# An Upper Bound Visualization of Design Trade‐Offs in Adsorbent Materials for Gas Separations: CO_2_, N_2_, CH_4_, H_2_, O_2_, Xe, Kr, and Ar Adsorbents

**DOI:** 10.1002/advs.202206437

**Published:** 2023-01-16

**Authors:** Samuel J. Edens, Michael J. McGrath, Siyu Guo, Zijuan Du, Hemin Zhou, Lingshan Zhong, Zuhao Shi, Jieshuo Wan, Thomas D. Bennett, Ang Qiao, Haizheng Tao, Neng Li, Matthew G. Cowan

**Affiliations:** ^1^ Department of Chemical and Process Engineering and MacDiarmid Institute for Advanced Materials and Nanotechnology University of Canterbury Canterbury 8041 New Zealand; ^2^ State Key Laboratory of Silicate Materials for Architectures Wuhan University of Technology Wuhan 430070 China; ^3^ Shenzhen Research Institute of Wuhan University of Technology Shenzhen 518000 China; ^4^ Department of Materials Science and Metallurgy University of Cambridge 27 Charles Babbage Road Cambridge CB3 0FS UK

**Keywords:** adsorbent design, adsorption, bound visualization, gas separation, metal organic framework, zeolite imidazolate framework

## Abstract

The last 20 years have seen many publications investigating porous solids for gas adsorption and separation. The abundance of adsorbent materials (this work identifies 1608 materials for CO_2_/N_2_ separation alone) provides a challenge to obtaining a comprehensive view of the field, identifying leading design strategies, and selecting materials for process modeling. In 2021, the empirical bound visualization technique was applied, analogous to the Robeson upper bound from membrane science, to alkane/alkene adsorbents. These bound visualizations reveal that adsorbent materials are limited by design trade‐offs between capacity, selectivity, and heat of adsorption. The current work applies the bound visualization to adsorbents for a wider range of gas pairs, including CO_2_, N_2_, CH_4_, H_2_, Xe, O_2_, and Kr. How this visual tool can identify leading materials and place new material discoveries in the context of the wider field is presented. The most promising current strategies for breaking design trade‐offs are discussed, along with reproducibility of published adsorption literature, and the limitations of bound visualizations. It is hoped that this work inspires new materials that push the bounds of traditional trade‐offs while also considering practical aspects critical to the use of materials on an industrial scale such as cost, stability, and sustainability.

## Graphical abstract

This critical review catalogues over 4000 adsorbent materials in a public database for the separation of CO_2_, N_2_, CH_4_, O_2_, H_2_ and noble gases. The tradeoffs between capacity, selectivity and heat of adsorption through a bound visualization are described. The design strategies used by the best outlier materials are highlighted, aiming to inspire the next generation of advanced porous materials.

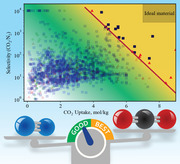

## 1 Introduction

Chemical separations account for 10–15% of the worlds energy use and are fundamental in the production of raw materials relied upon by modern society. For the separation and purification of gases, adsorption by solid materials is one process for performing chemical separations (alongside distillation and membrane technologies).^[^ ^1^ ^]^

The optimum process for a given separation or purification problem can be selected by consideration of several aspects: 1) Can the technique produce the required purity? 2) What proportion of the feed is recovered? 3) What are the operating costs associated with the desired purity and recovery? 4) What are the capital costs associated with the desired purity and recovery? Accurate consideration and comparison of the above questions will result in selection of an optimum separation/purification technique for a specific operational time horizon.

Key to the competitive success of an adsorption separation process is the selection of an adsorbent material with favorable separation characteristics for the gases of interest. For a fair assessment of separation performance, a material must be assessed under conditions that mimic its potential industrial application, which will almost always require process modeling.^[^ ^2^, ^3^, ^4^, ^5^ ^]^

Accurately assessing the performance of an adsorbent requires access to the adsorption isotherms under the temperature and pressure conditions of interest. Databases, such as the NIST database of Novel and Emerging Adsorbent Materials,^[^ ^6^ ^]^ provide pure and binary isotherm information for many materials. However, the database is not yet comprehensive and more than 70% of the initial 100 papers searched contained adsorbent information not in this database. A standard data format for the publication of adsorption isotherm information (AIF) has recently been proposed,^[^ ^7^ ^]^ which is similar to the well‐known crystallographic information file (CIF). The AIF has the potential to enable a more comprehensive isotherm database by sharing of electronic adsorption data, as opposed to relying on published figures.

Even where high‐quality and reliable isotherms are readily available, assessment of the separation performance of potentially hundreds of adsorbent materials through process modeling can be time‐intensive. Where isotherms are not available, obtaining (or synthesizing) the adsorbent and experimentally measuring isotherm data is even more time‐intensive.

A faster but less detailed and accurate approach to screening adsorbents is the comparison of key performance characteristics, such as capacity, selectivity, and heat of adsorption. This reduces the time to select promising adsorbents when reviewing references and provides a first‐glance estimation of their performance.^[^ ^8^ ^]^ Promising adsorbents can then be investigated further, finding isotherms from their original source or performing experimental measurements.

With an abundance of materials in the published literature (this work identified 1608 materials for CO_2_/N_2_ separation alone), selecting materials to use in a process model can be challenging. Several methods already exist to evaluate materials for use in a separation process. These methods include the ratio of effective purge amount to working capacity,^[^ ^9^ ^]^ the “PSA selection parameter”^[^ ^10^ ^]^ which considers selectivity and the working capacity for each gas and the “Adsorbent Performance Indicator” (API)^[^ ^11^ ^]^ which considers working capacity, selectivity and heat of adsorption, along with weighting factors relevant to the process. These methods are useful for comparing individual materials, but they have not been used to provide a comprehensive view of the field.

In 2021, we proposed using bound visualizations to compare the key properties (capacity, selectivity, and heat of adsorption) of adsorbents for alkene/alkane gas pairs.^[^ ^12^ ^]^ The bound visualization is ubiquitous in membrane science where the Robeson upper bound is used to compare the permeability and selectivity of membrane materials.^[^ ^13^, ^14^ ^]^

In the current work, we used a systematic method to source data from the literature (Section S1, Supporting Information) to expand application of the bound visualization to CO_2_/N_2_, CO_2_/CH_4_, CO_2_/H_2_, CH_4_/H_2_, CH_4_/N_2_, O_2_/N_2_, and the noble gases Ar/Xe/Kr from air. This application facilitates the identification and comparison of leading materials for 1) progression in understanding optimal design strategies for the development of superior adsorbent materials, and 2) further investigation through process modeling. In addition, the bound visualizations provide a visual overview that facilitates a description of what levels of capacity, selectivity, and heat of adsorption are relatively “good,” insight into the fundamental properties of adsorbent materials, rate of progress in the field, how leading works have advanced the field, what types of data collection and analysis provide the most relevant and impressive metrics, and the reproducibility of adsorbent production and isotherm measurement. This data is freely available for use at our website: https://adsorbents.canterbury.ac.nz to allow readers to create their own interactive bound plots and identify adsorbents of interest to their own research.

## 2 Data Collection

The detailed method used to identify and assess references for this review is contained in Section S1 (Supporting Information). Briefly, multiple Scifinder searches were used to collect data for this review and the resulting 4068 references were processed by hand, identifying 1051 references (4747 adsorbent material entries) with data useful to the review.

## 3 Bound Theory

Ideal adsorbent materials for gas separation would have a high capacity, high selectivity, and low heat of adsorption (discussion on additional factors including cost, stability, and sustainability is included in Section 17). However, there are limitations to optimizing all three parameters, as described in detail in our previous review that introduced the bound visualization.^[^ ^12^ ^]^

Briefly, for example, selectivity could be increased (favorable) by introducing active sites to interact specifically with the target gas, but this typically increases interaction strength and therefore increases heat of adsorption (unfavorable). Similarly, higher surface area could be introduced to increase capacity (favorable) but this typically introduces non‐selective surface area therefore reducing selectivity (unfavorable). For adsorbent materials, these trade‐offs form “bounds” that require new design strategies to surpass.

**Figure 1:**
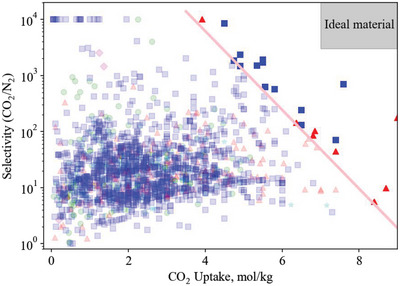
Example upper bound plot of CO_2_ uptake versus CO_2_/N_2_ selectivity. Each point is an adsorbent material, and the best materials lie in the upper‐right region of the plot above the upper bound, highlighted in solid colors.

A plot of capacity versus selectivity results in an upper bound (**Figure** **1**), where there are few materials that exhibit both high capacity and high selectivity. This upper bound illustrates the traditional trade‐off between capacity and selectivity, where capacity may be increased by increasing the pore volume or internal surface area, which often sacrifices gas‐specific interactions and reduces selectivity. The best materials can be identified above the upper bound line in the upper‐right region of the plot (Figure 1, highlighted in solid colors). Similarly, a plot of capacity versus heat of adsorption or selectivity versus heat of adsorption gives a lower bound, where few materials have a high capacity (or selectivity) and low heat of adsorption. In lower‐bound plots, the best materials lie in the lower‐right region below a lower‐bound line.

In this review, the upper and lower bounds are empirical observations based on the data gathered. While a physical explanation is sought for these empirical bounds (Section S2, Supporting Information), different phenomena can govern adsorption and therefore produce different bounds. For example, would perfect size‐sieving versus optimizing interactions lead to different fundamental limits? Would a core–shell structure be “cheating” the bound, or should it be considered alongside size‐sieving mechanisms?

Note that this paper reviews equilibrium data, and therefore uses the term “size‐sieving” to refer to a hypothetical adsorbent where narrow pore apertures would prevent access into pores under equilibrium conditions. This is distinct from kinetics limited processes where imperfect size‐sieving can achieve high kinetic selectivity based on gas size.

Herein, a linear axis is used for uptake (capacity) as would be expected from typical isotherm models such as the Langmuir isotherm which describes the loading as a certain proportion of occupied adsorption sites (Equation 1).

(1) $$\begin{array}{c} \theta_{A}=\frac{K_{A}p_{A}}{1+K_{A}p_{A}} \end{array}$$

Equation (1): This is the Langmuir isotherm for the adsorption of gas A on the surface of an adsorbent, where *θ*_A_ is the proportion of sites occupied by gas A, *K*_A_ is the equilibrium constant for the adsorption of gas A and *p*_A_ is the partial pressure of gas A.

In contrast to uptake, the selectivity for gas A over gas B can be considered as the ratio of their equilibrium constants (also known as affinity parameters). The temperature dependence of equilibrium constants is typically expressed as an exponential function of heat of adsorption (Equation 2).

(2) $$\begin{array}{c} K_{A}=K_{A,0}\exp\left(\frac{-\Delta H_{\mathrm{ads},A}}{R}\left(\frac{1}{T}-\frac{1}{T_{0}}\right)\right) \end{array}$$

Equation (2): This describes the temperature dependency of the Langmuir equilibrium constant of gas A (*K*_A_), as a function of temperature (*T*), where *K*_A,0_ is the equilibrium constant of gas A at *T*_0_, *R* is the universal gas constant and Δ*H*_ads,A_ is the heat of adsorption of gas A.

These equilibrium constants are exponential functions of heat of adsorption, and therefore are most suited to be plotted on a log axis. The wide range of selectivity values (spanning from 1 to 10000) also supports the use of a log axis.

In this paper, we provide the graphs with heat of adsorption on a linear axis both because the data covers a narrow region and is easier to read, and because we do not have an assertion that the axis should be on a log scale. Lower bound graphs with heat of adsorption as a log axis are provided in the Section S4 (Supporting Information).

### 3.1 Minimum Heat of Adsorption

**Figure 2:**
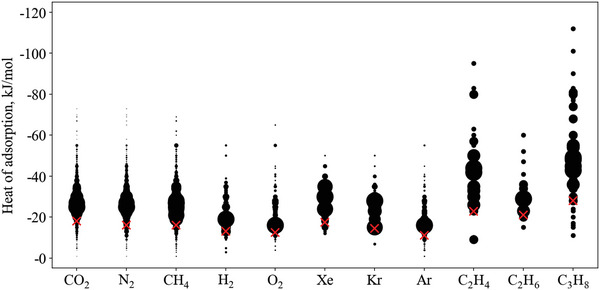
Distribution of heat of adsorption values for each gas (data gathered as described in Sections 7 and 19). A larger bubble indicates more materials at the given heat of adsorption value. Crosses indicate the 10^th^ percentile for the minimum heat of adsorption.

**Figure** **2** plots the heat of adsorption for each gas, where a larger marker corresponds to more materials. Data from the previous alkane/alkene review is included to give a broader view.^[^ ^12^ ^]^

The red crosses indicate the minimum heat of adsorption for each gas. This is defined as the value that more than 90% of materials report a higher heat of adsorption than. On the questionable assumption that enough data has been recorded and the physical limits have, or are close to, being reached, we hypothesize that a correlation should exist between the minimum heat of adsorption and physical properties of the gases.

To explore potential correlations, the minimum heat of adsorption value has been compared against polarizability, quadrupole moment, kinetic diameter, specific gas constant, and molar mass (Figures S6–S11, Supporting Information). No clear correlation emerged, with the closest correlation being to kinetic diameter, which shows a rough increasing trend (Figure S6, Supporting Information).

## 4 Results Overview

**Figure 3:**
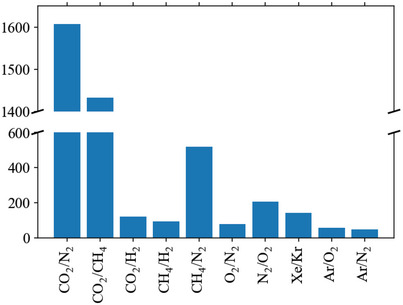
Number of adsorbent materials recorded for each gas pair, organized in the format selective gas/non‐selective gas. Less than 50 adsorbents for the Xe/O_2_/N_2_, Kr/O_2_/N_2_, Xe/Ar and Kr/Ar gas pairs were found, so are not included.

Our literature searches (method described in Section S1, Supporting Information) produced 4068 results, of which 1137 contained data suitable for inclusion in this review. The number of adsorbent materials recorded for each pair is shown in **Figure** **3**. Less than 50 adsorbents for the Xe/O_2_/N_2_, Kr/O_2_/N_2_, Xe/Ar and Kr/Ar gas pairs were found, so are not included. For all adsorbents identified, we recorded data for the three key screening properties: capacity, selectivity and heat of adsorption. These results can be viewed online at: https://adsorbents.canterbury.ac.nz where readers can create their own interactive bound plots and identify materials of interest.

## 5 Capacity

The most relevant measure of capacity (or uptake) to an industrial process is the working capacity of a material, in mol_gas_ kg^−1^_adsorbent_. The working capacity considers isotherm shape and temperature dependency to determine the difference between the amount of gas adsorbed under the temperature and pressure conditions at the end of an adsorption stage and the end of a desorption stage in a separation process. This differs from the capacity reported in isotherms, which are often recorded between 0 and 1 bar, because in real processes it is usually too energy intensive, and therefore uneconomical, to reach high‐vacuum conditions.

**Figure 4:**
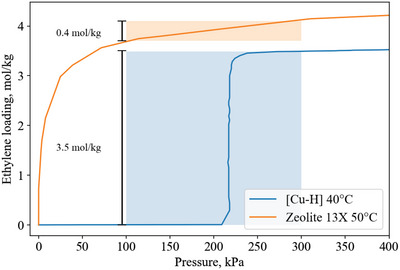
Type‐1 ethylene isotherm of Zeolite 13X^[^ ^15^ ^]^ compared to the step isotherm of nonporous [Cu—H].^[^ ^16^ ^]^ Zeolite 13X shows a loading of 4.1 mol kg^−1^ at 300 kPa, while [Cu‐H] has a loading of 3.5 mol kg^−1^ at the same pressure. This makes the capacity of Zeolite 13X appear better than [Cu‐H]. However, an example pressure swing between 300 kPa and 100 kPa reveals a working capacity of 0.4 mol kg^−1^ for Zeolite 13X, while a much larger working capacity of 3.5 mol kg^−1^ for [Cu—H]. This illustrates the difference between static capacity and working capacity.

The equilibrium isotherm capacity at 1 bar is not always the best indicator of working capacity. For example (**Figure** **4**), the Type‐1 ethylene isotherm of Zeolite 13X has a higher total capacity at 300 kPa (4.1 mol kg^−1^) than the step isotherm of ethylene on [Cu‐H] (3.5 mol kg^−1^). But if a pressure swing between 300 and 100 kPa is considered, Zeolite 13X only has a working capacity of 0.4 mol kg^−1^, while [Cu—H] has a much higher working capacity of 3.5 mol kg^−1^. Even for a vacuum‐pressure swing between 300 and 10 kPa, Zeolite 13X has a working capacity of 1.9 mol kg^−1^, while [Cu—H] still has 3.5 mol kg^−1^.

In a similar example, the Fe(bdp) and Co(bdp) frameworks have step‐shaped isotherms that provide working capacities ≥12 mol kg^−1^ at pressures ≥1 bar,^[^ ^17^ ^]^ which would make these materials more suitable for a pressure swing separation application, but register low capacity at 1 bar.

Recording the working capacity of a material requires the optimum swing conditions to be defined, which are different for every material, gas pair, and process. This made it impractical to record working capacity for each material in this review. Instead, the capacity reported from isotherms is recorded as close to 293 K and 100 kPa as possible. This is a quick assessment of material capacity, however, it is not as practically useful as a comparison of working capacities.

## 6 Selectivity

Selectivity is crucial to achieving the desired product purity in separation processes and optimizing the working capacity and productivity of an adsorbent material. Selectivity (binary selectivity) is reported in units of mol_selective gas_ mol^−1^_nonselective gas_.

In this review, the most common methods used for recording selectivity were the ideal adsorbed solution theory (IAST) and uptake ratio (selectivity as a ratio of the pure gas uptakes). IAST selectivity values were preferred and recorded instead of uptake ratio when a paper reported them. Consequently, most of the leading materials that will be identified reported selectivity using the IAST method because it accounts for mixed gas behavior where a higher selectivity is likely. A notable limitation is that these selectivity values represent equilibrium selectivity and do not consider kinetic selectivity.

Work by Walton and Sholl^[^ ^18^ ^]^ summarizes that IAST predictions are excellent for mixtures with small differences in size and/or adsorption interactions (such as CO_2_/CH_4_) but have more significant error for mixtures such as CO_2_/N_2_ where one component is much more strongly adsorbed than the other and the adsorbed phase deviates from an ideal mixture. This distinction is also observed in other work by Gharagheizi and Sholl^[^ ^19^ ^]^ that ranks gas mixtures in their ability to be predicted by IAST. Of the ten gas pairs, CO_2_/CH_4_ and CO_2_/N_2_ rank 7^th^ and 10^th^ respectively, while N_2_/O_2_, C_2_H_6_/C_2_H_4_ and N_2_/CH_4_ ranked in the top three.

Likewise, not all adsorbent materials conform with the assumptions of IAST. IAST requires that the specific surface area of the framework is constant and the same for all adsorbed species. For example, flexible materials do not fit this assumption as a result of the structural changes upon gate opening.^[^ ^20^ ^]^ As a result, IAST has the potential to overestimate their selectivity by up to two orders of magnitude.^[^ ^20^ ^]^

With CO_2_/N_2_ and CO_2_/CH_4_ being the most common mixtures in this review, there is a risk that “outliers” are identified due to the limitation of IAST predictions, rather than material performance. In limiting the impact of one obvious flaw, any selectivity reported >10 000 mol_selective gas_ mol_non‐selective gas_^−1^ was reduced to 10 000 mol_selective gas_ mol_non‐selective gas_^−1^. IAST predictions of such high selectivity are error prone because the application of IAST requires integration of the less favorably adsorbed isotherm to unphysically high pressures.^[^ ^19^, ^21^ ^]^

Finally, where pure isotherms are measured or modeled poorly, the multicomponent IAST prediction is poor, even if IAST is normally accurate for the gas/adsorbent system of interest.^[^ ^18^ ^]^

## 7 Heat of Adsorption

Adsorption is an exothermic process, leading to the release of energy (heat of adsorption) as gas is adsorbed. If this energy release is not managed through cooling, the temperature in a process will increase and reduce the capacity of the adsorbent material. The opposite is true for the desorption step in a process, which is endothermic. Desorption requires an energy input (typically heating) to “pay” the heat of adsorption required to regenerate the material. The energy use of an adsorption process will therefore be strongly related to the heat of adsorption, making low heat of adsorption desirable because it directly affects the minimum energy cost of an adsorption process. Additionally, adsorption processes can be limited by heat transfer rate, so reducing the amount of heating and cooling required (through reduced heat of adsorption) is beneficial to reducing the time between adsorption and desorption cycles.^[^ ^22^, ^23^ ^]^

**Figure 5:**
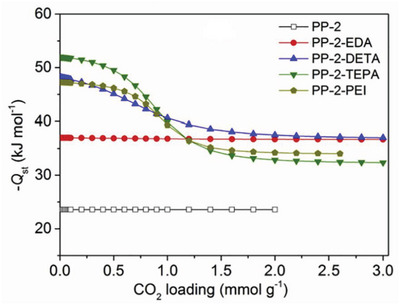
CO_2_ heat of adsorption as a function of CO_2_ loading on a range of amine‐modified porous polymers, illustrating how the value recorded at saturation capacity is not always representative of the entire range. For example, at 298K and 100 kPa, PP‐2‐PEI adsorbs 2.7 mol kg^−1^, giving a heat of adsorption of −34.5 kJ mol^−1^ and not accounting for the heat of adsorption up to −47 kJ mol^−1^. Reproduced with permission.^[^ ^24^ ^]^ Copyright 2019, Elsevier.

Heat of adsorption is a function of the amount of gas adsorbed. For example, **Figure** **5** shows heat of adsorption versus loading data for several variants of an amine‐modified porous polymer to demonstrate how the heat of adsorption can vary with loading.

PP‐2 and PP‐2‐EDA have a constant heat of adsorption, while the other materials have two distinct regions. The low loading region has a high heat of adsorption as a result of CO_2_ adsorbing via chemisorption to amine sites (a strong interaction). Once the amine sites are saturated (at higher loading), gas is adsorbed via physisorption to the material surface (a weaker interaction), resulting in a reduced heat of adsorption at higher loading.^[^ ^24^ ^]^ The flat regions of heat of adsorption indicate homogeneity of the surface adsorption sites, where their interaction strength with CO_2_ is uniform.^[^ ^24^ ^]^

In this review, only a single heat of adsorption value is recorded for each gas/material combination. This single value is chosen by choosing the heat of adsorption at the isothermal gas loading as close to 293 K/100 kPa as possible. The rationale for this is twofold: 1) To provide a consistent method for recording heat of adsorption across all reported literature. 2) Pressure swing processes usually operate at or above 1 bar.

Selecting a set condition to define heat of adsorption does not capture the entire range of data and is prone to under‐report the heat of adsorption. For example, PP‐2‐PEI has a CO_2_ capacity of 2.7 mol kg^−1^ at 298 K and 100 kPa and an associated heat of adsorption of −34.5 kJ mol^−1^ (Figure 5). This value is accurate for loadings from 1.5 to 2.7 mol kg^−1^ but under‐represents the increased heat of adsorption up to 47 kJ mol^−1^ at <1.5 mol kg^−1^. Ideally, this error would be accounted for by integrating the heat of adsorption over the working capacity range, requiring knowledge of the process‐specific adsorption and desorption conditions.

The exception to this situation is adsorbent materials that display “intrinsic heat management”, a phenomenon where the heat of adsorption is somewhat offset by a concurrent structural rearrangement (commonly associated with a step‐shaped isotherm). For example, this is seen for the Fe(bdp) and Co(bdp) frameworks which flex under CH_4_ pressures above 2 bar and reduce the net heat of adsorption (i.e., net heat of adsorption = heat of adsorption ‐ energy of structural rearrangement) to values ≤−8 kJ mol^−1^.^[^ ^17^ ^]^

This level of detail was impractical to consider for every material in this review, as the ideal and practical separation process conditions are not known. Instead, the single heat of adsorption value recorded is used as an indicator. When a material has an impressive (low) heat of adsorption value, we further examine the range of heat of adsorption values over areas of the isotherm with significant capacity change to determine if the material is truly an outlier or not. A material would be considered a true outlier if it maintains this low heat of adsorption value over pressures where gas uptake increases significantly.

## 8 Bound Parameters

As with the original Robeson upper bound used in membrane science, we fitted the adsorption bounds empirically, using the guiding principles based on physical limitations which are described below. **Tables** **1**, **2**, **3** outline the parameters used to describe these bounds. When referring to specific materials throughout this review, their properties are abbreviated to (uptake (mol kg^−1^), selectivity (mol mol^−1^), heat of adsorption (kJ mol^−1^)). For example, 2GrO@HKUST‐1^[^ ^25^ ^]^ for CO_2_/N_2_ separation has a capacity of 9.0 mol kg^−1^, a selectivity of 186 and a heat of adsorption of −25.5 kJ mol^−1^ and is abbreviated to (9.0,186,−25.5).

**Table 1:** Capacity (*q*) versus selectivity (*S*) empirical upper bound parameters (*a* and *b*), where *S* = *a*/exp(*bq*)

Gas pair	*a*	*B*
CO_2_/N_2_	3969017	1.617
CO_2_/CH_4_	1610262	1.588
CO_2_/H_2_	166555	1.060
CH_4_/H_2_	492	1.594
CH_4_/N_2_	52.88	1.134
O_2_/N_2_	100	3.542
N_2_/O_2_	34.74	1.400
Xe/Kr	160	0.577

**Table 2:** Capacity (*q*) versus heat of adsorption (*H*) empirical lower bound parameters (*m* and *c*), where *H* = *mq* + *c*

Gas pair	*m*	*c*
CO_2_/N_2_	3.78	0
CO_2_/CH_4_	3.78	0
CO_2_/H_2_	3.78	0
CH_4_/H_2_	12.50	0
CH_4_/N_2_	12.50	0
O_2_/N_2_	33.33	0
N_2_/O_2_	17.50	0
Xe/Kr	4.38	0

**Table 3:** Selectivity (*S*) versus heat of adsorption (*H*) empirical lower bound parameters (*g* and *f*), where *H* = *g*ln (*S*) + *f*, including the horizontal bound at low selectivity and constant heat of adsorption

Gas pair	*g*	*f*	Min Δ*H*_ads_ [kJ mol^−1^]	Min Δ*H*_ads_ from *S* = 1 to *S* = …
CO_2_/N_2_	10.86	−40	10	100
CO_2_/CH_4_	11.94	−17.50	10	10
CO_2_/H_2_	8.69	−16.02	10	20
CH_4_/H_2_	9.38	−16.09	12	20
CH_4_/N_2_	12.43	−5.23	12	4
O_2_/N_2_	13.10	4.69	10	1.5
N_2_/O_2_	6.51	10.00	n/a	n/a
Xe/Kr	9.52	−0.463	10	3

The capacity versus heat of adsorption bound intercepts zero for all gas pairs, with the reasoning that if there is no gas uptake, there is no gas–solid interaction and therefore, no heat of adsorption. The positioning of this bound is only dependent on the selective gas (i.e., it is identical for CO_2_/N_2_, CO_2_/CH_4,_ and CO_2_/H_2_ gas pairs), because the bound visualization only plots the pure component capacity and heat of adsorption of the selective gas.

The selectivity versus heat of adsorption bounds feature a horizontal minimum heat of adsorption before reaching a selectivity where a tradeoff is observed between selectivity and heat of adsorption. This minimum heat of adsorption value is tied to the selective gas, so is identical for all gas pairs with the same selective gas. The tradeoff between selectivity and heat of adsorption likely occurs because selectivity is typically increased by introducing active sites or modifying pores to more strongly interact with the desired gas–this increase in interaction strength increases heat of adsorption.

## 9 Carbon Dioxide—Nitrogen

**Figure 6:**
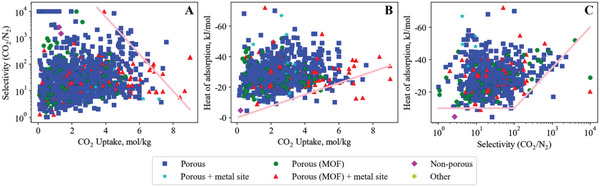
Carbon dioxide – nitrogen empirical bounds illustrating the trade‐off between A) CO_2_ uptake versus CO_2_/N_2_ selectivity (upper bound), B) CO_2_ uptake versus CO_2_ heat of adsorption (lower bound) and C) CO_2_/N_2_ selectivity versus CO_2_ heat of adsorption (lower bound). The legend applies to all figures in this manuscript, unless stated otherwise.

Our search method identified 1608 materials studied for adsorption of the CO_2_/N_2_ gas pair, which form upper and lower bounds illustrating the tradeoffs in adsorbents for CO_2_/N_2_ (shown in **Figure** **6**). The bounds are apparent by eye, with only a few standout adsorbents surpassing the bounds and making themselves apparent for analysis to identify design strategies success for optimizing a pair, or even all three, of the key properties. All three bounds are important to consider when selecting a material. However, the specific application may place more importance on one bound over another. For example, a bulk separation with high feed flow rate will likely prioritize uptake capacity (Figure 6A,B) to minimize the amount of adsorbent (and therefore equipment volume) required.

Key physical parameters (Table S1, Supporting Information) of the gases affect the sorption properties and can guide strategies for material design. For CO_2_ and N_2_, the respective kinetic diameters (3.3 and 3.64 Å), polarizabilities (26.5 × 10^−25^ and 17.6 × 10^−25^ cm^3^) and quadrupole moments (4.3 × 10^−26^ and 1.52 × 10^−26^ esu cm^2^) suggest design strategies that exploit size‐sieving or an increase interaction strength with CO_2_ could be suitable strategies.

**Figure 7:**
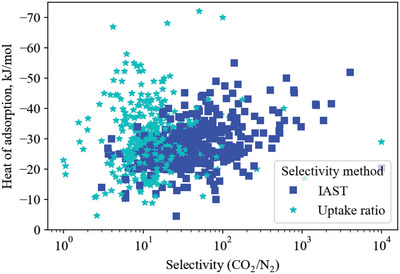
Comparison of CO_2_/N_2_ selectivity values reported using the IAST method (blue squares) and the uptake ratio method (cyan stars), illustrating that IAST predictions tend to report higher selectivity values than the uptake ratio.

Before diving into analyzing the bounds and the materials that make and exceed them, is it important to highlight that our methodology and its limitations are included at the end of this article (Section 19) and in Section S1 (Supporting Information). One important point to include here is that selectivity values were preferentially taken from IAST calculations where available because they better represent selectivity in a real separation process compared to a simple ratio of pure gas uptake. This leads to a large apparent discrepancy between the selectivity of materials which report IAST selectivity versus those which only report uptake ratios (illustrated in **Figure** **7**).

Zeolite 13X was one of the first adsorbent materials studied for the recovery of CO_2_ from flue gas and serves as a good example for noting the aforementioned limitations in our analysis method.^[^ ^9^ ^]^ Zeolite 13X appeared in a 1995 publication where its performance was studied in a pressure‐swing‐adsorption system applied to capturing CO_2_ from 16% and 26% CO_2_/N_2_ mixtures, achieving CO_2_ purities >99% and recoveries of 53% and 70%, respectively.^[^ ^9^ ^]^

Surprisingly, given the >1500 materials studied, the initial report of Zeolite 13X remains beyond the CO_2_/N_2_ lower bound for heat of adsorption (−40 kJ mol^−1^ vs selectivity (1850, IAST) (Figure 6C) [We note “the initial report” referring to Section 16 Reproducibility]. As a material, it provides a good first example for illustrating the discrepancy between IAST (CO_2_/N_2_ = 1850, at 1.1 bar, 15 °C) and selectivity estimated by taking the uptake ratio of single‐gas isotherms (CO_2_/N_2_ = 6.5).

### 9.1 Breaking the CO_2_/N_2_ Selectivity versus Uptake Upper Bound

The CO_2_/N_2_ selectivity versus uptake plot (Figure 6A) shows a clear trade‐off between uptake and selectivity, where high selectivity is only achieved at reduced CO_2_ uptake. The highest capacity materials on this bound include Mg‐MOF‐74 (9, 175, −34),^[^ ^26^ ^]^ Graphene‐oxide HKUST‐1 composite (9, 186, −26),^[^ ^25^ ^]^ and some reports of MOFs HKUST‐1 (8.7, 9.8, N/A)^[^ ^27^ ^]^/ (6.9, 103, −23)^[^ ^25^ ^]^/(7.4, 44, N/A),^[^ ^28^ ^]^ and CPO‐27‐Ni (Ni‐MOF‐74) (8.4, 5.6, −40)^[^ ^29^ ^]^/(6.8, 85, −38).^[^ ^30^ ^]^

The performance of Mg‐MOF‐74 (9,175,‐34)^[^ ^26^ ^]^ embodies two design strategies that feature heavily amongst materials that break the bounds. The pores in Mg‐MOF‐74 contain open metal ion sites that provide a strong binding site for CO_2_. The relatively high prevalence of these sites as a proportion of the framework structure ensures the CO_2_ uptake is among the highest reported. The next nuance here is size and shape of the pores, such that the orientation and packing of CO_2_ onto the open metal sites effectively fills the total available pore volume—leaving no non‐selective surface area for nitrogen to access. This latter feature achieves the high selectivity, which combined with high capacity makes Mg‐MOF‐74 a bound‐breaking material. Similarly, some reports of HKUST‐1 and Ni‐MOF‐74 feature on the bound.

The high capacity and selectivity of graphene‐oxide HKUST composites (9, 186, −26)^[^ ^25^ ^]^ have been attributed to the void space created at the HKUST–graphene interface. As a caution, the IAST method assumes the adsorbent is homogeneous,^[^ ^18^ ^]^ and was applied on this composite material. This adsorbent suggests that one strategy toward high‐performance adsorbents is to purposefully introduce interfacial and interstitial defects that have properties surpassing their “perfect” crystalline analogs. This explanation is a design motif we observe used by core–shell materials for breaching both the heat of adsorption bounds.

A single work lists multiple ion‐exchanged FAU‐type zeolites (‐X and Li‐LSX types) modified by an aqueous ammonia treatment to produce ABW and ANA‐type structures, with best performing materials being Li‐LSX‐100‐24 (4.5,8400,‐N/A), Na‐X‐150‐24 (5.5,1900,‐N/A) and Na‐X‐200‐24 (5.5,1700,‐N/A).^[^ ^31^ ^]^ Although the reason for the exceptional performance is unclear, this series of materials has among the best trade‐offs for capacity versus selectivity. Furthermore, a systematic study combining the Na‐X zeolite with different binder materials showed that the properties of these zeolites can be maintained when the adsorbents are pelletized for use in a PSA process, e.g., NZL‐500 (5.6,620,‐N/A).^[^ ^32^ ^]^

### 9.2 Breaking the CO_2_/N_2_ Heat of Adsorption versus Uptake Lower Bound

The CO_2_/N_2_ uptake versus heat of adsorption plot (Figure 6B) shows a clear trade‐off between uptake and heat of adsorption. Note that the information in this bound line describes CO_2_ only, so the bound is identical for the other CO_2_ gas pairs (CO_2_/CH_4_ Figure 9B and CO_2_/H_2_ Figure 10B).

The simplest design strategy for exceeding the heat of adsorption versus uptake lower bound is to produce high surface area materials with weak adsorption sites. The materials in this category include activated and nitrogen‐doped carbons, including: KNWS‐2‐600‐120 (7.4,70,‐25),^[^ ^33^ ^]^ NPC‐2‐700 (6.8,12,‐23),^[^ ^34^ ^]^ and, PMF Carbon cryogel 1:2.5 (5.7,5.8,‐10),^[^ ^35^ ^]^ which are produced from a variety of cheap sources. As expected for this class of materials, the CO_2_/N_2_ selectivities are relatively low.

The metal organic frameworks that sit on or beyond this bound include reports of HKUST‐1 (6.9,103,‐23)^[^ ^25^ ^]^ and a graphene oxide composite 2GrO@HKUST‐1 (9,186,‐26),^[^ ^25^ ^]^ and Ni‐MOF‐74 (4.5,35,‐17)/(5.5,32,‐20)^[^ ^36^ ^]^ synthesized under hydrothermal and microwave conditions, respectively. Mg_2_(dobdc) (Mg‐MOF‐74) (9175,‐34) also features,^[^ ^26^ ^]^ and the cobalt‐based MOF LCU‐105a (4.9,12.5,‐18)^[^ ^37^ ^]^ is close to the bound and discussed in depth in the CO_2_/CH_4_ section. These frameworks illustrate the limitation of using standard conditions for this bound visualization, because the majority of gas uptake occurs at pressures below 1 bar and the heat of adsorption in that region of the isotherm, i.e., associated with the uptake, is significantly higher than the value recorded at 1 bar and plotted on the bound.

The composite core–shell variants of Zeolite 5A and Ni‐MOF‐74 also sit beyond the bound [ZeoB@MOF‐74 (7,9.3,‐12.5) and ZeoA@MOF‐74 (7.4,9.3,‐13)].^[^ ^38^, ^39^ ^]^ Their selectivities are much lower than the MOF‐74 parent materials, which is due to mesopores (i.e., non‐selective free space) at the interfacial boundary between the MOF‐74 and zeolite layers.

Notably close to the bound is the nanoporous nitrogen‐doped carbon material Co‐NDPC‐600/700 (5.3,84,‐19)/(5.6,212,‐18).^[^ ^40^ ^]^ These high‐surface area materials contain low‐energy adsorption sites as a result of the cobalt ions and nitrogen‐doped into the material during its synthesis and pyrolysis. The material stands out for the amount of nitrogen successfully retained in the carbon material (>11 wt%), i.e., a large number of adsorption sites. Achieving the largest amount of nitrogen into an activated carbon material while maintaining a suitable porous structure appears to be the key design challenge for this class of materials.

### 9.3 Breaking the CO_2_/N_2_ Heat of Adsorption versus Selectivity Lower Bound

The CO_2_/N_2_ heat of adsorption versus selectivity lower bound (Figure 6C) is horizontal, reflecting a minimum CO_2_ heat of adsorption, until a CO_2_/N_2_ selectivity of ≈ 100 is reached. Up to 2015, the sloped bound began at a selectivity of ≈ 15, then from 2015–2018 the sloped bound start point extended to ≈ 100. Past a CO_2_/N_2_ selectivity of 100, a clear bound appears that correlates to a trade‐off of increasing selectivity requiring higher CO_2_ heat of adsorption.

Materials that “break” the horizontal minimum adsorption energy fall into two categories. The first highlights a notable limitation in collecting the heat of adsorption data, where the heat of adsorption observed at standard conditions (298 K, 100 kPa) does not reflect the much higher heat of adsorption at low pressures/low sorbent loadings. This also includes most materials at the edge of the bound, which are porous adsorbents that have reached or approached saturation capacity and therefore only weak adsorption sites remain.

An example is the series of porous PMF cryogels,^[^ ^35^ ^]^ created by pyrolysis of phenol, melamine, and formaldehyde mixtures. For the PMF series, the heat of adsorption decreases from −35 kJ mol^−1^ at low CO_2_ loadings to −10 kJ mol^−1^ at high CO_2_ loadings. The second case includes chitosan–SH (0.2,2.6,‐4.7)^[^ ^41^ ^]^ and thermally reduced graphite (0.59, 26, −4.5),^[^ ^42^ ^]^ both nonporous polymers with low CO_2_ capacity.

Materials that break the sloped section of the bound achieve size‐sieving that prevents the entry of nitrogen. AlOF (3.9,10000,‐20)^[^ ^43^ ^]^ is a true outlier because along with a high selectivity, its low heat of adsorption is maintained across a range of loadings. AlOF is a rigid framework with 1D channels that limit access into the internal pore spaces. Those authors theorized that strong nitrogen adsorption onto sites in the 1D channels blocked further adsorption of nitrogen gas. In contrast, CO_2_ appeared able to traverse these channels and access weak hydrogen binding adsorption sites in the internal pore space. This restriction of nitrogen entry is a remarkable accomplishment, apparently made possible by the 1D channels and rigidity of the channels.

### 9.4 Leading Designs for Selective CO_2_/N_2_Adsorption

The M‐MOF‐74 series of metal organic frameworks appear beyond or at all three of the CO_2_/N_2_ bounds. The reason for this success is a combination of strong interaction sites (open metal ions) and pore sizes that are close to perfectly filled when CO_2_ occupies the high‐strength binding sites. This theme of pore size‐matching is evident throughout the rest of the leading material sections for most gas pairs.

Positioning along the capacity versus selectivity upper bound can be achieved by modulating the interaction strength of adsorption sites, as seen in the series of ion‐exchanged FAU‐type zeolites. Another prominent design strategy was the size‐sieving achieved by the adsorbent AlOF, which achieved very high selectivity with low heat of adsorption. The careful crystal design and engineering to achieve perfect CO_2_/N_2_ size‐sieving of internal pore space has the potential to produce high‐performance adsorbent materials and should be explored further.

### 9.5 CO_2_/N_2_ Bounds over Time

**Figure 8:**
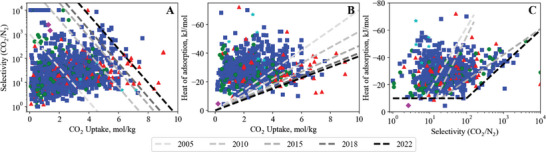
CO_2_/N_2_ bound movement from 2010 to the current day—A) uptake versus selectivity, B) uptake versus heat of adsorption, and C) selectivity versus heat of adsorption. Symbols represent material class; Square—porous, star—porous with metal site, circle—MOF,triangle—MOF with metal site, diamond—nonporous. In C), the 2018 bound cannot be seen as it did not move from 2018 to 2022.

Plotting the position of the bounds from 2005 to 2022 shows the evolution of adsorbent materials over time (**Figure** **8**). This evolution is clearer in the GIFs included as Supporting Information. The majority of these materials were published as the MOF field grew after ca. 2005, which we infer also correlates to increased scientific interest in materials to address climate change and a focus on capturing carbon dioxide from flue gas mixtures.

Both MOFs and porous materials have pushed the uptake versus selectivity bound forward, with metal site MOFs having a greater presence at the bound than MOFs without metal sites. Metal site MOFs are more present at the high uptake/lower selectivity end of the bound, while other porous materials have the majority at the lower capacity/higher selectivity end of the bound.

## 10 Carbon Dioxide—Methane

**Figure 9:**
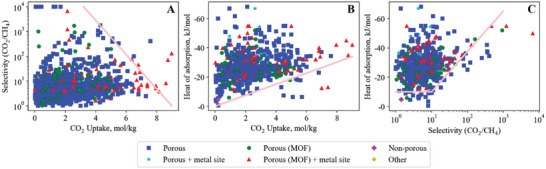
Carbon dioxide–methane empirical bounds illustrating the trade‐off between A) CO_2_ uptake versus CO_2_/CH_4_ selectivity (upper bound), B) CO_2_ uptake versus CO_2_ heat of adsorption (lower bound) and C) CO_2_/CH_4_ selectivity versus CO_2_ heat of adsorption (lower bound).

Our search method identified 1432 materials studied for adsorption of the CO_2_/CH_4_ gas pair. Similar to CO_2_/N_2_, the majority of these publications were published after ca. 2005 and exhibit visually apparent upper and lower bounds (**Figure** **9**).

The CO_2_/CH_4_ gas pair is of primary interest for the production of high‐quality natural gas streams where CO_2_ must be removed from a mixture composed mostly of CH_4_.^[^ ^44^ ^]^ Liquid amine absorption is the established method for this separation, with processes licensed by Fluor, Shell, DOW and many others.^[^ ^45^ ^]^ Pressure‐swing adsorption (PSA) using solid‐state sorbents for natural gas purification has drawn research attention because it is feasible even at small scales, does not require handling of corrosive chemicals and requires less energy than liquid absorption.^[^ ^44^ ^]^

The kinetic diameters (3.3 and 3.8 Å), polarizabilities (26.5 × 10^−25^ and 26 × 10^−25^ cm^3^) and quadrupole moments (4.3 × 10^−26^ and 0 esu cm^2^) of CO_2_ and CH_4_ respectively, suggest that design strategies that exploit size‐sieving or an increase interaction strength with the quadrupole moment CO_2_ will be suitable, while their similar polarizabilities remove this as an avenue for separation (see Table S1, Supporting Information for a complete comparison of physical parameters).

### 10.1 Breaking the CO_2_/CH_4_ Selectivity versus Uptake Upper Bound

The CO_2_/CH_4_ selectivity versus uptake plot (Figure 9A) shows a clear trade‐off between uptake and selectivity, where high selectivity is only achieved at reduced CO_2_ uptake.

Mg‐MOF‐74 variants exclusively dominate the high uptake end of the bound. Also known as Mg_2_(dobdc) and CPO‐27‐Mg, these frameworks incorporate coordinatively‐unsaturated metal sites in the form of magnesium,^[^ ^30^, ^46^, ^47^, ^48^, ^49^ ^]^ nickel,^[^ ^30^, ^49^ ^]^ manganese,^[^ ^30^ ^]^ and cobalt/zinc.^[^ ^30^, ^50^ ^]^ The dominance of this framework with reports from several different authors cements it is an impressive choice for maximizing selectivity and capacity.

When following these discussions, please note that several reports of the magnesium MOF‐74 variants illustrate the points made in Section 16—Reproducibility. CO_2_ uptake at 298 K/100 kPa varied from 6.4 to 9 mol kg^−1^, while IAST selectivity varied from 70 to 130 mol mol^−1^.

Both physical experiments and molecular modeling provide insight into understanding the exemplary performance of the Mg‐MOF‐74 variants. The open metal sites achieve bonding to multiple CO_2_ molecules (e.g., 1.75 CO_2_ per Mg ion in that variant)^[^ ^51^ ^]^ which results in an arrangement of CO_2_ molecules that leaves very little unoccupied void space within the pores and on the interior surface of the pore walls. These frameworks therefore maximize the ratio of selective‐adsorption sites over non‐selective void space, leading to their position at or above the selectivity versus uptake upper bound.

As seen in Section 10.2, Mg‐MOF‐74 also sits along the heat of adsorption versus capacity lower bound, making it a rare material that sits beyond or close to all three bounds. Note that this is true of data from two of its reports,^[^ ^30^, ^46^ ^]^ whereas two others report properties well within the bound.^[^ ^47^, ^49^ ^]^

The high selectivity region is dominated by Zeolite A and X variants either unmodified^[^ ^52^, ^53^ ^]^ or modified by ion‐exchange with potassium/cesium^[^ ^53^ ^]^ or lithium.^[^ ^54^ ^]^ Likewise, just inside the bound are ion‐exchanged KFI and CHA zeolite variants.^[^ ^55^, ^56^ ^]^ For potassium and cesium ion‐exchanged zeolite A and X frameworks,^[^ ^53^ ^]^ the introduction of the larger cesium ions is accompanied by major increases in selectivity without compromising pore volume and capacity. We go beyond the author's initial interpretation to suggest that the presence of the larger cesium ions contributes to blocking defects within the crystalline structure and forcing transport through the pore apertures with sizes controlled by the potassium ions. This is an important point worthy of further study, as it suggests that ion exchange is a potential route to filling the defects in “perfect” crystalline molecular sieving materials.

It must be noted that the promising zeolites were limited to three different reports,^[^ ^52^, ^53^, ^54^ ^]^ with >40 reports of other ion‐exchanged zeolites that sit well within the bound, so this is not a guaranteed method to balancing trade‐offs and requires close matching of ion and cavity sizes. In contrast, the only MOF‐74 reports within the bound were the Ni‐MOF‐74 variant.^[^ ^57^, ^58^, ^59^ ^]^ The significant differences in selectivity and uptake in these MOF materials with relatively small changes in bond lengths illustrates the fine structural control required for exceptional performance.

A core–shell composite structure of Zeolite 5A and Ni‐MOF‐74^[^ ^38^, ^39^ ^]^ synthesized using the seed‐mediated growth method also sits near the bound (7.4,7.4,‐13), combining the two major materials groups that sit near the bound. As reported above, this material incorporated 5wt% Zeolite 5A and increased the capacity compared to Ni‐MOF‐74 alone as a result of new mesopores at the MOF–zeolite interface. It is therefore the ability to include non‐selective volume/surface area (in this case the empty space of the mesopores), averaging out the heat of adsorption and selective adsorption provided by the Zeolite and Ni‐MOF‐74 that allows this material to exceed the bound.

### 10.2 Breaking the CO_2_/CH_4_ Uptake versus Heat of Adsorption Lower Bound

Figure 9B shows a clear trade‐off between CO_2_ uptake and heat of adsorption. All materials that overcome this bound are nitrogen‐doped carbons,^[^ ^33^, ^34^, ^40^, ^60^ ^]^ with two exceptions. The prevalence of nitrogen‐doped carbons reflects that selectivity is not taken into account for this bound. As shown in **Table** **4**, all adsorbents have selectivities for CO_2_/CH_4_ that are <12 and rely on high surface areas with minimal CO_2_‐specific interactions. As such, while minor improvements may be possible, this strategy does not provide a path toward overcoming the limits of the current bound.

**Table 4:** Comparison of adsorption performance and nitrogen content of carbon materials that overcome the CO_2_/CH_4_ uptake versus heat of adsorption lower bound

Material	Temp [K]	Capacity at 100 kPa [mol kg^−1^]	CO_2_/CH_4_ selectivity [mol mol^−1^]	CO_2_ heat of adsorption [kJ mol^−1^]	*N* [wt%]
KNWS‐6‐750‐90^[^ ^33^ ^]^	298	4.4	4.5	‐13	n/a
Co‐NDPC‐600^[^ ^40^ ^]^	298	5.3	11.4	–19	11.74
Co‐NDPC‐700^[^ ^40^ ^]^	298	5.6	7.1	–18	10.86
NPC‐2‐700^[^ ^34^ ^]^	273	6.8	2.9	–23	1.70
CP‐AC^[^ ^60^ ^]^	298	4.5	3	–7.4	0.53
J‐AC^[^ ^60^ ^]^	298	5.7	4.2	–6.5	2.31
K‐AC^[^ ^60^ ^]^	298	5.1	6.8	–6.4	2.12

Of the two exceptions, the first is a cobalt‐based MOF with dual active sites, LCU‐105a.^[^ ^37^ ^]^ LCU‐105a (4.9,7,‐18) is an anionic framework with loose excess carboxylate functionality where the sodium ions are exchanged for dimethylammonium cations, [NH_2_(CH_3_)_2_]^+^. Grand Canonical Monte Carlo simulations provided evidence that CO_2_ interacts electrostatically with the negatively charged carboxylates in this framework. The example of LCU‐105a therefore provides an example where the use of weak, but still selective, electrostatic interactions rather than stronger open metal site interactions can be used to increase CO_2_ capacity with minor consequence to heat of adsorption, effectively providing a design strategy to produce materials along the mid‐range of the current uptake versus heat of adsorption bound.

The second exception is the Zeolite 5A/Ni‐MOF‐74 composites discussed in the uptake versus selectivity bound which rely on mesopores at the Zeolite/MOF‐74 interface to also break the uptake versus heat of adsorption bound.^[^ ^38^, ^39^ ^]^

Zeolite 13X^[^ ^61^ ^]^ also features outside of the bound, but this report appears to be an outlier, given the number of Zeolite 13X reports that do not break the bound (see Section 16 Reproducibility). Mg‐MOF‐74 also features close to the bound, seen as the four red triangles at ca. 8 mol kg^−1^ in Figure 9B.

### 10.3 Breaking the CO_2_/CH_4_ Heat of Adsorption versus Selectivity Lower Bound

The CO_2_/CH_4_ heat of adsorption versus selectivity lower bound (Figure 9C) is horizontal, reflecting a minimum CO_2_ heat of adsorption of 10 kJ mol^−1^, until a CO_2_/CH_4_ selectivity of ca. 10 is reached. Past a CO_2_/CH_4_ selectivity of 10, a linear bound appears that correlates to a trade‐off of increasing selectivity requiring higher CO_2_ heat of adsorption.

Of the materials that sit below the 10 kJ mol^−1^ horizontal bound, none exhibit CO_2_‐specific interactions. Some report a heat of adsorption greater than 10 kJ mol^−1^ at low loading (Rb‐MER‐2.3,^[^ ^62^ ^]^ SBA‐15,^[^ ^63^ ^]^ NUT‐12^[^ ^64^ ^]^ and cellulose fiber carbons^[^ ^60^ ^]^), some rely on heat of adsorption from temperature dependent isotherm model fits instead of the methods like Clausius‐Clapeyron (ACI granules^[^ ^65^ ^]^ and IFP‐7^[^ ^66^ ^]^) and most others have a capacity less than 0.5 mol kg^−1^ (Rb‐MER‐2.3,^[^ ^62^ ^]^ Chitosan‐SH,^[^ ^41^ ^]^ coals,^[^ ^67^, ^68^ ^]^ MWCNTs,^[^ ^69^ ^]^ CTF‐NH^[^ ^70^ ^]^ and SBA‐15^[^ ^63^ ^]^).

Only MOFs display both very high selectivity and relatively low heat of adsorption. These include ZU‐36‐Ni (2.6,930,‐52)^[^ ^71^ ^]^ and MUF‐16 variants using cobalt (2.1,6690,‐50),^[^ ^72^ ^]^ manganese (2.1,470,‐55)^[^ ^72^ ^]^ and nickel centers (2.1,1220,‐55).^[^ ^72^ ^]^ These materials have pore or channel sizes that match the gas molecule of interest and contain many guest‐specific interactions on the framework wall, minimizing the nonselective surface area. For example, MUF‐16 variants^[^ ^72^ ^]^ match the size of CO_2_ to the 1D channels that run through the framework. This results in optimized interactions between CO_2_ and the framework wall that do not occur with smaller hydrocarbon molecules. ZU‐36‐Ni (2.6,930,‐52)^[^ ^71^ ^]^ (also named GeFSIX‐3‐Ni) also beats the bound and displays a similar design strategy. The pores are closely sized to CO_2_ (3.3 Å) through selection of the metal ion to control the metal–ligand bond length, and the CO_2_‐specific interactions in this case are provided via fluorine atoms on the GeF_6_^2−^ anions within the pore structure.

Matching pore size to the adsorbed gas molecule (or a number of gas molecules suitable for completely filling the pore) is a recurring theme throughout upper bound‐breaking materials. The combination of densely loading the wall with guest‐specific interactions and minimizing nonselective surface area and unoccupied volume results in high selectivity. Note that this is distinct from attempting to achieve size‐sieving, and does not require the same stringent control over the thermal flexibility of the pore size. However, the unavoidable trade‐off of relying on strong interactions is an increase in heat of adsorption.

SBA‐15 (mesoporous silica) has been reported to break the lower bound when grafted with specific amounts of amino‐silanes.^[^ ^63^ ^]^ These adsorbent materials are reported to show a range of selectivities (106‐374) and heats of adsorption (−22 to −44 kJ mol^−1^) outside the bound, noting that the heats of adsorption increase up to −80 kJ mol^−1^ at lower loadings. Even so, the inclusion of amine functionality usually pairs the adsorbent material with a high heat of adsorption.

The NUT series of microporous polymers sit outside the bound [e.g., NUT‐6 (3.6,22,‐18)], and along the bound [e.g., NUT‐5 (1.4,11,‐11)]^[^ ^73^ ^]^ The NUT series of materials are poly(aromatics) and have no CO_2_‐specific interactions, achieving the low heats of adsorption. The authors attribute the CO_2_ selectivity to micropores with pore diameter <1 nm, however CO_2_ (3.3 Å), N_2_ (3.64Å), and CH_4_ (3.8 Å) are all well under that size. The authors also suggest the cross‐linking density and cross‐linking length are also important, but exactly how remains unclear. These materials may operate on a similar design principle to core–shell materials (discussed above in Sections 10.1 and 10.2) that introduce mesopores into the adsorbent. Overall, the experimental data is consistent with a completely organic material that sits below the lower bound.

Similarly, adsorbents with core–shell morphology can also exceed the heat of adsorption versus selectivity bound. The shell structure can be optimized for selectivity while the core can be optimized for high capacity and minimal heat of adsorption (e.g., weak interactions). For example, Zeolite 4A (4,5.6,‐33) sits well within the bound, but after silica coating via chemical liquid deposition (CLD), its selectivity increased significantly (2.9,76,‐33) to place it outside of the bound.^[^ ^74^ ^]^ A selectivity of 76 is calculated using the uptake ratio method, as this type of core–shell material is not suitable for evaluation with the IAST method. The silica layer controlled the external pore apertures to almost completely exclude CH_4_ from the internal zeolite cavities (which remain unaltered). The modified material shows almost no methane uptake (reduced from ca. 0.7 mol kg^−1^ at 298 K before modification). The only downside to the silica layer was a reduction in CO2 uptake from 4 to 2.9 mol kg^−1^.

Drawing on a similar material for comparison, the composite of Zeolite 5A and Ni‐MOF‐74 also performed well, being on the uptake versus selectivity bound and breaking the uptake versus heat of adsorption bound.^[^ ^38^, ^39^ ^]^ However, this composite does not balance selectivity versus heat of adsorption and sits well within this bound. It is clear that for this core–shell strategy to be effective on all three bounds, the inner core should have low heat of adsorption and high capacity—relying on the shell to provide the needed selectivity.

### 10.4 Leading Designs for Selective CO_2_/CH_4_ Adsorption

The leading materials have consistently displayed the design feature of pores or channels sized to the CO_2_ molecule(s) leaving minimal non‐selective void or surface area available for other gases to occupy. In addition to its good performance on the bounds, MUF‐16 was reported for good moisture and air stability, mixed‐gas (though not humidified) behavior, along with commentary on (relatively low) cost and ease of manufacture.

The core–shell method of silica‐coated Zeolite 4A is an illustration of how a cheap porous material with good capacity and low heat of adsorption can be modified to increase selectivity. In this design strategy, the key challenge appears to be developing coating methods that retain as much capacity as possible. While activated carbons combine high capacity with low heat of adsorption, a challenge would be coating these materials with the core–shell or other strategies.

Overall, for CO_2_/CH_4_ we have observed different classes of materials coming to the fore over different sections of the bounds. Close matching of cavity size to CO_2_ has produced the best materials to date and requires careful molecular design of the adsorbent materials. In contrast, strategy of producing core–shell composite materials, or introducing microporosity such as the NUT series, is readily generalizable and may provide routes to cheaper or more chemically stable adsorbents given the range of materials that could be used to produce core–shell (inorganic/organic composites) and microporous (organic) structures.

## 11 Carbon Dioxide—Hydrogen

**Figure 10:**
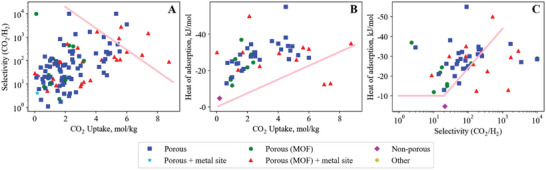
Carbon dioxide–hydrogen empirical bounds illustrating the trade‐off between A) CO_2_ uptake versus CO_2_/H_2_ selectivity (upper bound), B) CO_2_ uptake versus CO_2_ heat of adsorption (lower bound) and C) CO_2_/H_2_ selectivity versus CO_2_ heat of adsorption (lower bound).

Our search method identified 121 materials studied for adsorption of the CO_2_/H_2_ gas pair (**Figure** **10**). Hydrogen has been identified as an important part of the future energy supply, with the ability as an energy carrier to generate heat and electricity without emissions at the point of use.^[^ ^75^ ^]^

Hydrogen can be produced in a variety of ways, including natural gas reforming, gasification, and electrolysis.^[^ ^76^ ^]^ Around 95% of all hydrogen is produced from steam reforming of natural gas where the water‐gas shift reaction is used to increase the yield of hydrogen. Pressure‐swing adsorption is then typically used to remove carbon dioxide and other contaminants to produce pure hydrogen.^[^ ^76^, ^77^, ^78^ ^]^ A multi‐layer adsorbent bed is used for this process, with the first layer (activated carbon, alumina, or silica) removing moisture and heavy components, the second layer (activated carbon) removing CO_2_ and part of the CO and CH_4_, and the final layer (Zeolite 5A or 13X) removing the final traces of CO, CH_4,_ and N_2_.^[^ ^78^ ^]^

CO_2_ and H_2_ have kinetic diameters (3.3 and 2.89 Å), polarizabilities (26.5 × 10^−25^ and 7.9 × 10^‐25^ cm^3^) and quadrupole moments (4.3 × 10^−26^ and 0.66 ×10^−26^ esu cm^2^) respectively. The polarizability and quadrupole moments suggest that design strategies for CO_2_/H_2_ selective materials could exploit an increased interaction strength between CO_2_ and the material surface, whereas H_2_/CO_2_ selective materials could exploit the difference in kinetic diameter (see Table S1, Supporting Information for a complete comparison of physical parameters).

### 11.1 Breaking the CO_2_/H_2_ Uptake versus Selectivity Upper Bound

The CO_2_/H_2_ selectivity versus uptake plot (Figure 10A) shows a trade‐off between uptake and selectivity, where high selectivity is only achieved at a reduced CO_2_ uptake. Adsorbent materials that break the CO_2_/H_2_ bound are all metal‐site containing MOFs, with the exception of a series of nitrogen‐doped porous carbons synthesized from a mixture of algae and glucose and activated with potassium hydroxide. Noting that the selectivities are IAST derived, two of these carbon materials exceed the bound, NAHA‐1 (5.3,10 000,‐28.5) and NAHA‐2 (6,3500,‐27).^[^ ^79^ ^]^ These adsorbents have large surface areas and nitrogen doping that interacts with the CO_2_ quadrupole moment. The other carbon materials from that work (which experience milder potassium hydroxide activation) sit just inside the bound.

Similar to the carbon‐based materials, the metal‐site MOFs rely on large surface areas to achieve high CO_2_ capacity and differences in interaction strength to obtain high CO_2_/H_2_ selectivity. Examples of these MOFS are Cu‐TDPAT (5.2,142,‐26),^[^ ^5^ ^]^ di‐hydroxyl modified UiO‐66(Zr) (5.6,2854,‐33)^[^ ^80^ ^]^ and Zeolite 5A/Ni‐MOF‐74 composites (7.4,1450,‐13) and (7,175,‐13).^[^ ^38^, ^39^ ^]^ Mg‐MOF‐74 also features at the very high uptake end outside of the bound (8.75,88,‐35)^[^ ^46^ ^]^ due to strong interactions with CO_2_, which are discussed above as an outlier material for the CO_2_/N_2_ and CO_2_/CH_4_ gas pairs. The di‐hydroxyl modified UiO‐66 variants, [UiO‐66(Zr)‐(OH)_2_ (5.6,2854,‐33) and UiO‐66(Hf)‐(OH)_2_ (4,1722,‐30)],^[^ ^80^ ^]^ increase the polarity of the pore walls and hence the CO_2_/H_2_ selectivity and heat of adsorption.

As with earlier gas pairs, the core–shell composite structure of Ni‐MOF‐74 and 5wt% Zeolite 5A surpasses this bound.^[^ ^38^, ^39^ ^]^ The resulting material displays both increased capacity (7.4 vs 5.5 mol kg^−1^) and selectivity (1450 vs 900) over Ni‐MOF‐74 alone. The increase in capacity is attributed to mesopores that form at the MOF‐zeolite interface. The increased selectivity appears to be a result of the interfacial mesopores increasing the CO_2_ capacity, but not the H_2_ capacity (rather than a size‐sieving effect typically targeted by core–shell designs). The heat of adsorption recorded for ZeoA@MOF‐74 is −13 kJ mol^−1^ (1 bar) which is remarkably low; however, the heat of adsorption increases to −32 kJ mol^−1^ at low loading (<3 mol kg^−1^).

An interesting material at the edge of the bound is Cu‐TDPAT (6,240,‐32),^[^ ^81^ ^]^ (5.2,142,‐26).^[^ ^5^ ^]^ Cu‐TDPAT is stable under humid conditions and has a high density of both open metal sites and Lewis basic sites. Furthermore, the authors point out the utility of materials with large pores when they are operating at higher pressures because they can pack more gas into the open volumes. This insight is particularly potent for separations where one component is extremely weakly adsorbing, such as H_2_.

### 11.2 Breaking the CO_2_/H_2_Uptake versus Heat of Adsorption Lower Bound

The CO_2_/H_2_ uptake versus heat of adsorption plot (Figure 10B) shows a clear trade‐off between uptake and selectivity, where high CO_2_ uptake cannot be achieved at low heat of adsorption. The information in this bound describes CO_2_ only, so is identical for the other CO_2_ gas pairs (CO_2_/N_2_ Figure 6B and CO_2_/CH_4_ Figure 9B). Because of this, there are only two outliers for CO_2_/H_2_, so this discussion will also look at the materials closest to the bound.

The two outliers are the core–shell Zeolite 5A/Ni‐MOF‐74 composites Zeo‐A@Ni‐MOF‐74 (7.4,1450,‐13) and Zeo‐B@Ni‐MOF‐74 (7,175,‐12.5) (that also break the uptake versus selectivity bound).^[^ ^38^, ^39^ ^]^ These materials recur in the CO_2_/CH_4_ section, and incorporate 5wt% Zeolite 5A, which increased the capacity compared to Ni‐MOF‐74 alone due to the new mesopores at the MOF‐zeolite interface. The non‐selective pore volume (or adsorption surface area), i.e., the empty mesopore space, allows this material to exceed the bound by averaging out the heat of adsorption and selectivity provided by the Zeolite and Ni‐MOF‐74. The difference between these two composites is that Zeo‐B uses carboxylic‐functionalized Zeolite 5A, while Zeo‐A uses standard Zeolite 5A, which may have influenced the prevalence and nature of the mesopores formed. The recorded heat of adsorption is similar for these two materials, however, at low loading, Zeo‐B peaks at −24 kJ mol^−1^ while Zeo‐A peaks at −32 kJ mol^−1^. Even considering this increased heat of adsorption at low loading, these materials would still be on the bound.

The remaining materials that break this bound are open metal site MOFs, however these materials represent the limitation of our chosen standard conditions, rather than exceptional performance. In these materials, the heat of adsorption is higher at the lower pressures where the majority of gas adsorption occurs.

The other materials that broke the uptake versus selectivity bound are also closest to breaking this uptake versus heat of adsorption bound: NAHA‐x carbons,^[^ ^79^ ^]^ Mg‐MOF‐74,^[^ ^46^ ^]^ Cu‐TDPAT^[^ ^5^, ^81^ ^]^ and UiO‐66(Zr/Hf)‐(OH)_2_.^[^ ^80^ ^]^

### 11.3 Breaking the CO_2_/H_2_ Heat of Adsorption versus Selectivity Lower Bound

The CO_2_/H_2_ heat of adsorption versus selectivity lower bound (Figure 10C) is horizontal, reflecting a minimum CO_2_ heat of adsorption, until a CO_2_/H_2_ selectivity of ca. 20 is reached. Past a CO_2_/CH_4_ selectivity of 20, a clear lower‐bound appears, with several materials that surpass it.

A range of all our material classifications overcome this bound, reflecting that multiple strategies are effective for increasing CO_2_/H_2_ selectivity. The extension of the “flat line” for CO_2_ (CO_2_/H_2_  =  20) is between CO_2_/N_2_ (100) and CO_2_/CH_4_ (10).

Leading adsorbent materials that break the other CO_2_/H_2_ bounds, also perform well against this bound: Zeolite 5A/Ni‐MOF‐74 composites,^[^ ^38^, ^39^ ^]^ NAHA‐x carbons,^[^ ^79^ ^]^ Cu‐TDPAT^[^ ^5^, ^81^ ^]^ and UiO‐66(Zr/Hf)‐(OH)_2_.^[^ ^80^ ^]^ Materials that break this bound that were not present in the uptake versus selectivity or uptake versus heat of adsorption plots are Ni‐MOF‐74 Monolith (2.8360,‐22.4),^[^ ^82^ ^]^ UTSA‐16 (1.2120,‐26.5),^[^ ^83^ ^]^ COF‐IL@chitosan (1,55,‐16)^[^ ^41^ ^]^ and a range of unmodified zeolites.

The Ni‐MOF‐74 monolith^[^ ^82^ ^]^ was produced by 3D printing a mixture of Ni‐MOF‐74 (synthesized from the solvothermal method), bentonite clay, and PVA. This paper does not report the performance of Ni‐MOF‐74 before printing with binders, and there are no other reports of Ni‐MOF‐74 for CO_2_/H_2_ separation for comparison. A conclusion on the effect of the monolith binder mixture on CO_2_/H_2_ separation is therefore not possible.

UTSA‐16 (1.2120,‐26.5)^[^ ^83^ ^]^ sits just inside the bound. It is a MOF comprised of cobalt, potassium, and citrate with a dense zeolite‐like structure.^[^ ^84^ ^]^ Other reports of the material's performance include higher capacity (3.2,100,‐N/A).^[^ ^85^ ^]^ However, another report of UTSA‐16^[^ ^86^ ^]^ (not including CO_2_/H_2_ data) reports a higher heat of adsorption for CO_2_, between −36 and ‐40 kJ mol^−1^, which would place it within the lower bound.

COF‐IL@chitosan (1,55,‐16)^[^ ^41^ ^]^ sits outside the low selectivity end of the sloped bound. It is a composite material combining a covalent‐organic framework (COF) chemically modified with imidazolium ionic liquids and a non‐porous polymer base material, chitosan‐SH (which also happens to be the only material below the horizontal −10 kJ mol^−1^ bound). The composite maintained the high CO_2_ selectivity of the COF‐IL while making the material more robust and easily shaped.

Several un‐modified zeolites also perform well on this bound, sitting outside or just inside the bound. Zeolite 4A (3.6,1700,‐36),^[^ ^87^ ^]^ Zeolite 5A (2.5,250,‐30)^[^ ^88^ ^]^/(4,200,‐35),^[^ ^89^ ^]^ Zeolite 13X (4.9,205,‐33.4)^[^ ^90^ ^]^/(4.7,233,‐33.5)^[^ ^91^ ^]^ and NaY zeolite (4.8,192,‐31).^[^ ^92^ ^]^ This wide range of low‐cost effective adsorbents is reflective of why PSA is used industrially to separate CO_2_/H_2_ mixtures with >70% H_2_. These zeolitic materials all report remarkably similar uptake, selectivity and heat of adsorption. The selectivity of Zeolite 4A is higher because it uses the IAST method, while all others used the uptake ratio method. Applying the uptake ratio method to Zeolite 4A reduces its selectivity to a similar order of magnitude as the other materials with a selectivity of approximately 100.

### 11.4 Leading Designs for Selective CO_2_/H_2_ Adsorption

Overall, the best adsorbents for CO_2_/H_2_ achieve performances inferior to those seen for the CO_2_/N_2_ bound, which is surprising since the properties of H_2_ would infer weaker interactions than N_2_. The leading design strategy relies on enhancing thermodynamic selectivity for CO_2_ and minimizing non‐selective surface area. This is probably reflected in that H_2_ isotherms are not routinely measured like N_2_ and CH_4_. That is surprising in turn, given that industrial CO_2_/H_2_ separation is achieved by PSA and improvements to adsorbent performance could significantly improve the energy efficiency and capital‐cost requirements, in contrast for CO_2_/N_2_ separation which is not yet performed on a large scale.

The testing of materials for H_2_ selectivity is therefore an opportunity for the field, realizing that competition will be with zeolitic materials that achieve relatively good capacity (ca. 6 mol kg^−1^) and selectivities (>100) and are also cheap. New, higher capacity and selectivity adsorbent materials reported for the CO_2_/N_2_ separation could achieve an impact in this industry if their production costs could be reduced to the same order of magnitude as Zeolite materials (ca. <$USD 5 kg^−1^ at the time of publishing).

## 12 Methane—Hydrogen

**Figure 11:**
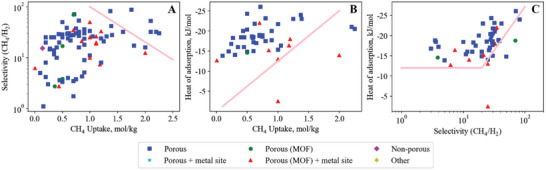
Methane–hydrogen empirical bounds illustrating the trade‐off between A) CH_4_ uptake versus CH_4_/H_2_ selectivity (upper bound), B) CH_4_ uptake versus CH_4_ heat of adsorption (lower bound) and C) CH_4_/H_2_ selectivity versus CH_4_ heat of adsorption (lower bound).

Our search method identified 95 materials studied for adsorption of the CH_4_/H_2_ gas pair. Similar to CO_2_/H_2_ separation, CH_4_/H_2_ separation is also useful in hydrogen production processes where steam methane reforming is used to purify off gases typically composed of H_2_, CO_2_, CH_4_, CO, and N_2_.^[^ ^93^, ^94^ ^]^ Pressure swing adsorption is current standard for this purification.^[^ ^93^, ^94^ ^]^ Methane – hydrogen separation also enables the use of existing natural gas pipeline networks for hydrogen/methane mixtures, without the need for dedicated hydrogen pipelines.^[^ ^95^, ^96^, ^97^ ^]^ The upper and lower bound trade‐offs for methane–hydrogen materials are illustrated in **Figure** **11**.

CH_4_ and H_2_ have kinetic diameters (3.8 and 2.89 Å), polarizabilities (26 × 10^−25^ and 7.9 × 10^−25^ cm^3^) and quadrupole moments (0 and 0.66 ×10^−26^ esu cm^2^) respectively. The minor differences in polarizability and quadrupole moment suggest that design strategies exploiting these for preferential adsorption of CH_4_ could be achieved, as is evidenced in the materials we found. Additionally, the smaller kinetic diameter of hydrogen could achieve inverse H_2_/CH_4_ selectivity. See Table S1, Supporting Information for a complete comparison of physical parameters.

### 12.1 Breaking the CH_4_/H_2_ Uptake versus Selectivity Upper Bound

The CH_4_/H_2_ selectivity versus uptake plot (Figure 11A) shows a trade‐off between uptake and selectivity, where high selectivity is only achieved at a reduced CH_4_ uptake. Only two materials reported CH_4_/H_2_ separations using the IAST method. CH_4_/H_2_ separation was often overlooked, with papers reporting IAST selectivity for other gas pairs but not CH_4_/H_2_, leading to use of the uptake ratio method in our analysis.

The best materials for CH_4_/H_2_ adsorption are all activated carbons, compared to the CO_2_/N_2_, CO_2_/CH_4_ and CO_2_/H_2_ gas pairs which had a range of material classes perform well. The nitrogen‐doped carbons produced from algae and glucose (NAHA‐x)^[^ ^79^ ^]^ that performed well for other separations also performed well for CH_4_/H_2_. Three variants lie outside of the bound: NAHA‐0.5 (2.1,32,‐N/A), NAHA‐1 (2.2,32,‐21), and NAHA‐2 (2.25,30,‐20.5), while one variant sits just inside the bound: NAHA‐4 (1.8,17,‐N/A). Each variant increased the amount of KOH mixed in during chemical activation (from NAHA‐0.5 to NAHA‐4), reducing the nitrogen content. Activated mesocarbon microbeads also sit just inside the bound (2,16,‐N/A).^[^ ^98^ ^]^

Two reports of etched carbon aerogels also performed well. The first was produced by pyrolysis of resorcinol‐formaldehyde (RF) aerogel, followed by CO_2_ etching to give a material labeled EC‐RF (1.75,88,‐N/A).^[^ ^57^ ^]^ The second produced polymeric aerogel by the sol‐gel method using pyromellitic acid (PMA) as the precursor. The polymeric aerogel was carbonized to give carbon aerogel (1.6,32,‐N/A) and then etched with CO_2_ to give etched carbon aerogel (2.1,53,‐N/A).^[^ ^99^ ^]^

Activated carbon modified with urea to increase nitrogen content also passes the bound on the high‐selectivity end. The un‐modified activated carbon sits just inside the bound (1.4,42,‐23), while after modification with a urea/ethanol mixture, the material exceeded the bound (1.4,82,‐24).^[^ ^100^ ^]^ This paper also investigated oxidizing both of these samples with nitric acid to increase the concentration of oxygen functional groups; however, the oxidizing step reduced the capacity, placing the oxidized materials well within the bound (oxidized activated carbon (0.6,30,‐15) and oxidized urea‐modified activated carbon (0.7,71,‐26)).^[^ ^100^ ^]^

No metal‐organic frameworks overcame the uptake—selectivity bound for CH_4_/H_2_ separation. The three MOFs closest to the bound are MOF‐74 variants Ni^[^ ^57^ ^]^ (1,50,‐N/A) and Mg^[^ ^46^ ^]^ (2,12.5,‐14), and Cu‐TDPAT (1.2,33,‐18).^[^ ^5^ ^]^

### 12.2 Breaking the CH_4_/H_2_ Uptake versus Heat of Adsorption Lower Bound

The CH_4_/H_2_ uptake versus heat of adsorption plot (Figure 11B) shows a clear trade‐off between uptake and heat of adsorption, where few materials achieve a high CH_4_ uptake and low heat of adsorption. While the ca. −20 kJ mol^−1^ heat of adsorption interaction strength is similar to that seen for the best CO_2_ selective materials, the CH_4_ uptake is significantly lower than CO_2_ (max. 2 mol kg^−1^ for CH_4_ vs max 8 mol kg^−1^ for CO_2_) and appears related to the difference in quadrupole moments rather than polarizability or size.

The high surface areas of activated carbons, combined with nitrogen doping to promote CH_4_ adsorption, are most successfully embodied in NAHA‐1 (2.2,32,‐21) and NAHA‐2 (2.25,30,‐20.5).^[^ ^79^ ^]^ The heats of adsorption seem high given the relatively low uptake and suggest that the capacity of these materials is limited by the number of adsorption sites (i.e., amount of nitrogen doping), as observed for the materials in Section 10.2, Table 4.

Mg‐MOF‐74 (2,12.5,‐14) also performs well on this bound,^[^ ^46^ ^]^ with core–shell composites of Zeolite 5A/Ni‐MOF‐74, Zeo‐A@Ni‐MOF‐74 (1,25,‐2.5) and Zeo‐B@Ni‐MOF‐74 (1,25,‐13), outperforming the parent material.^[^ ^38^, ^39^ ^]^ Zeo‐A uses Zeolite 5A and Zeo‐B uses a carboxylic‐functionalized Zeolite 5A. As with previous core–shell strategies, the increase in capacity is likely due to the creation of mesopores at the Zeolite/MOF interface, as indicated by the drop in selectivity and increase in capacity compared to the parent materials.

### 12.3 Breaking the CH_4_/H_2_ Selectivity versus Heat of Adsorption Lower Bound

The CH_4_/H_2_ heat of adsorption versus selectivity lower bound (Figure 11C) is horizontal, reflecting a minimum CH_4_ heat of adsorption of −12 kJ mol^−1^ until a CH_4_/H_2_ selectivity of ca. 20 is reached. Past a CH_4_/H_2_ selectivity of 20, a clear bound appears that correlates to a trade‐off of increasing selectivity requiring higher CH_4_ heat of adsorption.

The two core–shell Zeolite 5A/Ni‐MOF‐74 composites^[^ ^38^, ^39^ ^]^ identified above also overcome this bound. Similarly, the activated carbons^[^ ^100^ ^]^ that broke the uptake versus selectivity bound also perform well for selectivity versus heat of adsorption, with the urea modified carbon (1.4,82,–24) overcoming the bound at the high selectivity end and the oxidized carbon variant (0.6,30,–15) at the mid‐selectivity area of the bound.

UTSA‐16 (0.7,70,–19)^[^ ^83^ ^]^ sits as an obvious outlier. This source does not describe the material in detail and doesn't discuss the high selectivity, so it is difficult to elicit what makes this framework superior for CH_4_/H_2_ separation.

Cheap zeolites also overcome this bound: Zeolite 5A (0.5,50,–15)^[^ ^101^ ^]^ and MgX Zeolite 13X (0.3,25,–14).^[^ ^101^ ^]^ The latter is Zeolite 13X (0.27,15,–18)^[^ ^101^ ^]^ modified with magnesium ions exchanged into the structure. In contrast, calcium cations increased the uptake and selectivity at the cost of increased heat of adsorption CaX (0.4, 29, −20).^[^ ^101^ ^]^ Similarly, Zeolite 5A (0.5,50,–15)^[^ ^102^ ^]^ appears well outside of the sloped bound with two other reports of Zeolite 5A just inside the bound (0.75,38,–19)^[^ ^89^ ^]^ and (0.7,28,–19).^[^ ^103^ ^]^

### 12.4 Leading Designs for Selective CH_4_/H_2_ Adsorption

Compared with bounds examined so far, the CH_4_/H_2_ is both mature from an industrial viewpoint, in that low‐cost activated carbons feature past the current bound and are used industrially.

## 13 Methane—Nitrogen

**Figure 12:**
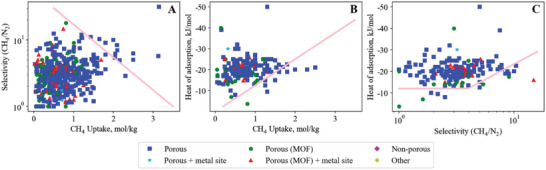
Methane–nitrogen empirical bounds illustrating the trade‐off between A) CH_4_ uptake versus CH_4_/N_2_ selectivity (upper bound), B) CH_4_ uptake versus CH_4_ heat of adsorption (lower bound) and C) CH_4_/N_2_ selectivity versus CH_4_ heat of adsorption (lower bound).

**Figure 13:**
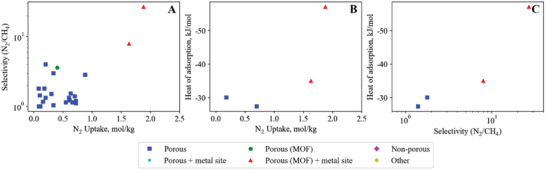
Nitrogen–methane empirical bounds illustrating the trade‐off between A) N_2_ uptake versus N_2_/CH_4_ selectivity, B) N_2_ uptake versus N_2_ heat of adsorption and C) N_2_/CH_4_ selectivity versus N_2_ heat of adsorption. Bound curves have not been fitted because of the lack of materials.

Our search method identified 546 materials studied for adsorption of the CH_4_/N_2_ gas pair, 23 of these materials were nitrogen selective, with the remainder selective for methane. The separation of CH_4_ and N_2_ is important for purifying natural gas to meet pipeline specifications (<3% N_2_)^[^ ^45^ ^]^ and can enable the use of low‐quality natural gas reserves as methane fuel sources. For application of adsorbent materials to natural gas purification via a PSA system, the adsorbents would preferably be nitrogen–selective in order to produce methane as the high‐pressure product.^[^ ^104^ ^]^ However, the majority of reported adsorbent materials are methane selective, as expected from the physical properties of each gas and illustrated in the upper and lower bound trade‐offs for methane selective materials (**Figure** **12**) and nitrogen selective materials (**Figure** **13**).

Methane and nitrogen have kinetic diameters (3.8 and 3.64 Å), polarizabilities (26 × 10^−25^ and 17.6 × 10^−25^ cm^3^) and quadrupole moments (0 and 1.52 × 10^−26^ esu cm^2^) respectively. This suggests methane selectivity can be achieved by leveraging its increased polarizability, while exploiting the greater quadrupole moment or smaller kinetic diameter of nitrogen could yield nitrogen selectivity. In contrast, the triple bond of N_2_ provides *π* electrons that can be targeted by interaction with transition metal ions. See Table S1 (Supporting Information) for a complete comparison of physical parameters.

### 13.1 Leading Designs for Selective CH_4_/N_2_ Adsorption

The three bounds of CH_4_/N_2_ selective adsorbents (Figure 12) show the expected trade‐offs and have been condensed into a single section to reflect their lack of industrial relevance and the absence of specific design strategies specific to encouraging methane adsorption over nitrogen. As expected from the physical properties of the gases, the achieved uptakes (<3.2 mol kg^−1^) and selectivities (<12, with one outlier at 31) are relatively low compared to other gas pairs.

Activated carbon materials produced via carbonization of natural products^[^ ^40^, ^105^, ^106^, ^107^ ^]^ and microporous polymers^[^ ^108^, ^109^, ^110^, ^111^ ^]^ account for the majority of outliers on all 3 bounds. Performance can be correlated to the prevalence of ultramicropore volume, to promote preferential adsorption of the larger and more polarizable methane,^[^ ^106^ ^]^ and prevalence of nitrogen doping, to increase the number of methane adsorption sites.^[^ ^112^ ^]^ These strategies are similarly prevalent in zeolite,^[^ ^113^ ^]^ MOF,^[^ ^114^, ^115^, ^116^ ^]^ and MOF/ionic liquid composites^[^ ^117^ ^]^ which exceed the bounds.

### 13.2 Leading Designs for Selective N_2_/CH_4_ Adsorption

The properties of nitrogen‐selective adsorbents are shown in Figure 13. While we did not identify enough materials to assign current bounds, the best design strategies for producing N_2_ selective adsorbents are obvious from the bound plots.

N_2_/CH_4_ selectivity >10 has been achieved by chemisorption of nitrogen through back‐bonding interactions between the unoccupied N_2_ π* orbital and open metal sites.^[^ ^118^, ^119^ ^]^ The vanadium(II) ion has shown most promise in V_2_Cl_2.8_(btdd) (1.9, 27, −57).^[^ ^119^ ^]^ In addition, the MIL‐101(Cr) MOF (1.6, 8, −35)^[^ ^118^ ^]^ also showed reasonable selectivity. As expected for a separation reliant on chemisorption, the higher N_2_ capacity and selectivity are concomitant with much higher heats of adsorption.

The N_2_/CH_4_ gas pair also highlights a limitation of the bound analysis, where performance is only assessed at equilibrium. Kinetic N_2_/CH_4_ selectivity of >10 has been observed for strontium‐exchanged zeolite ETS‐4 variants,^[^ ^120^ ^]^ ion exchanged clinoptilolites (for example, the methane selective Sr‐Clinoptilolite (1.3, 1.2, N/A) or the nitrogen selective Ce‐Clinoptilolite (0.9, 2.8, N/A),^[^ ^121^ ^]^ and MSC‐3K 172 (methane selective at equilibrium) (0.8, 2.4, −19).^[^ ^122^ ^]^ In these materials, nitrogen diffusivities are 1–2 orders of magnitude greater than for methane. In the example of strontium‐exchanged zeolite ETS‐4,^[^ ^120^ ^]^ the kinetic selectivity produces a scenario where, despite higher methane capacity at equilibrium, the faster nitrogen adsorption rate means that when nitrogen reaches equilibrium, methane is only at 20% of its equilibrium loading.

## 14 Oxygen—Nitrogen

Our search method identified 284 materials studied for adsorption of the O_2_/N_2_ gas pair (80 O_2_‐selective materials and 204 N_2_‐selective materials). O_2_/N_2_ separation is important for the production of pure oxygen and nitrogen from air, where cryogenic distillation^[^ ^123^ ^]^ (large scale) or pressure swing adsorption^[^ ^123^, ^124^, ^125^ ^]^ (medium/small scale) is typically used.

Oxygen and nitrogen have kinetic diameters (3.46 and 3.64 Å), polarizabilities (13 × 10^−25^ and 17.6 × 10^−25^ cm^3^) and quadrupole moments (0.4 × 10^−26^ and 1.52 × 10^−26^ esu cm^2^) respectively, while oxygen also has extra valence electrons compared to nitrogen. These physical parameters suggest that for nitrogen‐selective adsorption, exploiting the increased polarizability and quadrupole moment of nitrogen is key. While for oxygen selective adsorption, the extra valence electrons and smaller kinetic diameter could be exploited.

Existing pressure swing adsorption processes typically rely on nitrogen‐selective adsorbents^[^ ^123^, ^125^ ^]^ that exploit the larger quadrupole moment of nitrogen.^[^ ^126^ ^]^ However, when searching for the O_2_/N_2_ gas pair, there were also many oxygen‐selective adsorbents with considerably higher selectivities. With a nitrogen‐selective adsorbent, the immediate product gas will be oxygen‐enriched. In contrast, an oxygen‐selective adsorbent will produce an oxygen‐enriched phase by capture and repressurization of the desorbed gas phase.

### 14.1 Nitrogen‐Selective Materials

**Figure 14:**
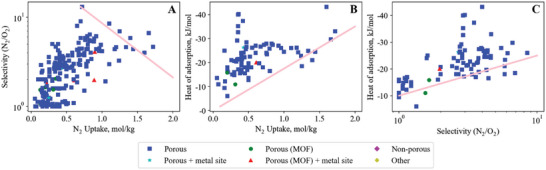
Nitrogen–oxygen empirical bounds illustrating the trade‐off between A) N_2_ uptake versus N_2_/O_2_ selectivity (upper bound), B) N_2_ uptake versus N_2_ heat of adsorption (lower bound) and C) N_2_/O_2_ selectivity versus N_2_ heat of adsorption (lower bound).

Nitrogen‐selective adsorbents had the lowest capacities and lowest selectivities of any gas pair studied (**Figure** **14**). This is related to the lower polarizability and larger size of nitrogen compared to oxygen, leaving neither of the common adsorbent design strategies (increasing interaction strength of adsorption sites or matching cavity size to gas size), applicable for this separation.

The bound plots reveal a rare case of the same adsorbent materials breaking all three N_2_/O_2_ bounds. These adsorbent materials are exchanged low silica type X (LSX) Zeolites, specifically Li‐exchanged variants which are used industrially (1.3,6.9,‐18.5),^[^ ^127^ ^]^ (1.1,8.5,‐26),^[^ ^128^ ^]^(1.4,5.8,‐21)^[^ ^129^ ^]^ and (0.8,4.4,‐17).^[^ ^130^ ^]^ Silver, calcium, and strontium‐exchanged LSX zeolites also perform well: Ag_3.0_Ca_46.5_‐LSX (1.7,6.6,‐30), Ag_2.0_Ca_47_‐LSX (1.7,4.6,‐33) and Ag_3.0_Sr_46.5_‐LSX (1.4,4.4,‐25).^[^ ^129^ ^]^

Molecular simulations suggest the reason for this exceptional performance is due to the higher quadrupole moment of nitrogen versus oxygen and the polarizability of the alkali cations promoting interactions at strengths on par with van der Waals interactions.^[^ ^126^ ^]^ Larger cations are capable of interacting with valence oxygen electrons, leading to the higher selectivities we observed in the O_2_/N_2_ selective materials discussed below.

It is worth noting that N_2_/O_2_ separation is a rare example of an adsorption process being industrially relevant for bulk separations (i.e., production of oxygen and/or nitrogen from air). For materials scientists designing adsorbents, this field is a clear opportunity—that an enterprising, cost‐competitive strategy to increase the capacity of N_2_/O_2_ selective adsorbents would be valuable to this already established industry.

### 14.2 Breaking the O_2_/N_2_ Bounds

**Figure 15:**
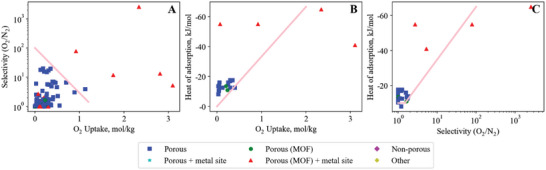
Oxygen–nitrogen empirical bounds illustrating the trade‐off between A) O_2_ uptake versus O_2_/N_2_ selectivity (upper bound), B) O_2_ uptake versus O_2_ heat of adsorption (lower bound) and C) O_2_/N_2_ selectivity versus O_2_ heat of adsorption (lower bound).

After nitrogen, oxygen capacity was the lowest for all the gas pairs studied in this review (**Figure** **15**A). There are two distinct regions in this plot, with the bound limited to porous materials and capacities similar to nitrogen (< = 1 mol kg^−1^) and MOFs with open metal sites at 1–3 mol kg^−1^ that are significantly beyond the porous (non‐MOF and non‐metal‐site) adsorbents’ upper bound. The porous materials forming the current capacity versus selectivity bound rely on physisorption and weak interactions, with no stand‐out design strategies or insights.

The five MOFs outside the bounds use Cr^II^, Fe^II^, and Co^II^ open metal sites for the chemisorption of oxygen. These MOFs are Cr‐BTT (2.3,2570,‐65),^[^ ^131^ ^]^ Fe_2_(dobdc) (3.1,5.3,‐41),^[^ ^132^ ^]^ Cr_3_(BTC)_2_ (2.8,13.4,‐N/A),^[^ ^133^ ^]^ Cr^t^Bu‐bdc (1.8,11.7,‐N/A)^[^ ^134^ ^]^ and [{Co(II)_2_(bpbp)}_2_bdc](PF_6_)_4_ (0.9,78,‐55).^[^ ^135^ ^]^ The consequences of this strategy are as expected: the isotherm shapes are steep at low pressure and high O_2_ capacity and O_2_/N_2_ selectivity are accompanied by high heats of adsorption. While the selectivity of Cr‐BTT seems exceptional, this is because it is the only material to report selectivity using the IAST method.

The O_2_ capacity and O_2_/N_2_ selectivity versus heat of adsorption plots (Figure 15B,C) suffer from a lack of reported heat of adsorption data. However, a key design point is illustrated in that the MOFs relying on the open metal site strategy are still limited by the capacities they can achieve. The absence of alternate adsorption sites for O_2_ and the space required for constructing the open metal sites take a large proportion of the framework space.

### 14.3 Leading Designs for selective O_2_/N_2_ and N_2_/O_2_ Adsorption

While the O_2_/N_2_ selective adsorbents display an order of magnitude higher selectivity than N_2_/O_2_ selective adsorbents, it is N_2_/O_2_ selective adsorbents that find application in the bulk separation of air. This is an important point for materials scientists designing adsorbent materials—in rapid‐cycle PSA for oxygen production the key properties of these successful adsorbents are a kinetic rather than equilibrium selectivity, low cost, and low heat of adsorption. Beating the upper or lower bound will not always be an advance toward practical applications (as discussed in Section 17).

## 15 Argon, Krypton, and Xenon

**Figure 16:**
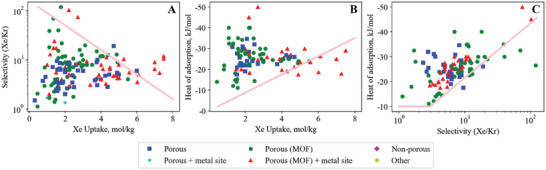
Xenon–krypton empirical bounds illustrating the trade‐off between A) Xe uptake versus Xe/Kr selectivity (upper bound), B) Xe uptake versus Xe heat of adsorption (lower bound) and C) Xe/Kr selectivity versus Xe heat of adsorption (lower bound).

Argon, krypton, and xenon were considered in this review for their separation from each other and from air. The noble gases are used for inert atmospheres (argon),^[^ ^124^ ^]^ lighting (Krypton/Xenon), aeronautics (Xenon), and would be used widely as anesthetics if the price were considerably cheaper (Xenon).^[^ ^136^ ^]^ Adsorption processes usually perform well when removing trace components, noting that Argon (0.93 vol%), Krytpon (1.14 vol%), and Xenon (0.086 vol%) are trace components in the atmosphere, and it is surprising that more research effort has not gone into isolating these valuable gases from air. This is reflected in the absence of enough data to identify clear bounds for the Kr/Ar, Xe/O_2_/N_2_, Kr/O_2_/N_2_ and Ar/O_2_/N_2_ gas pairs (Section S3, Supporting Information). However, the Xe/Kr pair did have enough data points (142 materials) to draw preliminary conclusions (**Figure** **16**).

Krypton (80 vol%) and Xenon (20 vol%) mixtures are typically produced as by‐products of cryogenic distillation of air, which is energy and capital‐intensive making the gases expensive. Adsorption provides an alternative to these processes that could operate at smaller scales with reduced engineering requirements and energy consumption.^[^ ^137^ ^]^ Indeed, hybrid adsorption/membrane systems are used for monitoring nuclear weapons testing via the ^133^Xe isotope.^[^ ^138^ ^]^

### 15.1 Breaking the Xe/Kr Uptake versus Selectivity Upper Bound

The Xe/Kr selectivity versus uptake plot (Figure 16A) interestingly uses Xe/Kr selectivity, which immediately reveals that adsorbent materials studied so far rely on the strength of Xe versus Kr interactions (polarizability 4.04 × 10^−25^ and 2.48 × 10^−25^ cm^−3^, respectively) instead of size selectivity (kinetic diameter 4.1 and 3.6 Å respectively).^[^ ^137^ ^]^ Our search only identified one material that was Kr/Xe selective, similar to the CH_4_/H_2_ gas pair, where a significant gas size difference was not exploited for size‐sieving under equilibrium conditions.

To increase the interaction strength with Xenon, multiple adsorbents use the strategy of adding open metal sites. For example, X‐MOF‐74 variants (X = Co (7.5,12,‐29),^[^ ^139^ ^]^ Co (6.1,5,‐26),^[^ ^140^ ^]^ Mg (7,6.5.‐25),^[^ ^139^ ^]^ Zn (4.5,6.5,‐27)^[^ ^139^ ^]^) and PCN‐14 (7.3,5.4,‐18)^[^ ^140^ ^]^ achieve selectivities greater than 5 and the highest capacities of all adsorbents recorded for Xe/Kr. Similarly, the framework Ag‐MOF‐303 (5.3,10.4,‐30)^[^ ^141^ ^]^ contains an unsaturated silver(I) site within the pores. Alternatively, GBC‐900 (4.7,19,‐26)^[^ ^142^ ^]^ uses oxygen‐functionalities (—OH and —COOH) instead of metal sites to interact with the increased polarizability of Xenon.

The design theme of matching cavity size to Xenon also exceeds the bound. The Zeolite‐templated carbon adsorbent beta‐ZTC (6,6,N/A)^[^ ^143^ ^]^ and similar Z11CBF‐1000‐2 (4.9,13,‐23),^[^ ^144^ ^]^ a carbonized ZIF11, likely fall into the cavity‐size matching category – with the authors of both papers reporting a range of narrow pore sizes in the adsorbent.

Three materials combine the strategies of open metal sites and cavity size matching to achieve the highest Xe/Kr selectivities reported:, ZJU‐74a‐Ni (2.7,75,‐50)^[^ ^145^ ^]^ and ZJU‐74‐Pd (2.2,103,‐45).^[^ ^145^ ^]^ As expected, a higher heat of adsorption is observed for both materials.

Mn(ina)_2_ (1.8,120,‐27)^[^ ^146^ ^]^ also lies above the upper bound. Mn(ina)_2_ is a flexible MOF that shows Xenon selectivity above 260K and Krypton selectivity below 260 K. The framework flexibility results in a gate‐opening mechanism and step isotherm shape. At temperatures above 260 K, the gate‐opening pressure is lower for xenon than krypton giving excellent Xe/Kr selectivity. However, below 260 K, the gate‐opening pressure is reduced for both gases and a partial gate‐opening allows for size‐sieving of the smaller Krypton to achieve Kr/Xe selectivity.

Overall, these experimental results match the proposed upper limits of Xe/Kr selective materials as identified from a classical density functional theory study that screened both real and hypothetical adsorbent materials.^[^ ^147^ ^]^

### 15.2 Breaking the Xe/Kr Uptake versus Heat of Adsorption Lower Bound

Interestingly, while the pore size‐matching strategy is effective in reducing heat of adsorption for other gas pairs, it does not out‐perform the open metal site strategy in the case of Xenon (Figure 16B). The X‐MOF‐74 variants mentioned earlier as exemplars of the open metal site strategy are all present outside of the lower bound due to their high capacity (X = Co (7.5,12,‐29),^[^ ^139^ ^]^ Co (6.1,5,‐26),^[^ ^140^ ^]^ Mg (7,6.5.‐25),^[^ ^139^ ^]^ Mg (5.6,5.6,‐24),^[^ ^140^ ^]^ Zn (4.5,6.5,‐27).^[^ ^139^ ^]^). Other metal‐site containing MOFs also overcome the lower bound: PCN‐14 (7.3,5.4,‐18), NOTT‐100 (6,3.6,‐19) and HKUST‐1 (4.9,4.2,‐18).^[^ ^140^ ^]^

The only material outside of this lower bound without open metal sites implemented the pore cavity size matching technique: Ni‐MOF (5.5,4.1,‐24).^[^ ^148^ ^]^

### 15.3 Breaking the Xe/Kr Selectivity versus Heat of Adsorption Lower Bound

The Xe/Kr selectivity versus heat of adsorption lower bound (Figure 16C) is horizontal at −10 kJ mol^−1^, similar to other gases, until a Xe/Kr selectivity of ca. 3. Past a Xe/Kr selectivity of 3, a clear bound appears that correlates to a trade‐off of increasing selectivity requiring higher heat of adsorption. Pore size‐matched materials dominate outside of this lower bound.

The X‐MOF‐74 variants that performed well on the uptake bounds are all within the selectivity versus heat of adsorption bound (X = Co (7.5,12,‐29),^[^ ^139^ ^]^ Co (6.1,5,‐26),^[^ ^140^ ^]^ Mg (7,6.5.‐25),^[^ ^139^ ^]^ Mg (5.6,5.6,‐24),^[^ ^140^ ^]^ Zn (4.5,6.5,‐27).^[^ ^139^ ^]^) This indicates that these materials’ reliance on open metal sites to interact with xenon introduces the expected tradeoff of increased heat of adsorption penalty. In fact, all the metal‐site MOFs recorded for Xe/Kr separation are within the selectivity versus heat of adsorption lower bound, suggesting that pore size control is a more effective method than open metal sites for balancing this tradeoff.

The materials that break this bound rely on pore size/cavity control to influence the Van der Waals forces and leverage the increased polarizability of Xenon compared to Krypton to achieve Xe/Kr separation. Some of these materials also broke the capacity versus selectivity upper bound: Z11CBF‐1000‐2 (4.9,13,‐23),^[^ ^144^ ^]^ GBC‐900 (4.7,19,‐26)^[^ ^142^ ^]^ and Mn(ina)_2_ (1.8120,‐27),^[^ ^146^ ^]^ while the other outliers only broke the selectivity versus heat of adsorption bound: Squarate 1a (1.3,70,‐33),^[^ ^149^ ^]^ Ni(4‐DPDS)_2_CrO_4_ (1.6,40,‐30),^[^ ^150^ ^]^ CROFOUR‐1‐Ni (1.8,22,‐29),^[^ ^151^ ^]^ Zr‐Fum‐Me (1.9,11.8,‐23)^[^ ^152^ ^]^ and Noria (1.6,9.5,‐18).^[^ ^153^ ^]^ In addition to pore cavity size control, GBC‐900, Squarate 1a, Ni(4‐DPDS)_2_CrO_4_ and CROFOUR‐1‐Ni also utilize on framework functionalities that increased the polarity of pore walls and the interaction strength with Xenon. The flexible framework Mn(ina)_2_ (1.8120,‐27)^[^ ^146^ ^]^ discussed as an outlier in the capacity versus selectivity upper bound also breaks the selectivity versus heat of adsorption bound.

Perhaps surprisingly for a noble gas, Xenon is capable of inducing structural changes similar to those used to reduce the overall heat of adsorption. The example we found was a xenon‐induced phase change in framework SIFSIX‐3‐Ni (2.5,4.5,‐15),^[^ ^154^ ^]^ which has a linear krypton isotherm but a unique xenon isotherm shape with an inflection point at ca. 45 kPa. The inflection point is attributed to a comparatively subtle structural change, where pyrazine rings organize their rotational configurations as xenon loading increases.

### 15.4 Leading Materials for Selective Xe/Kr Adsorption

Both pore size matching and open metal sites were found to be leading strategies for Xe/Kr adsorbents. However, the pore size‐matching strategy promises better performance on the heat of adsorption versus selectivity bound; although both strategies will be hampered by the capacity trade‐off required to promote stronger interactions between the frameworks and xenon atoms.

## 16 Reproducibility

Process engineers require reliable and reproducible adsorption data for use in modeling and design. Likewise, materials scientists require reliable data to create accurate descriptions of structure‐function relationships and optimize materials design. Understanding the reliability and reproducibility of data is therefore crucial to optimizing use of material resources and time.

The reproducibility of results is an important consideration for the identification of leading materials in this review. For example, AlOF^[^ ^43^ ^]^ is a promising material identified in the CO_2_/N_2_ uptake versus selectivity bound; however, there are no other reports of this material. Answering the question of how far this single report and others like it can be trusted poses a challenge and has been investigated and commented on by Sholl et al.^[^ ^155^ ^]^ and Nguyen et al.,^[^ ^156^ ^]^ among others.

Our collected data set can add evidence to previous investigations. The materials with most numerous reports chosen for this comparison are Zeolite 13X (NaX zeolite) (41 reports), HKUST‐1 (Cu‐BTC) (23 reports), UiO‐66 (13 reports) and ZIF‐8 (16 reports). This selection covers a range of material types (Zeolite, MOF, and MOF + metal site). Isotherms for the most common gases (CO_2_, CH_4_ and N_2_) are compared to observe and account for the differences in the reported literature.

For each report of these materials identified, the isotherm closest to 293K was digitized and the activation temperature/duration was recorded. In the case of an unclear temperature or duration, no value was recorded. For example, “Activated at 473K until no further mass loss was observed” would not have a duration recorded. Similarly, “Activated under vacuum for 4 h at the appropriate temperature for each material” would not have a temperature recorded, unless the appropriate temperatures were defined within the manuscript.

**Figure 17:**
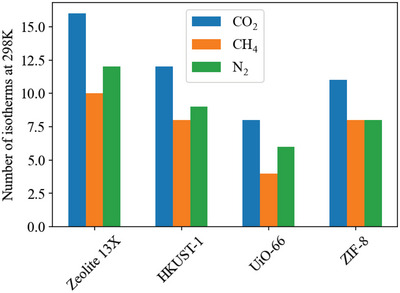
Number of 298 K isotherms for each material/gas combination identified in this literature search.

298K was chosen as the comparison temperature because it was the most common isotherm temperature for the chosen materials. **Figure** **17** shows the number of isotherms for each material/gas combination.

### 16.1 Zeolite 13X, HKUST‐1, UiO‐66 and ZIF‐8

**Figure 18:**
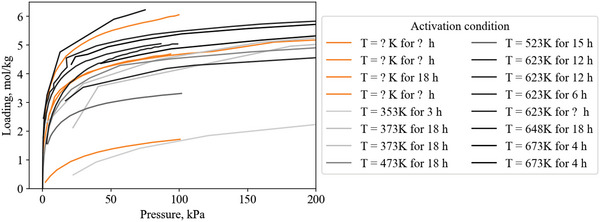
Zeolite 13X CO_2_ 298 K isotherm reproducibility. Darker gradient indicates higher activation temperature, with orange indicating an unknown activation temperature. “?” indicates an unknown activation temperature or duration.

**Figure 19:**
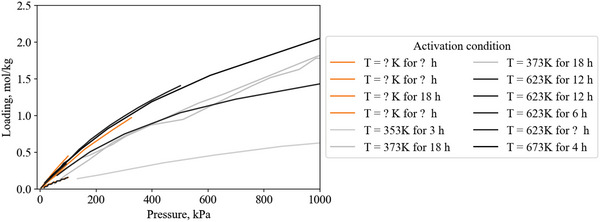
Zeolite 13X N_2_ 298 K isotherm reproducibility. Darker gradient indicates higher activation temperature, with orange indicating an unknown activation temperature. “?” indicates an unknown activation temperature or duration.

**Figure 20:**
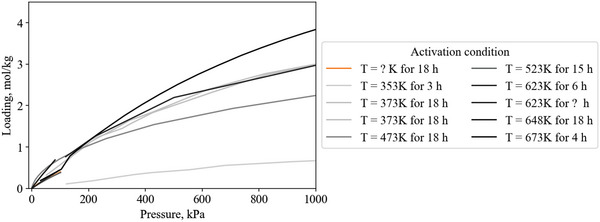
Zeolite 13X CH_4_ 298 K isotherm reproducibility. Darker gradient indicates higher activation temperature, with orange indicating an unknown activation temperature. “?” indicates an unknown activation temperature or duration.

The reproducibility of CO_2_ (**Figure** **18**), N_2_ (**Figure** **19**), and CH_4_ (**Figure** **20**) isotherms on Zeolite 13X were selected for discussion. All other materials can be found in Section S5 (Supporting Information). In Figures 18, 19, 20, a grayscale gradient is used to visualize the activation temperature, with darker lines indicating a higher activation temperature and orange indicating an unknown activation temperature. Similar results were observed for HKUST‐1 (Cu‐BTC/Basolite‐C300), UiO‐66, and ZIF‐8 (Section S5, Supporting Information).

The variance in Zeolite 13X isotherms follows a rough trend where higher activation temperature results in higher gas loading. The low temperature (<373 K) results can be explained by considering that the equilibrium condition for a completely evacuated structure is not achieved, or the kinetics are too slow to complete evacuation of the residual moisture/solvent, and the pores are still occupied to some degree.

**Figure 21:**
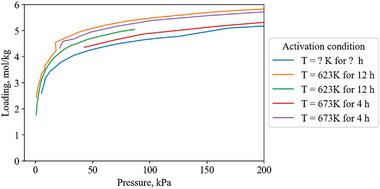
CO_2_ isotherms at 298 K on Zeolite 13X purchased from Zeochem.^[^ ^90^, ^158^, ^159^, ^160^, ^161^ ^]^ The narrow range of activation conditions suggests that isolating synthesis/source alone is not sufficient to remove variability.

Similarly, there is variance with isotherms measured after activation at >600 K. This variance cannot be explained as easily and is more likely due to variation in the material itself. In the reports we found, every 298K CO_2_ isotherm on Zeolite 13X was performed on a purchased/provided sample, with the exception of one case that synthesized it themselves.^[^ ^157^ ^]^ Zeolite 13X was purchased from Zeochem,^[^ ^90^, ^158^, ^159^, ^160^, ^161^ ^]^ UOP,^[^ ^52^ ^]^ CWK,^[^ ^162^ ^]^ Zeo‐Tech,^[^ ^163^ ^]^ Luoyang Jianlong Micro‐Nano New Materials,^[^ ^158^ ^]^ Sigma‐Aldrich,^[^ ^164^ ^]^ Klostrolith,^[^ ^165^ ^]^ SINOPEC^[^ ^47^ ^]^ and CECA.^[^ ^166^ ^]^ Zeochem was the only common supplier, with five reports. The Zeolite 13X isotherms on Zeochem‐supplied samples are compared in **Figure** **21** below to minimize the effect of synthesis as a variable. The activation conditions were fairly consistent between reports (>600 K), but the results vary by up to 1 mol kg^−1^ at 100 kPa. This suggests either significant variation in the material from a single supplier, that activation conditions have a random effect, or that there is significant variation in measurement accuracy/technique.

### 16.2 Sources of Variance

Synthetic variation (e.g., solvent choice, temperature, reaction time), activation conditions (e.g., moisture and solvent retention, oxidation) and the method of isotherm measurement are possible sources for variance in isotherm equilibrium loading. These three possibilities are explored through other literature work where reference isotherms are developed^[^ ^156^, ^167^ ^]^ and the same material is synthesized under different conditions.^[^ ^168^, ^169^ ^]^

#### 16.2.1 Isotherm Measurement

Variance from isotherm measurements can be eliminated after considering the work of Ngyuen et al. Those authors applied the concept of a reference isotherm for validating high pressure adsorption equipment and examined CO_2_ on ZSM‐5 and CH_4_ on Zeolite Y.^[^ ^156^, ^167^ ^]^ Using NIST reference material RM 8852 (ammonium ZSM‐5), eleven labs produced a CO_2_ isotherm at 293.15K up to 4.5 MPa and using NIST reference material RM 8850 (Zeolite Y), twenty labs produced a CH_4_ isotherm at 293.15K up to 7.5 MPa.

**Figure 22:**
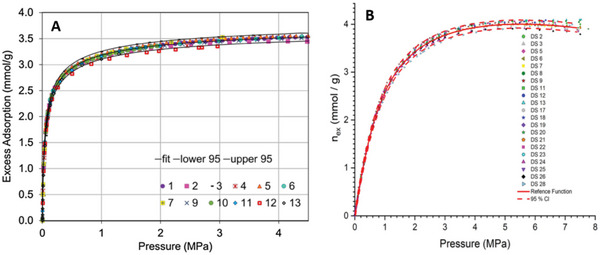
A) Reference isotherm experiments for CO_2_ on ZSM‐5, with a 95% confidence interval of 0.075 mol kg^−1^. Reproduced with permission under Creative Commons license CC BY 4.0.^[^ ^156^ ^]^ Copyright 2018, Springer. B) Reference isotherm experiments for CH_4_ on Zeolite Y, with a 95% confidence interval of 0.09 mol kg^−1^. Reproduced with permission under Creative Commons license CC BY 4.0.^[^ ^167^ ^]^ Copyright 2020, Springer.

To eliminate variance in synthesis conditions, all labs were provided with a portion of the same material sample. Likewise, variation in activation conditions was eliminated by using a common activation condition. The result was a highly repeatable series of statistically repeatable isotherm measurements (**Figure** **22**). This work effectively rules out variance in isotherm measurement as a serious source of error.

#### 16.2.2 Activation Conditions

**Figure 23:**
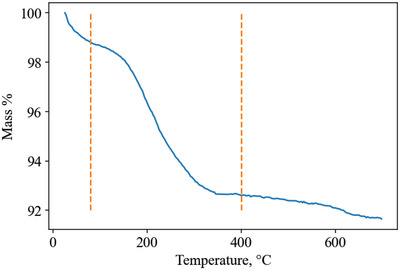
Zeolite 13X (bought from Sigma‐Aldrich) TGA curve at a ramp rate of 10 °C min^‐1^, showing a significant mass loss between 80 °C and 400 °C.

Activation conditions contribute to the observed isotherm variance. The activation conditions should have a predictable effect on adsorption capacity, where insufficient activation reduces capacity due to retention of solvents/previous guests and excessive activation causes degradation of the material. Thermogravimetric analysis (TGA) replicating the activation condition is an excellent tool to prove the degree of activation and thermal stability of the adsorbent. Furthermore, this can identify and characterize variability between batches of adsorbents. An example TGA for Zeolite 13X, (**Figure** **23**) shows a significant mass loss between 80 °C and 400 °C and accounts for the trend of increasing isotherm capacity with increasing activation temperature (Figures 18, 19, 20). Where TGA is unavailable, the publishing authors could replicate the isotherm measurement after sequential activations at the same condition, as is commonly reported for cycling experiments.

#### 16.2.3 Synthetic Variation

Other studies show that variations in synthesis methods between research groups, individual researchers, and commercial sources are the most likely and significant cause of variance in isotherm measurements. Those studies explored, for example, the effect of HKUST‐1 synthesis conditions on adsorption performance.^[^ ^168^, ^169^ ^]^ The first study compared high‐temperature and low‐temperature synthesis methods,^[^ ^168^ ^]^ and the second compared reaction duration and solvent choice.^[^ ^169^ ^]^ The resulting HKUST samples varied in BET surface area between 801 and 1615 m^2^ g^−1^ and CO_2_ loading at 1 bar/300 K varied from 5.58 to 8.08 mol kg^−1^. At the least, where isotherm data is a key feature of a publication, a key isotherm measurement should be repeated on 3 batches of the material to demonstrate the reproducibility of the synthesis conditions used.

## 17 Additional Adsorbent Properties to Consider

As discussed in our previous review,^[^ ^12^ ^]^ optimizing only the capacity, selectivity and heat of adsorption of an adsorbent may not always be the solution to the engineering problem being targeted. Other adsorbent properties such as kinetics, chemical and thermal stability, and cost are as (or even more) vital to practical applications. All of which will have different optima based on the feed conditions of the gas mixture of interest (composition, temperature, pressure), the surrounding plant environment (heat integration), and the required product purity and recovery.

**Figure 24:**
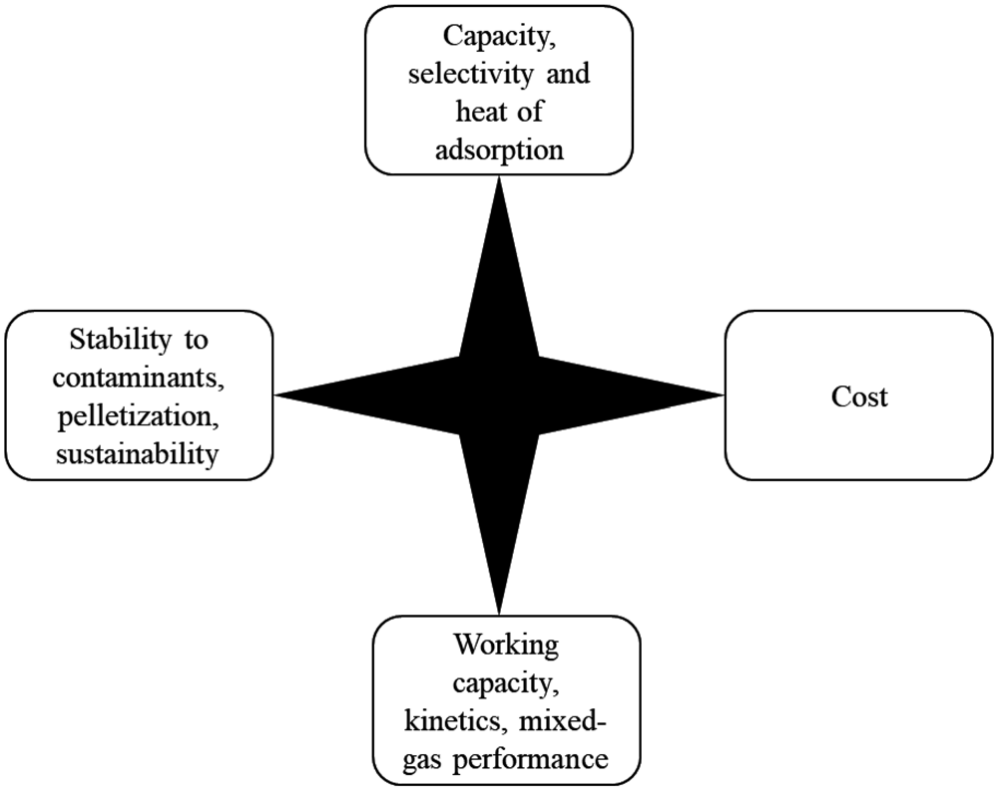
Adsorbent criteria star diagram, illustrating the major considerations for designing and selecting an appropriate adsorbent material.

Other factors include the ability to shape adsorbents (e.g., pelletize) for use on a large scale.^[^ ^78^ ^]^ Sustainable production and carbon footprint of materials is an area of increasing interest, especially given that these adsorbent materials are often developed with the aim of increasing energy efficiency or CO_2_ capture to slow climate change. **Figure** **24** highlights the criteria to consider when designing or selecting an adsorbent for a gas separation. The sections that follow expand with specific examples of how cost, stability and sustainability impact the choice of adsorbent material for an industrial process.

### 17.1 Cost

For bulk separations, where millions of kilograms of adsorbent are required, cost will be the most important factor in choosing an adsorbent material. More correctly, there will be a cost threshold, beyond which it is unreasonable to consider an adsorbent—no matter its properties.

As an example, a representative process modeling work^[^ ^170^ ^]^ (noting that any given work limited by its assumptions and methodology) defined an adsorbent material attractive for CO_2_ capture when it possesses a working capacity >2 mol kg^−1^, CO_2_/N_2_ selectivity >100, and cost <$USD 10 kg^−1^. To illustrate the variance in process modeling conclusions, another economic sensitivity analysis showed that the material cost becomes significant when greater than $USD 6 kg^−1^.^[^ ^2^ ^]^

To illustrate the gains in materials properties required to offset costs, we compare HKUST‐1 against Zeolite 13X using the example VSA process of Ho et al.^[^ ^171^ ^]^ for the capture of 85% of CO_2_ from coal powerplant flue gas. The process required 43 tons (630 m^3^) of Zeolite 13X (2.2 mol kg^−1^; CO_2_/N_2_ 54, ?? kJ mol^−1^) at a cost of $USD 3.2 million using their inflation‐adjusted estimate, or $USD 0.7 million if using suppliers from Alibaba ($USD 1.5 kg^−1^).

Back of the envelope calculations (**Table** **5**) reflect that for bulk gas separations, common adsorbents such as natural and synthetic zeolites have a cost advantage that would require order‐of‐magnitude improvements in the cost and capacity of advanced synthetic adsorbents such as MOFs.^[^ ^78^ ^]^ However, these advanced synthetic adsorbents could find opportunities in smaller‐scale separations and niche applications with high‐value products.

**Table 5:** Cost comparison of Zeolite 13X and HKUST‐1 using three different methods assuming 4x working capacity for HKUST‐1 compared to Zeolite 13X. See Section S6 (Supporting Information) for method details

Total cost in millions of USD	Zeolite 13X	HKUST‐1
Purchase from Sigma‐Aldrich (as of 05/07/2022)	54	3130
Purchasing raw materials from Sigma‐Aldrich (as of 05/07/2022)	32	283
Purchase from adsorbent manufacturer (Alibaba vs NovoMOF + MOF Technologies)	0.7 (as of 05/07/2022)	405^[^ ^172^ ^]^ as of 01/05/2019 851^[^ ^173^ ^]^ as of 13/07/2022

### 17.2 Thermal and Chemical Stability

The thermal and chemical stability of adsorbent materials directly relates to their capital cost and also dramatically affects the risk profile of a gas separation operation.

To illustrate the effect on process risk, consider CO_2_ capture from flue gas. In many designs, moisture is removed upstream of the adsorption process.^[^ ^171^ ^]^ Where adsorbents are sensitive to moisture and irreversibly damaged, an upstream failure of the drying unit poses the risk of destroying the adsorbent bed and inducing complete replacement. In contrast, adsorbents that are not irreversibly damaged can be salvaged through (relatively) inexpensive regeneration operations.

Therefore, an important, and probably minimum, consideration for any material design is that it can handle the conditions, contaminants, or variation in feed composition where it will be used. For example, when integrating with operations such as cryogenic distillation it is unlikely moisture will be present or even at risk of being present.

From examining leading materials identified from the bound analysis, it is evident that comments on stability are rare. Negative stability results do not need to be a paper killer, although relevant comments on stability will be required for any application. Poor stability to certain contaminants would only limit the field of applications of a given adsorbent material and does nothing to detract from the scientific conclusions of a work.

Stability is also sometimes a solvable design problem. For example, water‐stable MOFs exist, such as SIFSIX‐18‐Ni‐ß,^[^ ^174^ ^]^ MUF‐16^[^ ^3^, ^72^ ^]^ and MIL‐100(Fe).^[^ ^175^ ^]^ For MIL‐100(Fe), moisture has even been shown to increase the CO_2_ uptake by increasing the solubility of CO_2_.^[^ ^175^ ^]^

Moisture is only one example of stability requirements for CO_2_/N_2_ separation from flue gas, other contaminants include SO_X_ and NO_X_.^[^ ^78^ ^]^ Other gas separations will have their unique conditions that must either have an engineering/process solution or involve materials resistant to contaminants and non‐ideal conditions. Addressing the stability of adsorbents studied for a specific application would add great practical value to the resulting work.

### 17.3 Sustainability and Veracity of Relevance to Application

Taking a wider viewpoint, the majority of these materials and processes are being developed with the motivation of slowing climate change. Therefore, the environmental impact of these materials should also be considered.

A lifecycle assessment of ZIF‐8 synthesis identified the main environmental weak points as the use of DMF and methanol.^[^ ^176^ ^]^ Green alternatives such as de‐ionized water^[^ ^176^ ^]^ and near supercritical water^[^ ^177^ ^]^ have been suggested. These alternatives appear to be feasible with BASF producing MOFs (including HKUST‐1, MOF‐177, Fe‐BTC, ZIF‐8 and MIL‐53(Al)) on a large scale using an electrochemical synthesis method/aqueous reaction medium, without organic solvents.^[^ ^78^, ^178^ ^]^ Synthesis methods like these are key to competing on a sustainability level with materials like Zeolite 13X, which does not require any organic solvents.^[^ ^179^, ^180^, ^181^, ^182^ ^]^ Sustainable, scalable, and low‐cost, production methods are therefore an area of exploration for the field.

### 17.4 Preparing Data for Unit Operation Modeling

Ultimately, many scientists would like to see their new adsorbent materials utilized in the real world. Unit operation modeling, i.e., how the adsorbent would perform in an idealized process, is a useful next step before conducting real‐world, equipment‐intensive experiments with the adsorbent.

Many processes are available, including breakthrough pressure swing adsorption (PSA), vacuum swing adsorption (VSA), temperature swing adsorption (TSA), and simulated moving bed (SMB) processes. Universally, the information required for initial process simulations is reliable isotherm data collected over at least 3 temperatures. The isotherms should be collected over the pressure range relevant to the process, i.e., for vacuum swing adsorption 0–1 bar is suitable, and for PSA processes up to 40 bar could be required.

The information provided by an initial process model will provide information about the amount of adsorbent required, allowing rough estimation of the capital costs, and the minimum energy requirements, allowing rough estimation of the operating costs. This information can be used to rapidly decide if a new adsorbent is promising for a specific gas separation and if further studies would be pursued for practical application or scientific discovery.

### 17.5 Summary

The purpose of this section has been to inspire researchers who strive to have their adsorbent materials applied to real‐world separation problems. It does nothing to detract from scientific inquiry that has produced such a wide array of potential adsorbent materials. Hopefully, this section has highlighted that beyond the simple metrics of capacity, selectivity, and heat of adsorption, a diverse number of other practical aspects can be considered during scientific studies and provide rich and valuable advances in designs to reduce cost and increase chemical stability. From our analysis, it is unclear what tradeoffs would exist for these additional properties—or even how (or if) they could be effectively metricized. In the future, it will be materials that strike a good balance between their adsorption metrics and practical considerations that drive the truly impressive practical breakthroughs.

## 18 Conclusions 

We have applied a bound visualization tool to the field of adsorption for gas separation—providing insights to materials scientists interested in the leading design strategies for inspiration to their own material innovations. The bound visualization is also useful to process engineers for identifying adsorbent materials for process modeling and further investigation into cost, stability, and sorption kinetics.

The simple adsorption metrics of capacity, selectivity, and heat of adsorption have traditional design trade‐offs, where it is difficult to maximize one without another suffering as a result. The bound visualizations allowed for quick comparison of these trade‐offs and observation of the trends that best balance (and in some cases, break) them. **Table** **6** summarizes these results for each gas pair, comparing the theoretical design strategies to the best observed strategies. Interactive versions of these bound visualizations can be accessed using the online resource (https://adsorbents.canterbury.ac.nz).

**Table 6:** Summary of physical parameters and best strategies for designing adsorbent materials for each gas separation pair. Inferred design strategies are based on the physical properties of the gas pair. Best current strategies were identified from our analysis of the materials that perform best against the identified upper and lower bounds

Gas Pair	Kinetic diameters [Å]	Polarizability ×10^−25^ [cm^3^]	Quadrupole ×10^−26^ moment [esu cm^2^]	Inferred design strategies	Best current strategies
CO_2_/N_2_	3.3/3.64	26.5/17.6	4.3/1.52	Size‐sieving, pore‐size matching, and CO_2_ interaction strength	Open metal sites with well‐matched cavity sizes, balancing of interaction strength, and high surface area carbons
CO_2_/CH_4_	3.3/3.8	26.5/26	4.3/0	Pore‐size matching and CO_2_ quadrupole interaction strength	Cavity size matching to enhance CO_2_ adsorption, core–shell strategies to create mesopores
CO_2_/H_2_	3.3/2.89	26.5/7.9	4.3/0.66	CO_2_ interaction strength	Open metal sites
CH_4_/H_2_	3.8/2.89	26/7.9	0/0.66	CH_4_ polarizability interaction strength	Nitrogen‐doped activated carbons
CH_4_/N_2_	3.8/3.64	26/17.6	0/1.52	CH_4_ polarizability interaction strength	Ultra‐microporous and nitrogen‐doped activated carbons
N_2_/CH_4_	3.64/3.8	17.6/26	1.52/0	N_2_ π* orbitals, size sieving	Open metal sites
O_2_/N_2_	3.46/3.64	13/17.6	0.4/1.52	Size‐sieving (kinetic selectivity) and interaction with additional valence electrons	Physisorption, open metal sites and valence electron interaction
N_2_/O_2_	3.64/3.46	17.6/13	1.52/0.4	N_2_ interaction strength	Interaction with N_2_ quadrupole
Xe/Kr	4.1/3.6	4.04/2.48	0/0	Pore size matching and Xe polarizability interaction strength	Pore size matching of Xe and adsorption to open metal sites

The practical application of adsorbent materials is to reduce the energy use and capital cost of gas separation processes. Therefore, factors such as cost, stability, reproducibility, and sustainability are important to consider in addition to simple adsorption metrics. The bound visualizations in this work do not consider these factors, and we suggest that for gas separations such as CO_2_/N_2_ and CO_2_/CH_4_, where over a thousand adsorbent materials have been reported, it is the additional considerations that require scientific investigation to promote the application of adsorbent materials in industry.

We hope that this work inspires advances in both adsorbent materials design and process engineering and that, as a result, adsorbent materials and their processes can be accelerated toward increasing the energy and capital efficiency of the gas separation industry.

## 19 Methods

### 19.1 Data Gathering

4068 references were processed, of this, 1137 references contained data useful to the review. The detailed method for assessing references is contained in Section S1 (Supporting Information). Some key points include:

- Only sources with experimental data for both gases in a pair are considered.
- Capacity and selectivity were recorded as close to 293 K and 100 kPa as possible. To keep the comparison consistent, any data recorded above 110 kPa/333 K or below 273 K was removed from the dataset.
- Selectivity from the IAST method was preferred, where this was not reported, the pure gas uptake ratio was calculated from the reported isotherms.
- Where IAST results were reported for multiple compositions, the equimolar mixture was recorded over any other mixture.
- Materials were classed as including metal sites only if it is explicitly mentioned in the paper, or if the material is well known to have active metal sites. This means that some metal site materials may have been classed as porous or porous (MOF) incorrectly.
- Values were estimated from figures visually. The scale of this review meant digitizing every source or requesting raw experimental data was not a suitable method.
- It is recognized that visual estimation of values may introduce some error to results. In particular, gases with low uptake when plotted on the same figure as gases with high uptake (e.g., H_2_ and CO_2_), the low uptake gas is likely to have more significant error.
- Heat of adsorption was recorded at a loading as close to the recorded capacity as possible. This method may miss spikes in heat of adsorption at low loading, particularly for materials with open metal sites or amine sites where the heat of adsorption may reduce once all of these active sites are saturated. This is recognized for the superior materials that break the bound, where some only break the bound once the initial spike is overcome, these are either considered not a special bound breaking material or discussed otherwise.

## Conflict of Interest

The authors declare no conflict of interest.

## Author Contributions

S.J.E. led the review data gathering process, analyzed the results and prepared the manuscript. M. G.C. conceptualized the idea for the work and contributed to writing the manuscript. M.J.M., S.G., Z.D., H.Z., L.Z., Z.S. and J.W., T.D.B., A.Q., H.T., and N.L. organized and processed references, gathered data, edited the manuscript, and provided insightful observations for the review.

## Supporting information

Supporting Information

Supporting Information

Supporting Information

Supporting Information
