# Supplementary material for: An Upper Bound Visualization of Design Trade‐Offs in Adsorbent Materials for Gas Separations: CO2, N2, CH4, H2, O2, Xe, Kr, and Ar Adsorbents
Source: Adv Sci (Weinh). 2023 Jan 16;10(8):2206437. doi: 10.1002/advs.202206437 (PMC10015871; doi:10.1002/advs.202206437)
Supplement: Supplementary file 1 — Supporting Information [file ADVS-10-2206437-s002.pdf]

ADVANCED SCIENCE
Open Access

Supporting Information

for *Adv. Sci.*, DOI 10.1002/advs.202206437

An Upper Bound Visualization of Design Trade-Offs in Adsorbent Materials for Gas Separations: CO_2, N_2, CH_4, H_2, O_2, Xe, Kr, and Ar Adsorbents

*Samuel J. Edens, Michael J. McGrath, Siyu Guo, Zijuan Du, Hemin Zhou, Lingshan Zhong, Zuhao Shi, Jieshuo Wan, Thomas D. Bennett, Ang Qiao, Haizheng Tao, Neng Li and Matthew G. Cowan**

Supporting Information

An upper bound visualization of design trade-offs in adsorbent materials for gas separations: CO_2, N_2, CH_4, O_2, H_2, Xe, Kr and Ar adsorbents.

*Samuel J. Edens,[1] Michael J. McGrath,[1] Siyu Guo,[2] Zijuan Du,[2] Hemin Zhou,[2] Lingshan Zhong,[2] Zuhao Shi,[2,3] Jieshuo Wan,[2,3] Thomas D. Bennett,[4] Ang Qiao,[2] Haizheng Tao,[2] Neng Li,[2,3] Matthew G. Cowan[1,]**

[1] Department of Chemical and Process Engineering and MacDiarmid Institute for Advanced Materials and Nanotechnology, University of Canterbury, 8041, New Zealand
* E-mail: matthew.cowan@canterbury.ac.nz

[2] State Key Laboratory of Silicate Materials for Architectures, Wuhan University of Technology, Wuhan 430070, China

[3] Shenzhen Research Institute of Wuhan University of Technology, Shenzhen 518000, China

[4] Department of Materials Science and Metallurgy, University of Cambridge, 27 Charles Babbage Road, Cambridge CB3 0FS, UK

Contents

1 Data gathering method 1
1.1 Literature search 1
1.2 Data extraction notes 1
1.3 Data gathered 4
1.3.1 General information 4
1.3.2 Selectivity and capacity 4
1.3.3 Heat of adsorption 4
2 Physical bound explanation 5
2.1 Bound parameters 5
2.2 Minimum heat of adsorption 7
3 Other gas pairs 10
4 Choice of linear or log axis 13
5 Isotherm reproducibility 15
5.1 Isotherm sources 15
5.2 HKUST-1 16
5.3 UiO-66 17
5.4 ZIF-8 18
6 Cost analysis 19
6.1 Zeolite 13X cost for literature example[51] 19
6.2 HKUST-1 commercial sample purchase 19
6.3 Cost to purchase synthesized material from Sigma-Aldrich 19
6.4 Cost to purchase raw materials for synthesis from Sigma-Aldrich 19
7 References 21

List of tables

Table S1: Gas physical parameters and their minimum heat of adsorption. 5
Table S2: Cost (per gram) to synthesize HKUST-1 from Sigma-Aldrich raw materials...................... 20
Table S3: Cost (per kilogram) to synthesize Zeolite 13X from Sigma-Aldrich raw materials. 20

List of figures

Figure S1: Reference processing flowchart (Part 1). 2
Figure S2: Reference processing flowchart (Part 2) 3
Figure S3: Correlation between absolute difference in kinetic diameter and uptake vs selectivity bound parameters a) 'a' and b) 'b'. Blue = CO_2/N_2, orange = CO_2/CH_4, green = CO_2/H_2, red = CH_4/H_2, purple = O_2/N_2, brown = Xe/Kr. 6
Figure S4: Correlation between absolute difference in kinetic diameter and uptake vs heat of adsorption bound parameters a) 'm' and b) 'c'. Blue = CO_2/N_2, orange = CO_2/CH_4, green = CO_2/H_2, red = CH_4/H_2, purple = O_2/N_2, brown = Xe/Kr. 6
Figure S5: Correlation between absolute difference in kinetic diameter and selectivity vs heat of adsorption bound parameters a) 'g' and b) 'f'. Blue = CO_2/N_2, orange = CO_2/CH_4, green = CO_2/H_2, red = CH_4/H_2, purple = O_2/N_2, brown = Xe/Kr. 6
Figure S6: Correlation between minimum heat of adsorption and kinetic diameter. Blue = CO_2, orange = N_2, green = CH_4, red = H_2, purple = O_2, brown = Xe, pink = Kr, grey = Ar, olive = C_2H_4, cyan = C_2H_6, black = C_3H_6. 7
Figure S7: Correlation between minimum heat of adsorption and polarizability. Blue = CO_2, orange = N_2, green = CH_4, red = H_2, purple = O_2, brown = Xe, pink = Kr, grey = Ar, olive = C_2H_4, cyan = C_2H_6, black = C_3H_6. 7
Figure S8: Correlation between minimum heat of adsorption and quadrupole moment. Blue = CO_2, orange = N_2, green = CH_4, red = H_2, purple = O_2, brown = Xe, pink = Kr, grey = Ar, olive = C_2H_4, cyan = C_2H_6, black = C_3H_6. 8
Figure S9: Correlation between minimum heat of adsorption and specific gas constant. Blue = CO_2, orange = N_2, green = CH_4, red = H_2, purple = O_2, brown = Xe, pink = Kr, grey = Ar, olive = C_2H_4, cyan = C_2H_6, black = C_3H_6. 8
Figure S10: Correlation between minimum heat of adsorption and square root of molar mass. Blue = CO_2, orange = N_2, green = CH_4, red = H_2, purple = O_2, brown = Xe, pink = Kr, grey = Ar, olive = C_2H_4, cyan = C_2H_6, black = C_3H_6. 9
Figure S11: Correlation between minimum heat of adsorption and molar mass. Blue = CO_2, orange = N_2, green = CH_4, red = H_2, purple = O_2, brown = Xe, pink = Kr, grey = Ar, olive = C_2H_4, cyan = C_2H_6, black = C_3H_6. 9
Figure S12: Xenon-oxygen bound visualization - 19 materials. 10
Figure S13: Xenon-nitrogen bound visualization - 49 materials. 10
Figure S14: Krypton-oxygen bound visualization - 18 materials. 10
Figure S15: Krypton-nitrogen bound visualization - 48 materials. 11
Figure S16: Argon-oxygen bound visualization - 57 materials. 11
Figure S17: Argon-nitrogen bound visualization - 50 materials. 11
Figure S18: Xenon-argon bound visualization - 37 materials. 11
Figure S19: Krypton-argon bound visualization - 41 materials. 12
Figure S20: Illustration of how plotting selectivity on a log-axis results in a straight line. 13
Figure S21: Carbon dioxide – nitrogen plots with heat of adsorption on a log-axis. 13
Figure S22: Carbon dioxide – methane plots with heat of adsorption on a log-axis. 14
Figure S23: Carbon dioxide – hydrogen plots with heat of adsorption on a log-axis. 14
Figure S24: Methane – hydrogen plots with heat of adsorption on a log-axis. 14
Figure S25: Methane – nitrogen plots with heat of adsorption on a log-axis. 14
Figure S26: Oxygen – nitrogen plots with heat of adsorption on a log-axis. 15
Figure S27: Nitrogen – oxygen plots with heat of adsorption on a log-axis. 15
Figure S28: Xenon – krypton plots with heat of adsorption on a log-axis. 15
Figure S29: HKUST-1 CO_2 298K isotherm reproducibility. Darker gradient indicates higher activation temperature, with orange indicating an unknown activation temperature. 16

Figure S30: HKUST-1 CH_4 298K isotherm reproducibility. Darker gradient indicates higher activation temperature, with orange indicating an unknown activation temperature............................16
Figure S31: HKUST-1 N_2 298K isotherm reproducibility. Darker gradient indicates higher activation temperature, with orange indicating an unknown activation temperature. 16
Figure S32: UiO-66 CO_2 298K isotherm reproducibility. Darker gradient indicates higher activation temperature, with orange indicating an unknown activation temperature. 17
Figure S33: UiO-66 CH_4 298K isotherm reproducibility. Darker gradient indicates higher activation temperature, with orange indicating an unknown activation temperature. 17
Figure S34: UiO-66 N_2 298K isotherm reproducibility. Darker gradient indicates higher activation temperature, with orange indicating an unknown activation temperature. 17
Figure S35: ZIF-8 CO_2 298K isotherm reproducibility. Darker gradient indicates higher activation temperature, with orange indicating an unknown activation temperature. 18
Figure S36: ZIF-8 CH_4 298K isotherm reproducibility. Darker gradient indicates higher activation temperature, with orange indicating an unknown activation temperature. 18
Figure S37: ZIF-8 N_2 298K isotherm reproducibility. Darker gradient indicates higher activation temperature, with orange indicating an unknown activation temperature. 18

1 Data gathering method

This section outlines the literature search and data processing methods used.

1.1 Literature search

The search was performed on 16/11/2021 using the ‘Research topic’ function of SciFinder.

The following keywords were used:

“CO2 N2 adsorption” “carbon dioxide nitrogen adsorption” “CO2 H2 adsorption” “carbon dioxide hydrogen adsorption” “CO2 CH4 adsorption” “carbon dioxide methane adsorption” “CO2 H2 adsorption” “carbon dioxide hydrogen adsorption” “CH4 H2 adsorption” “methane hydrogen adsorption” “argon adsorption” “xenon adsorption” “krypton adsorption” “O2 N2 adsorption” “oxygen nitrogen adsorption”

Filtered by term “isotherm”, language “English”, and document type “Journal” “Book” “Review”. Duplicates were removed and the list compiled, resulting in 4068 references. The results were judged according to the flowsheet in Figure S1 and Figure S2 and data from the relevant papers were recorded.

1.2 Data extraction notes

Papers required experimental results for at least two gases to be considered in the review. This meant that papers that only showed simulations results or only reported the adsorption of a single gas were not considered.

Where values were extracted from figures, they were estimated by eye. The most accurate method would be to digitize the plots to extract the values of interest, however, the scale of this review meant that this was not feasible.

Students reviewing references for this review were first taken through 13 example papers to help them understand the process. The examples were split into two sections. The first section was worked through in detail with the students one on one, the second section was completed by the student on their own afterwards. The second section aimed to confirm the students understanding. After successful completion of the second section, they began work on references for the review.

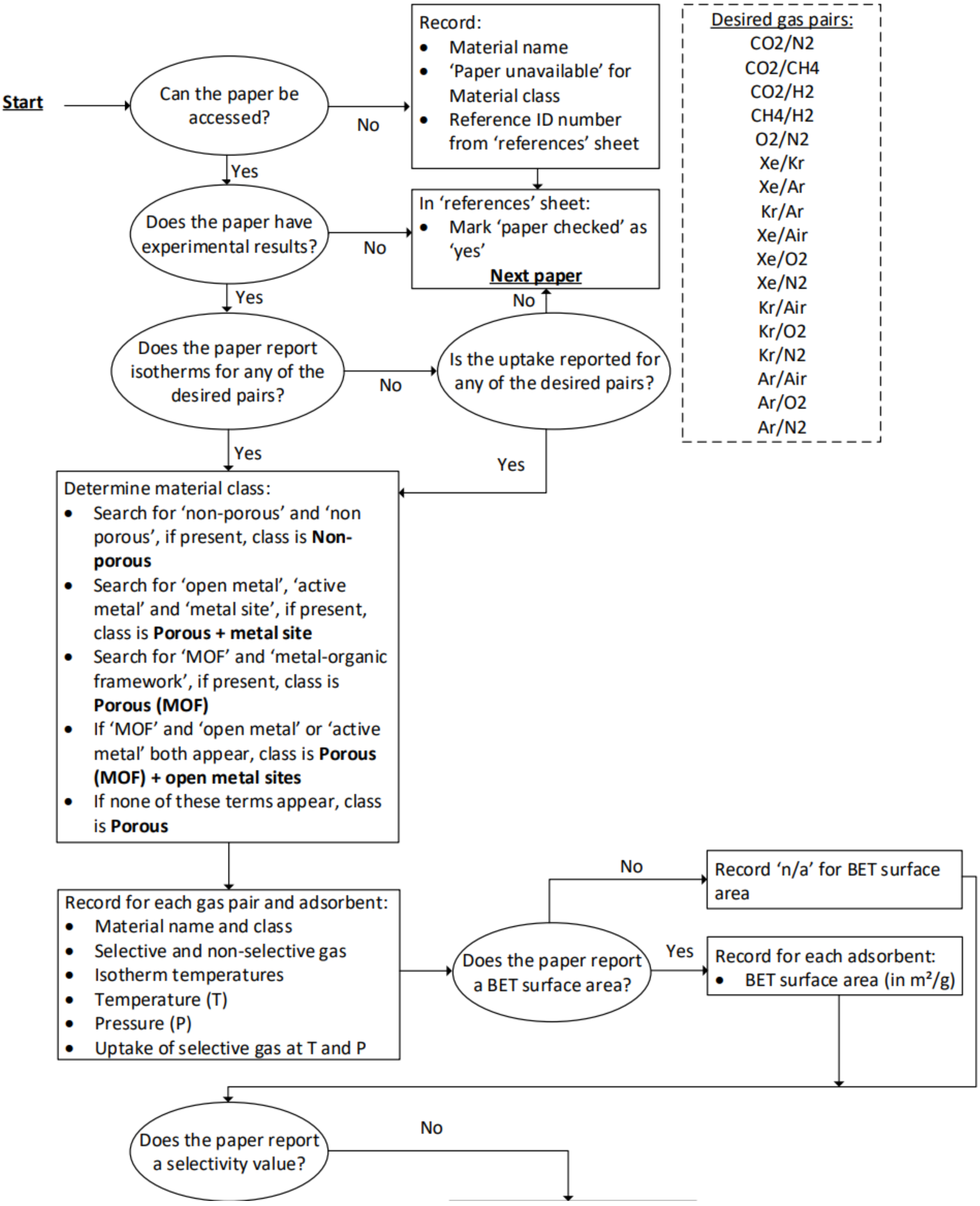

Figure S1: Reference processing flowchart (Part 1).

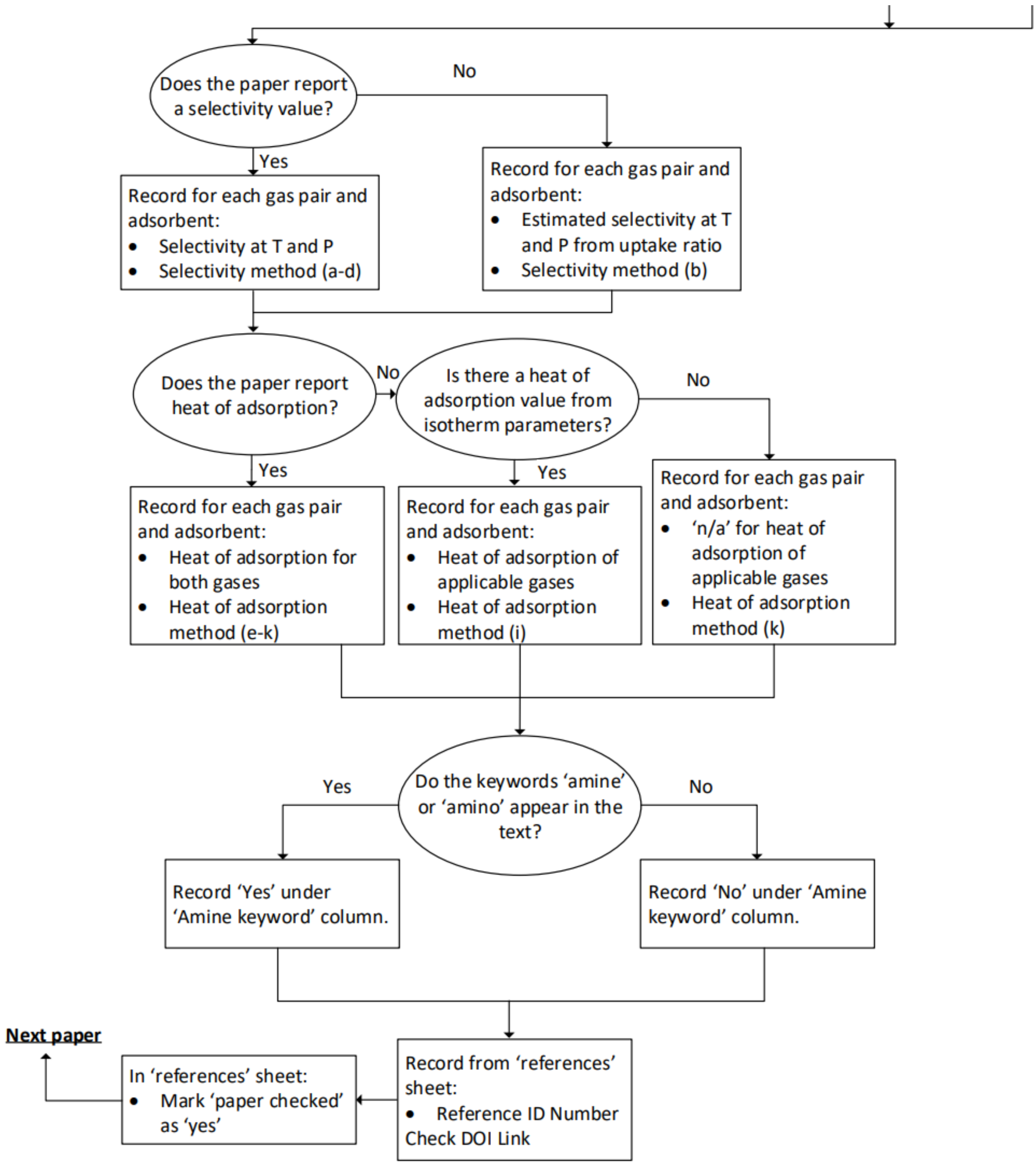

Figure S2: Reference processing flowchart (Part 2)

1.3 Data gathered

This section describes the specific data gathered for each material/gas separation pair.

1.3.1 General information

- Adsorbent class (Porous, non-porous, porous+metal site, MOF, MOF+metal site).
- Selective and non-selective gas.
- Isotherm temperatures.
- BET surface area

1.3.2 Selectivity and capacity

- Temperature and pressure that capacity and selectivity correspond to. This temperature was chosen as close to 293 K and 100 kPa as possible to give a fair comparison between materials.
- Pure gas capacity for selective gas.
- Selectivity.
- The method used for calculating selectivity was also recorded. Selectivity method:
    a) Using IAST for an equimolar amount of the two gases (preferred over uptake ratio).
    b) From reported adsorption capacities of both gases (uptake ratio).
        - $S = \frac{q_{selective\ gas}}{q_{non-selective\ gas}}$
    c) Measured/calculated using an unconventional method.
    d) Measurement method not shown/available.
    l) Henrys law.

In the case that a paper did not report a selectivity value, it was calculated manually using the reported gas uptake and the uptake ratio method.

1.3.3 Heat of adsorption

- Heat of adsorption for both the selective and non-selective gas.
- The heat of adsorption was recorded at the Temperature and Pressure previously recorded for selectivity and capacity. If the heat of adsorption was reported as a function of loading, the loading value was chosen as the loading at the Temperature and Pressure previously recorded for selectivity and capacity.
- If the loading was below/above the minimum/maximum loading value of the heat of adsorption curve, report the heat of adsorption at the minimum/maximum loading shown on the heat of adsorption curve.
- 'n/a' was recorded if the paper did not report heat of adsorption for the gas of interest
- Heat of adsorption method:
    e) Clausius-Clapeyron method.
    f) Virial method.
    g) From temperature programmed desorption (TPD).
    h) From differential scanning calorimetry (DSC).
    i) From isotherm model fit.
    j) Measured/calculated using an unconventional method.
    k) Measurement method not shown/available.
    m) Vant Hoff method
    n) Gibbs-helmholtz method

2 Physical bound explanation

This section gives some examples of attempts to correlate the empirical bound parameters and minimum heat of adsorption to physical parameters of each gas. The physical parameters used for each gas are outlined in Table S1 below.

Table S1: Gas physical parameters and their minimum heat of adsorption.

	CO_2	N_2	CH_4	H_2	O_2	Xe	Kr	Ar	C_2H_4	C_2H_6	C_3H_6
**Kinetic diameter (Å)**	3.30 [1]	3.64 [1]	3.80 [1]	2.89 [1]	3.46 [2]	4.10 [3]	3.60 [3]	3.40 [1]	3.90 [4]	3.80 [5]	4.50 [4]
**Polarizability $\times 10^{-25}$ (cm^3)**	26.5 [1]	17.6 [1]	26 [1]	7.9 [1]	13 [6]	4.04 [3]	2.48 [3]	16.2 [1]	42 [7]	44 [7]	63 [7]
**Quadrupole $\times 10^{-26}$ moment ($esu.cm^2$)**	4.3 [1]	1.52 [1]	0 [1]	0.66 [1]	0.4 [8]	0 [1]	0 [1]	0 [1]	2[8]	0.8[8]	0 [7]
**Specific gas constant (J/(kgK))** [9]	188.92	297	518.28	4126	260	63.3	99.2	208.13	296.38	276.51	197.59
**Molar mass (g/mol)**	44	28	16	2	32	131.3	83.8	39.95	28	30	42
**Minimum heat of adsorption (kJ/mol)**	18	16.2	16.0	13.0	12.5	17.5	14.5	11.0	22.8	21.0	28.0

2.1 Bound parameters

The upper and lower bounds for each gas pair are described by equation S1 (uptake vs selectivity upper bound), equation S2 (uptake vs heat of adsorption lower bound) and equation S3 (selectivity vs heat of adsorption lower bound).

$$S = \frac{a}{\exp(bq)} \#S1$$

$$H = mq + c \#S2$$

$$H = g\ln(S) + f \#S3$$

Where S is selectivity in mol·mol^{-1}, q is capacity in mol·kg^{-1} and H is heat of adsorption in kJ·mol^{-1}. a, b, m, c, f and g are the fitting parameters for each bound.

Figure S3, Figure S4 and Figure S5 below show the attempted correlation between these bound parameters for each gas pair and the kinetic diameter difference of the gas pair. There is no clear correlation, other physical parameters such as difference in quadrupole moment or difference in molar mass also showed no convincing physical correlation for the upper and lower bound parameters.

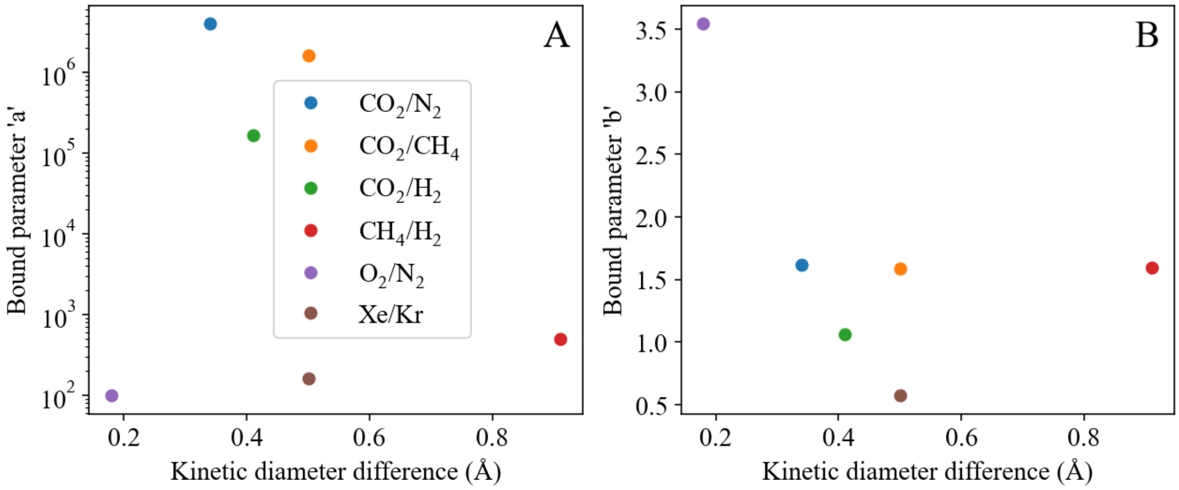

Figure S3: Correlation between absolute difference in kinetic diameter and uptake vs selectivity bound parameters a) 'a' and b) 'b'. Blue = CO_2/N_2, orange = CO_2/CH_4, green = CO_2/H_2, red = CH_4/H_2, purple = O_2/N_2, brown = Xe/Kr.

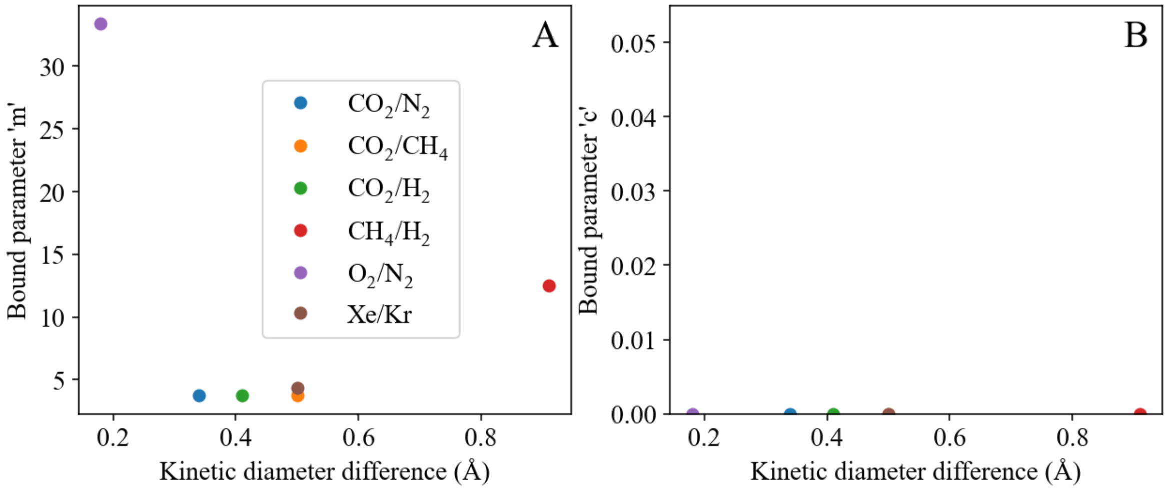

Figure S4: Correlation between absolute difference in kinetic diameter and uptake vs heat of adsorption bound parameters a) 'm' and b) 'c'. Blue = CO_2/N_2, orange = CO_2/CH_4, green = CO_2/H_2, red = CH_4/H_2, purple = O_2/N_2, brown = Xe/Kr.

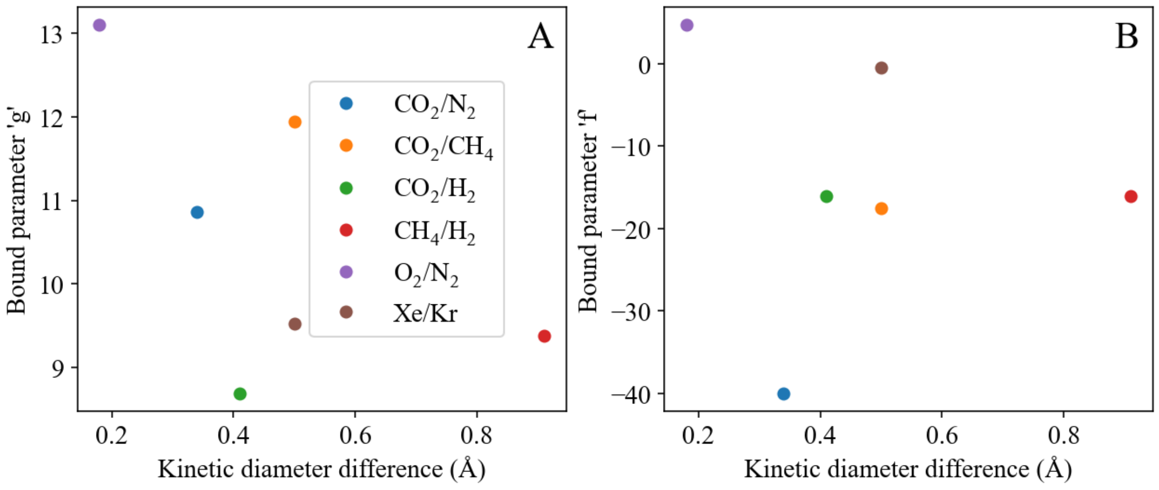

Figure S5: Correlation between absolute difference in kinetic diameter and selectivity vs heat of adsorption bound parameters a) 'g' and b) 'f'. Blue = CO_2/N_2, orange = CO_2/CH_4, green = CO_2/H_2, red = CH_4/H_2, purple = O_2/N_2, brown = Xe/Kr.

2.2 Minimum heat of adsorption

Figure S6, Figure S7, Figure S8, Figure S9, Figure S10 and Figure S11 below show attempts to correlate physical parameters to the estimated minimum heat of adsorption. None of the investigated parameters achieved a plausible correlation.

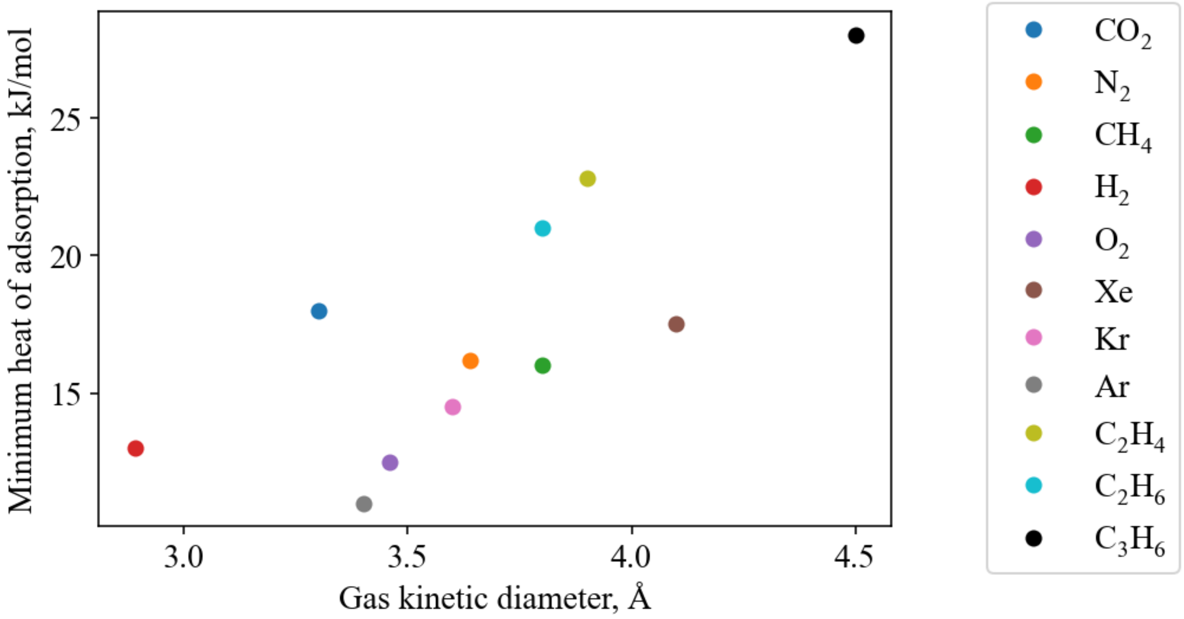

Figure S6: Correlation between minimum heat of adsorption and kinetic diameter. Blue = CO_2, orange = N_2, green = CH_4, red = H_2, purple = O_2, brown = Xe, pink = Kr, grey = Ar, olive = C_2H_4, cyan = C_2H_6, black = C_3H_6.

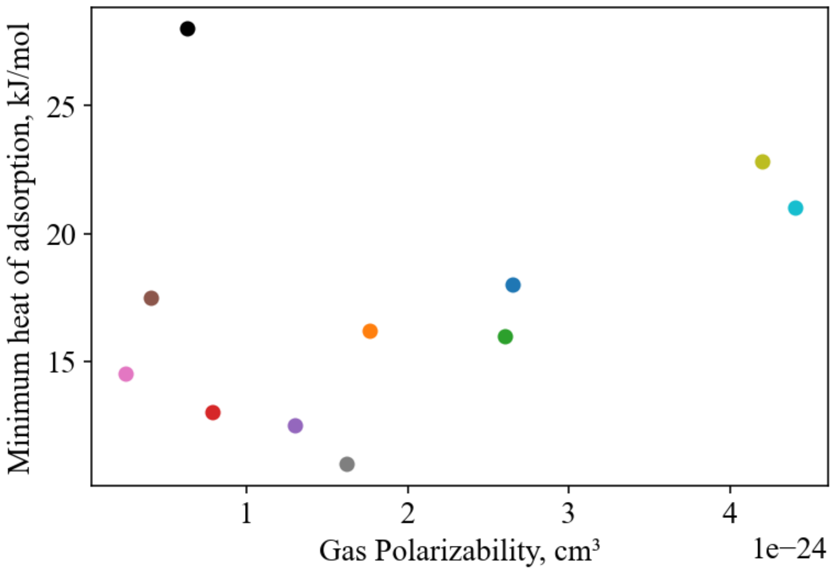

Figure S7: Correlation between minimum heat of adsorption and polarizability. Blue = CO_2, orange = N_2, green = CH_4, red = H_2, purple = O_2, brown = Xe, pink = Kr, grey = Ar, olive = C_2H_4, cyan = C_2H_6, black = C_3H_6.

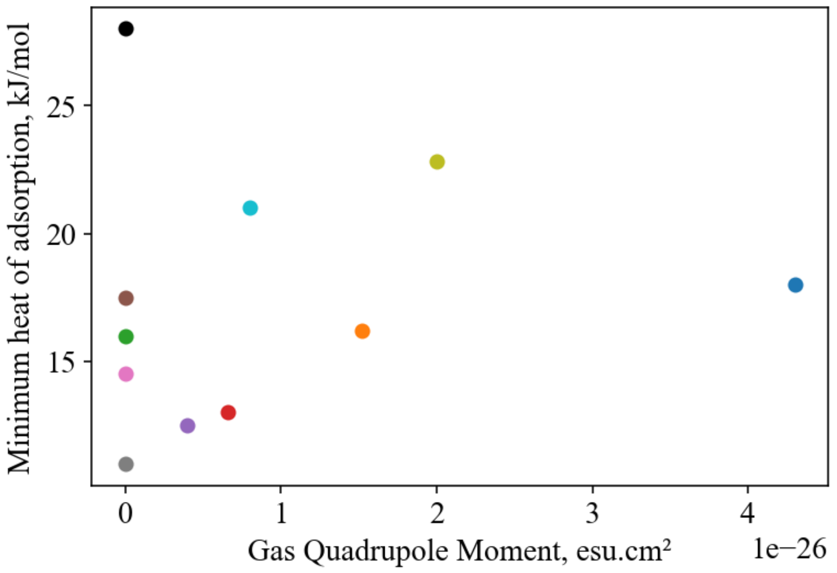

Figure S8: Correlation between minimum heat of adsorption and quadrupole moment. Blue = CO_2, orange = N_2, green = CH_4, red = H_2, purple = O_2, brown = Xe, pink = Kr, grey = Ar, olive = C_2H_4, cyan = C_2H_6, black = C_3H_6.

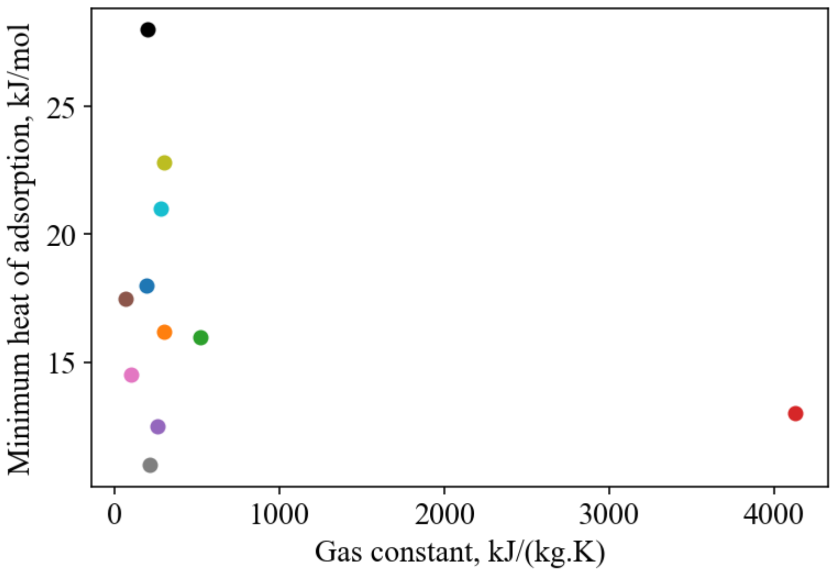

Figure S9: Correlation between minimum heat of adsorption and specific gas constant. Blue = CO_2, orange = N_2, green = CH_4, red = H_2, purple = O_2, brown = Xe, pink = Kr, grey = Ar, olive = C_2H_4, cyan = C_2H_6, black = C_3H_6.

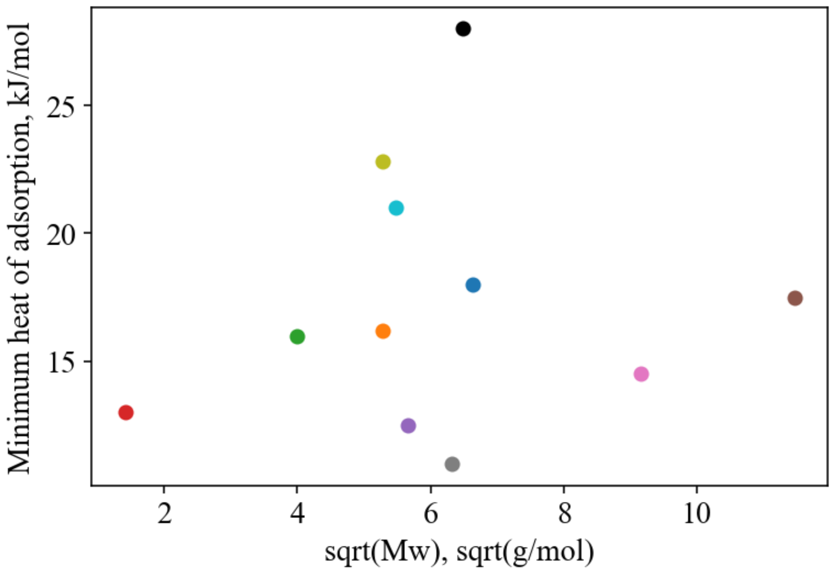

Figure S10: Correlation between minimum heat of adsorption and square root of molar mass. Blue = CO_2, orange = N_2, green = CH_4, red = H_2, purple = O_2, brown = Xe, pink = Kr, grey = Ar, olive = C_2H_4, cyan = C_2H_6, black = C_3H_6.

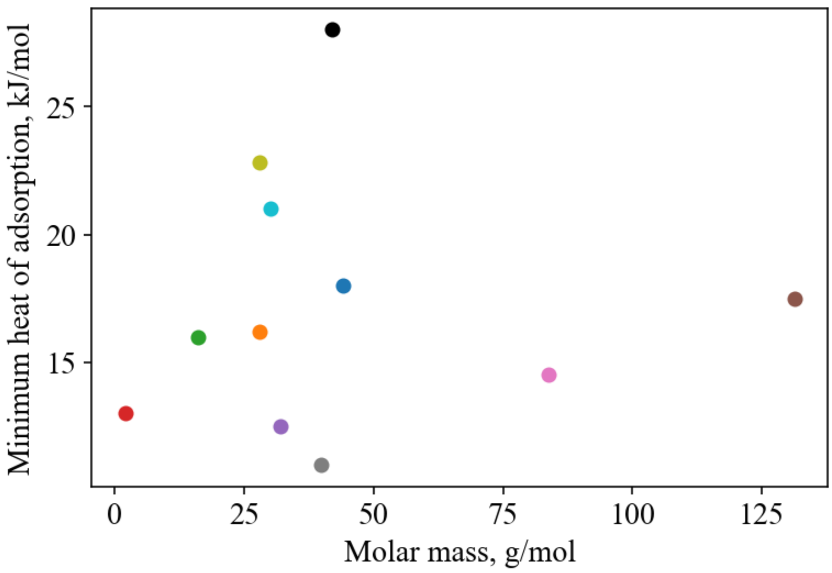

Figure S11: Correlation between minimum heat of adsorption and molar mass. Blue = CO_2, orange = N_2, green = CH_4, red = H_2, purple = O_2, brown = Xe, pink = Kr, grey = Ar, olive = C_2H_4, cyan = C_2H_6, black = C_3H_6.

3 Other gas pairs

This section includes the bound plots of the gas pairs that did not have enough materials to perform an upper or lower bound analysis.

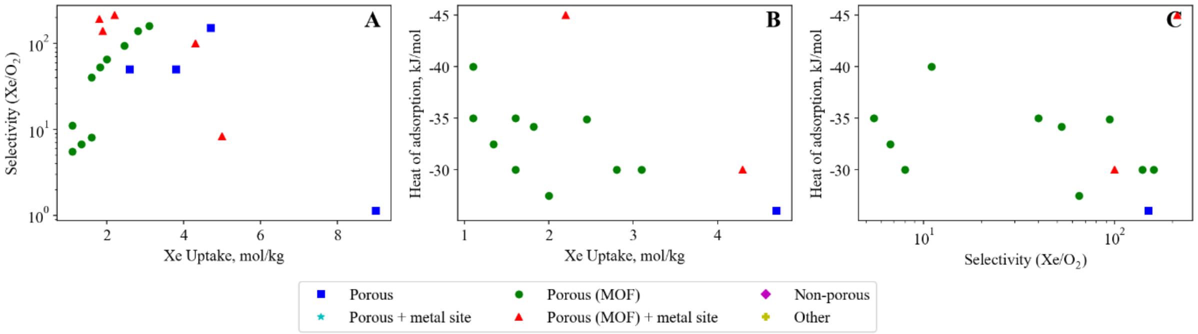

Figure S12: Xenon-oxygen bound visualization - 19 materials.

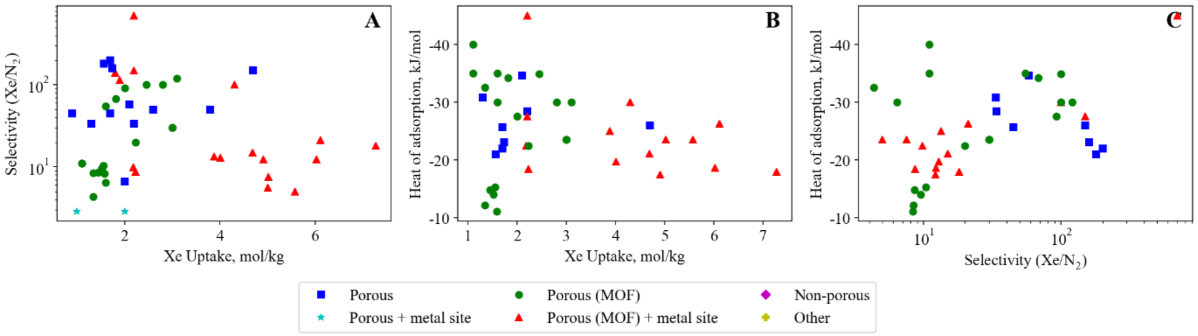

Figure S13: Xenon-nitrogen bound visualization - 49 materials.

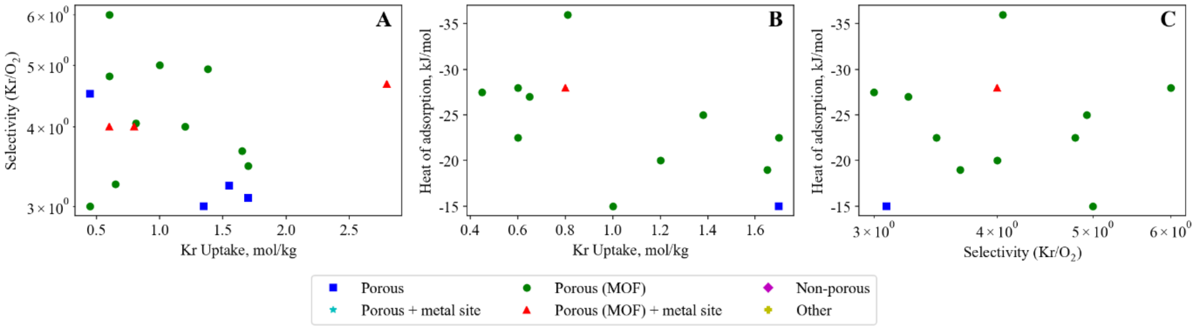

Figure S14: Krypton-oxygen bound visualization - 18 materials.

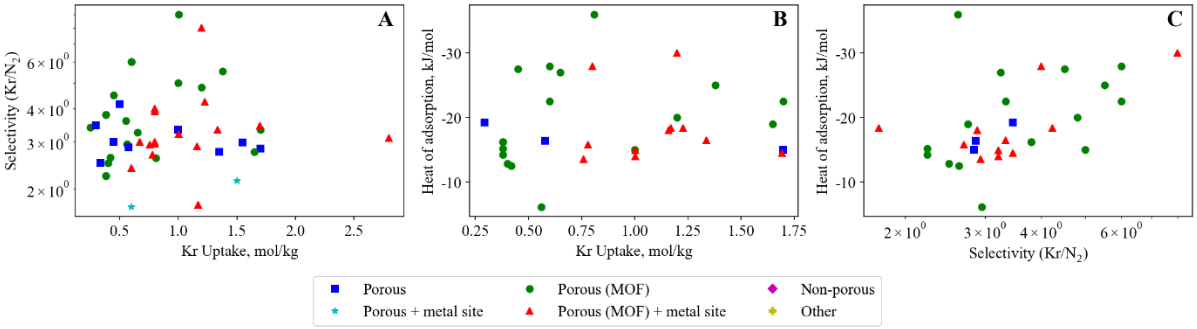

Figure S15: Krypton-nitrogen bound visualization - 48 materials.

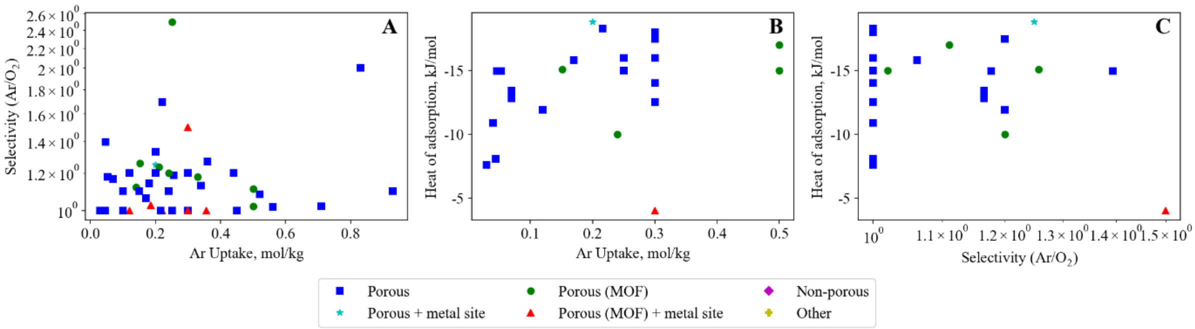

Figure S16: Argon-oxygen bound visualization - 57 materials.

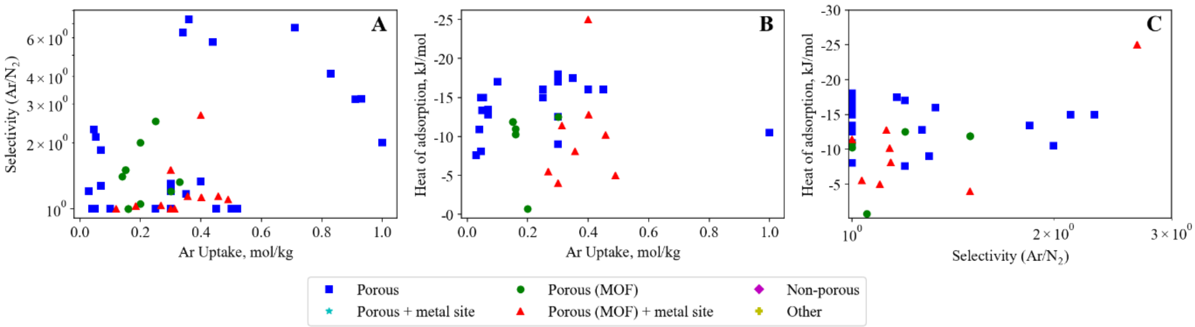

Figure S17: Argon-nitrogen bound visualization - 50 materials.

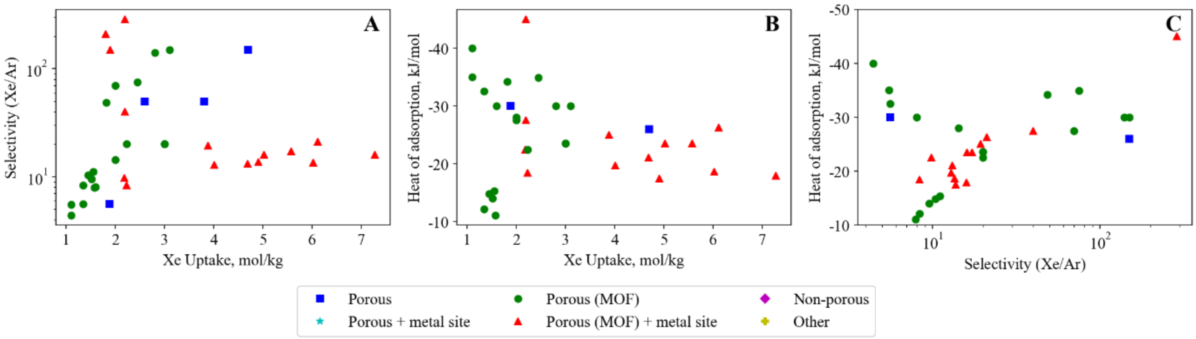

Figure S18: Xenon-argon bound visualization - 37 materials.

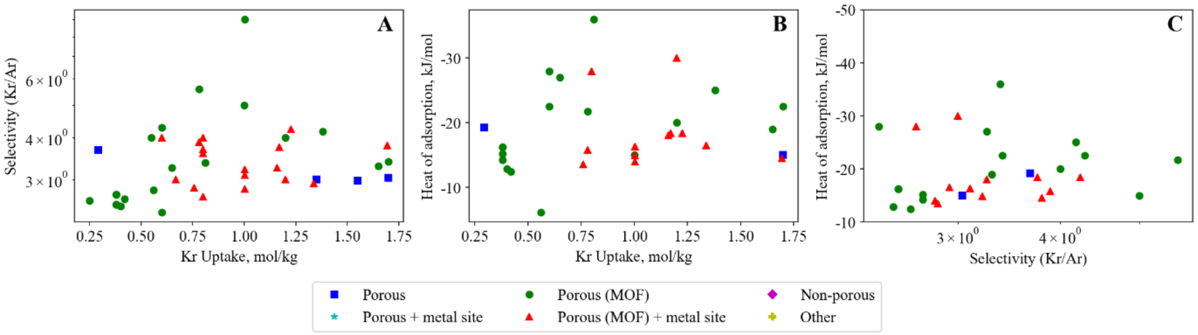

Figure S19: Krypton-argon bound visualization - 41 materials.

4 Choice of linear or log axis

Capacity features on a linear axis as is expected from typical isotherm models, such as the Langmuir isotherm.

In contrast, selectivity for gas A over gas B can be considered as the ratio of their equilibrium constants (K_i) (also known as affinity parameters). The temperature dependence of equilibrium constants are typically expressed as an exponential function of heat of adsorption (Equation S4).

$$K_A = K_{A,0} \exp\left(\frac{-\Delta H_{ads,A}}{R}\left(\frac{1}{T} - \frac{1}{T_0}\right)\right) \#S4$$

This can be simplified for illustrative purposes,

$$Selectivity = \exp(x) \ \#S5$$

Plotting equation S5 over a range of hypothetical x values gives the curve shape in Figure S20A. Applying a log axis to Figure S20A results in Figure S20B and a linear (straight-line) plot. The upper and lower bound visualization in this work rely on linear bounds, therefore it is appropriate to use a log-axis for selectivity.

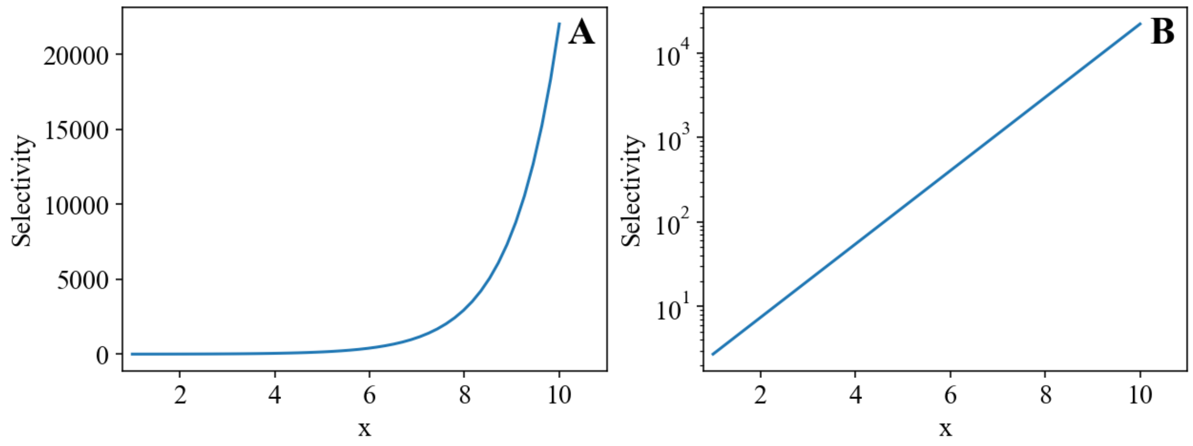

Figure S20: Illustration of how plotting selectivity on a log-axis results in a straight line.

Heat of adsorption features on a linear axis in the main text because the small range of values are adequately displayed on a linear axis. However, this section presents the bound visualizations with heat of adsorption on a log-axis for reader interest.

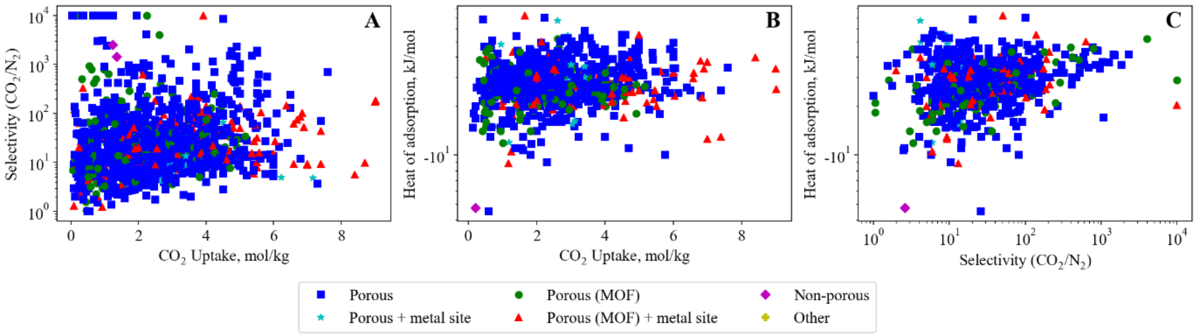

Figure S21: Carbon dioxide – nitrogen plots with heat of adsorption on a log-axis.

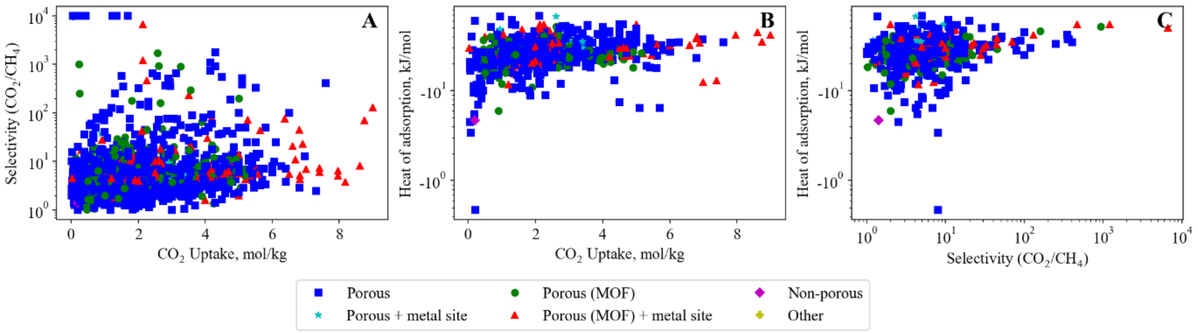

Figure S22: Carbon dioxide – methane plots with heat of adsorption on a log-axis.

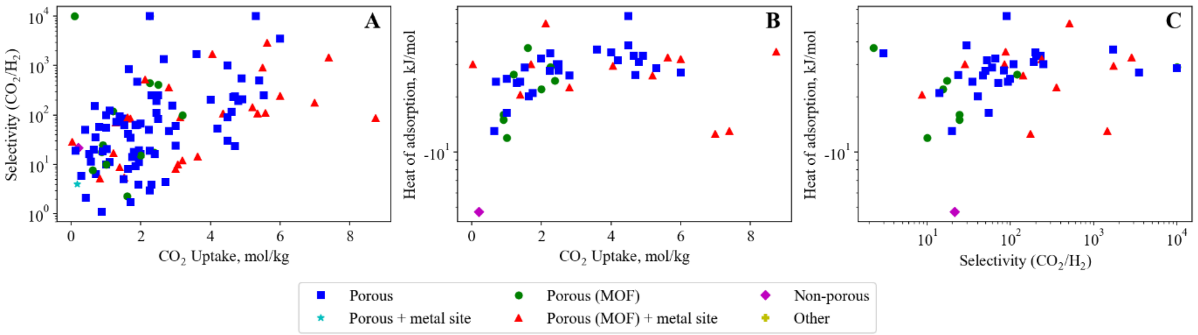

Figure S23: Carbon dioxide – hydrogen plots with heat of adsorption on a log-axis.

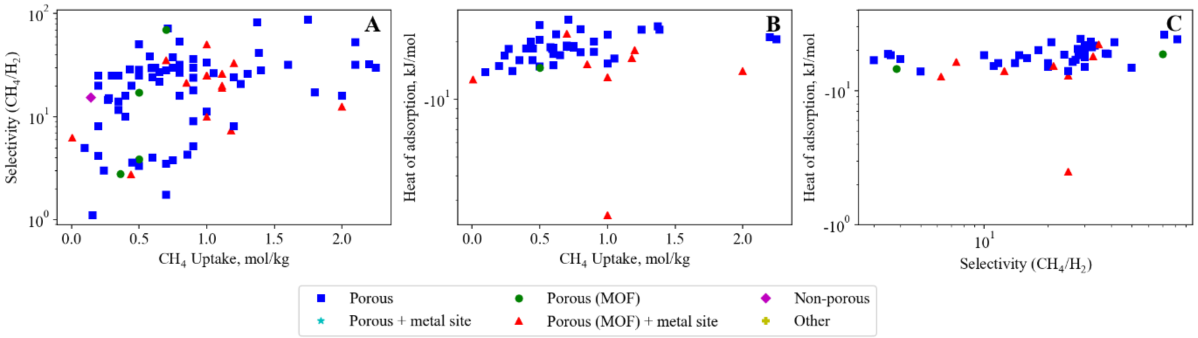

Figure S24: Methane – hydrogen plots with heat of adsorption on a log-axis.

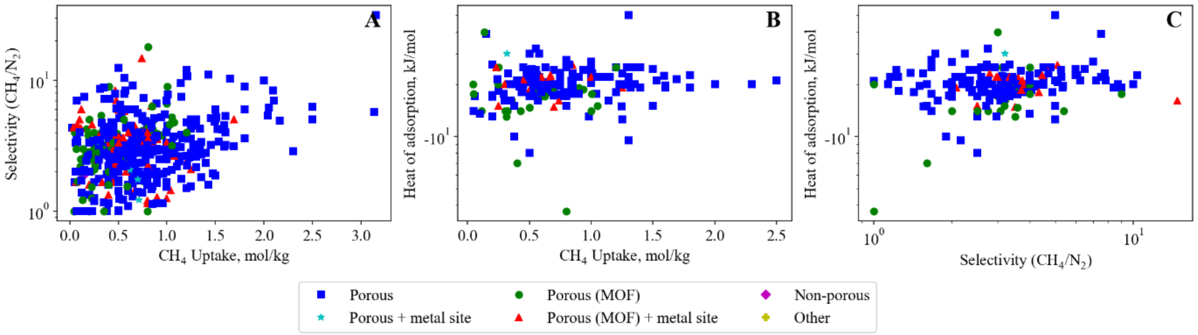

Figure S25: Methane – nitrogen plots with heat of adsorption on a log-axis.

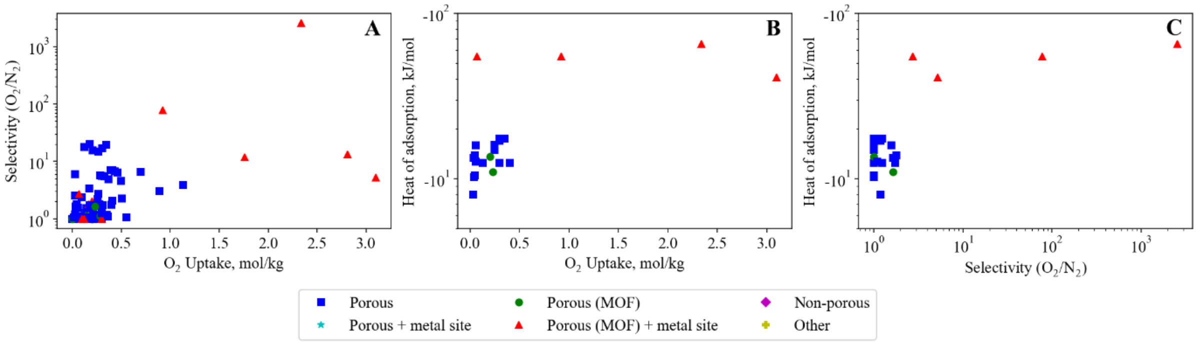

Figure S26: Oxygen – nitrogen plots with heat of adsorption on a log-axis.

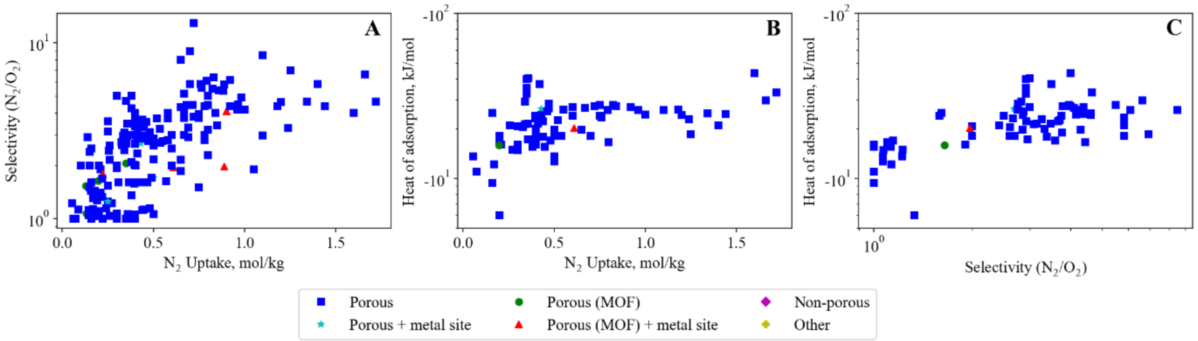

Figure S27: Nitrogen – oxygen plots with heat of adsorption on a log-axis.

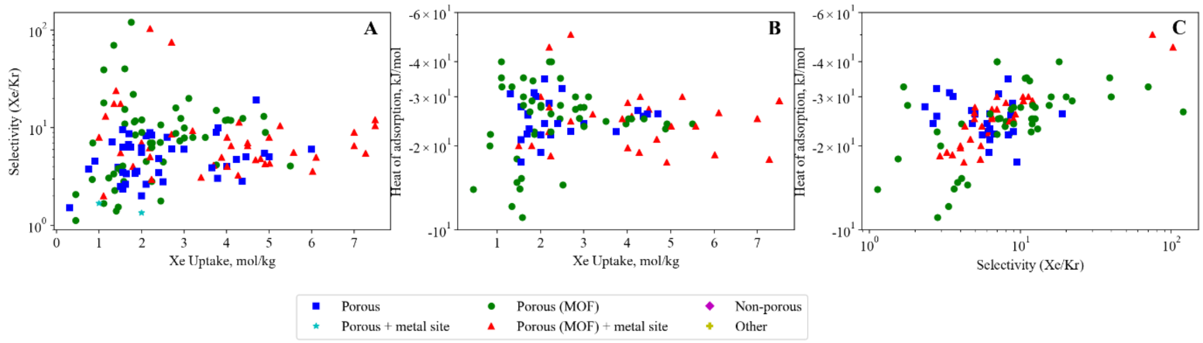

Figure S28: Xenon – krypton plots with heat of adsorption on a log-axis.

5 Isotherm reproducibility

This section includes the references for isotherm reproducibility sources and the isotherm reproducibility plots of HKUST-1, UiO-66 and ZIF-8.

5.1 Isotherm sources

298K Zeolite 13X isotherm references.[10–25]

298K HKUST-1 isotherm references.[10,25–35]

298K UiO-66 isotherm references.[36–43]

298K ZIF-8 isotherm references.[22,26,27,34,44–50]

5.2 HKUST-1

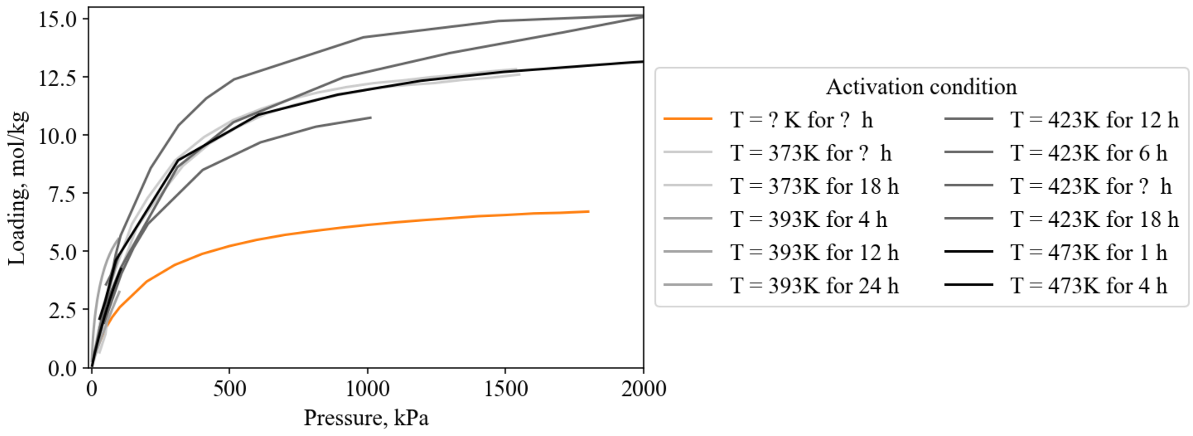

Figure S29: HKUST-1 CO_2 298K isotherm reproducibility. Darker gradient indicates higher activation temperature, with orange indicating an unknown activation temperature.

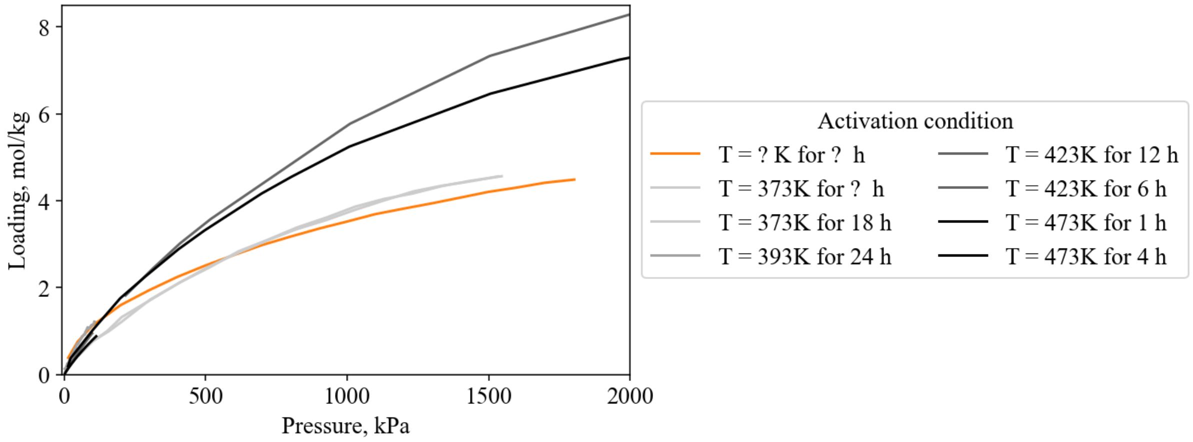

Figure S30: HKUST-1 CH_4 298K isotherm reproducibility. Darker gradient indicates higher activation temperature, with orange indicating an unknown activation temperature.

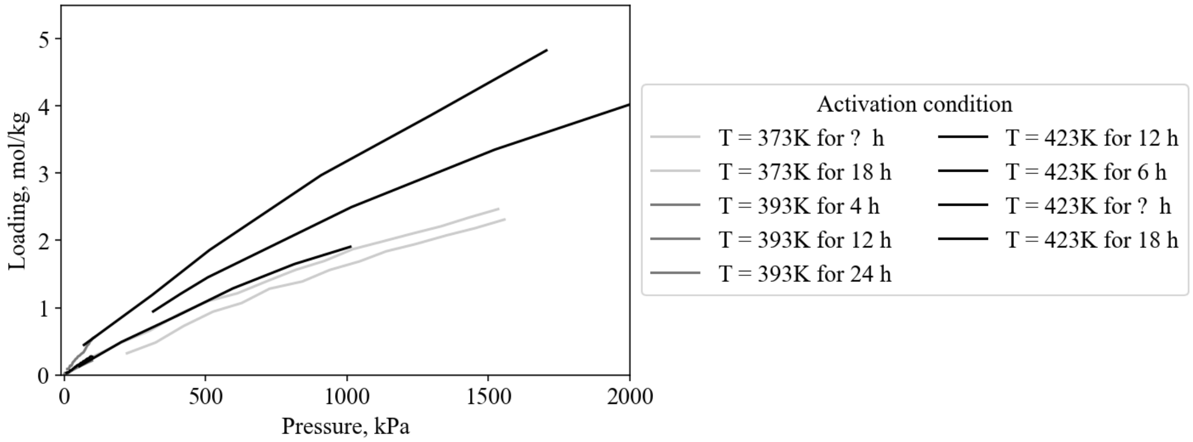

Figure S31: HKUST-1 N_2 298K isotherm reproducibility. Darker gradient indicates higher activation temperature, with orange indicating an unknown activation temperature.

5.3 UiO-66

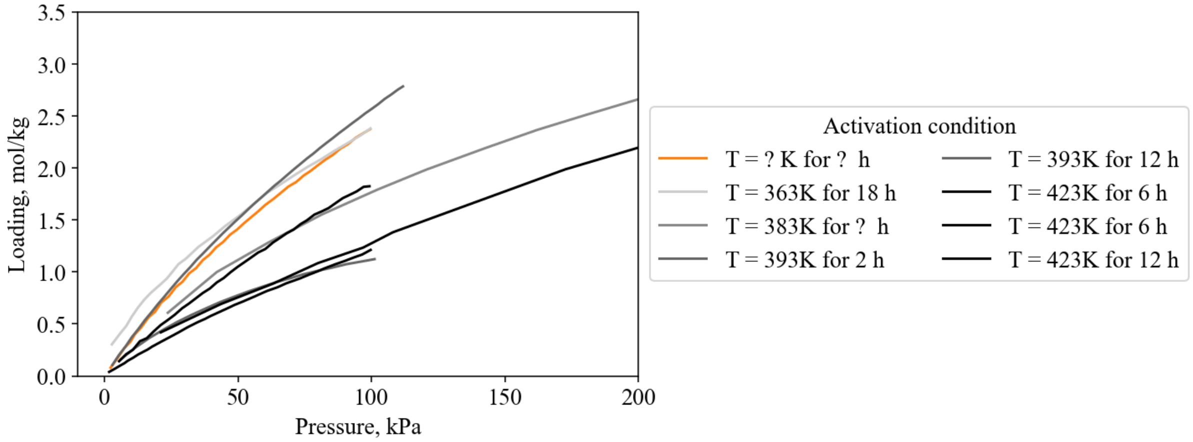

Figure S32: UiO-66 CO_2 298K isotherm reproducibility. Darker gradient indicates higher activation temperature, with orange indicating an unknown activation temperature.

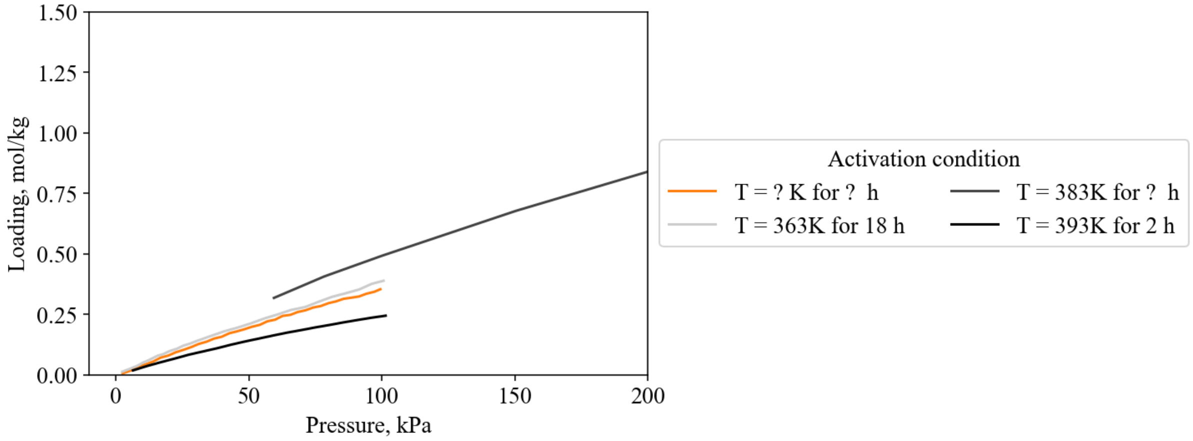

Figure S33: UiO-66 CH_4 298K isotherm reproducibility. Darker gradient indicates higher activation temperature, with orange indicating an unknown activation temperature.

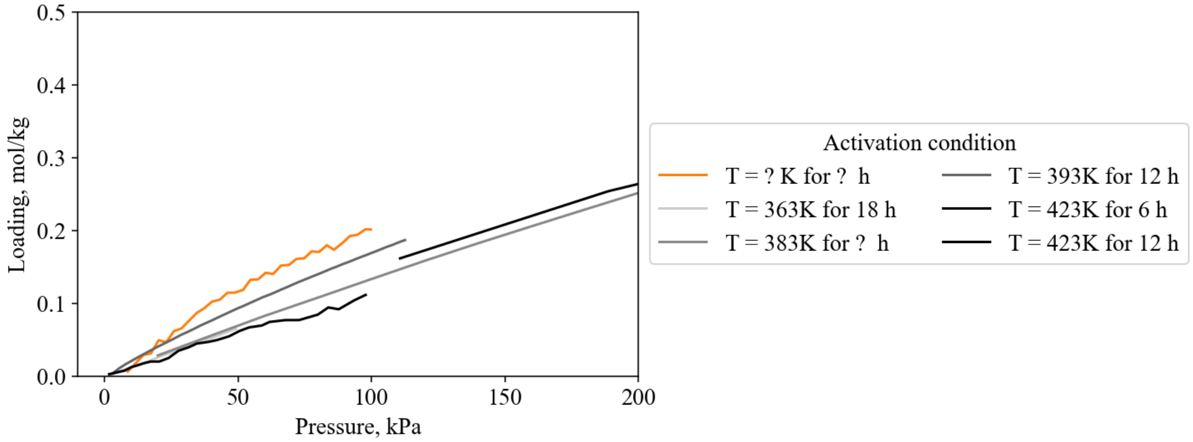

Figure S34: UiO-66 N_2 298K isotherm reproducibility. Darker gradient indicates higher activation temperature, with orange indicating an unknown activation temperature.

5.4 ZIF-8

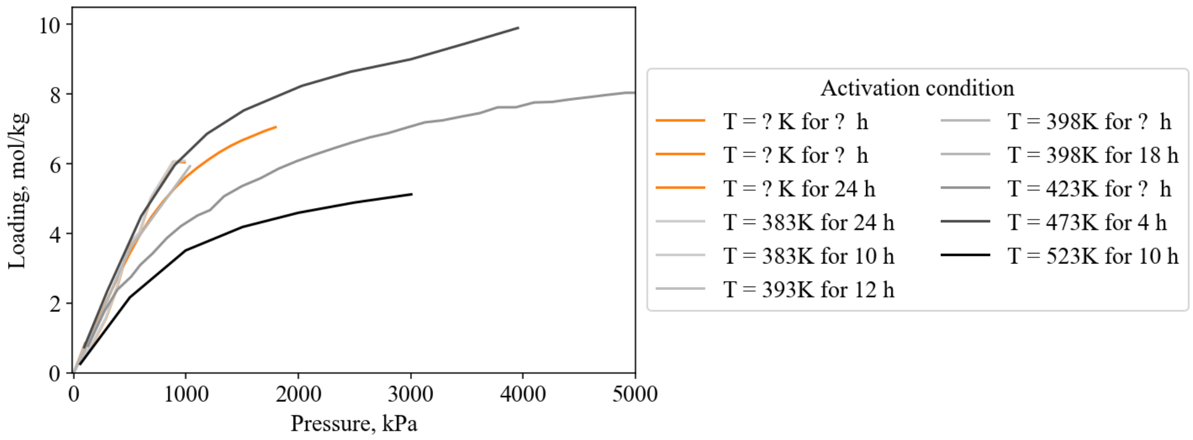

Figure S35: ZIF-8 CO_2 298K isotherm reproducibility. Darker gradient indicates higher activation temperature, with orange indicating an unknown activation temperature.

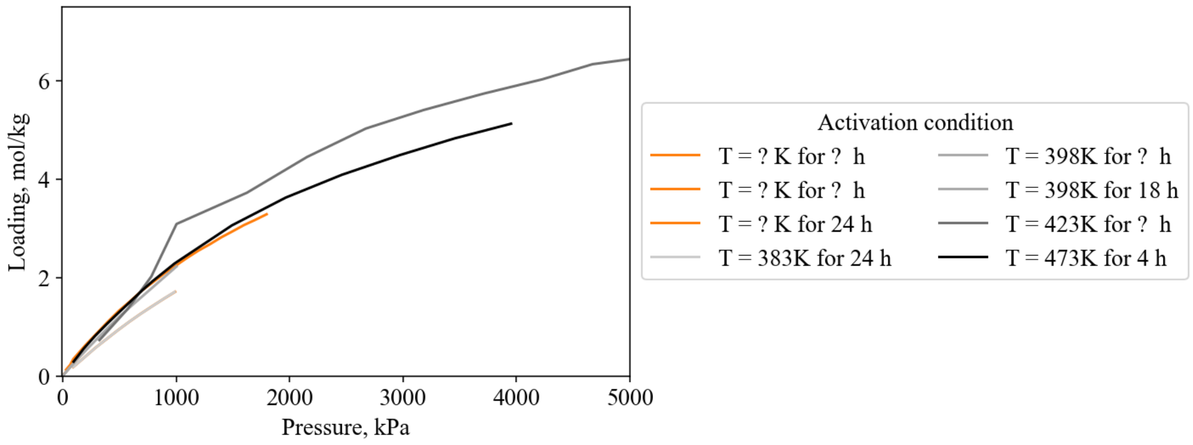

Figure S36: ZIF-8 CH_4 298K isotherm reproducibility. Darker gradient indicates higher activation temperature, with orange indicating an unknown activation temperature.

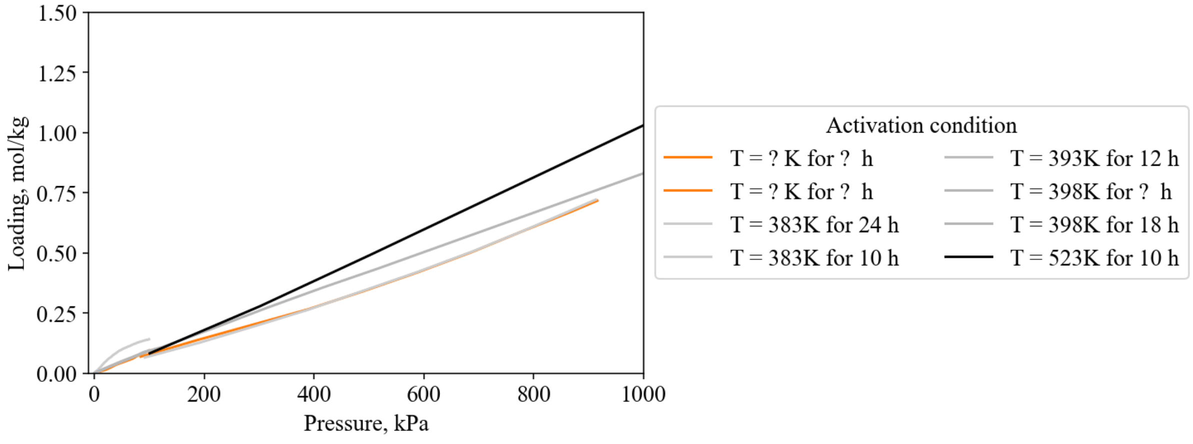

Figure S37: ZIF-8 N_2 298K isotherm reproducibility. Darker gradient indicates higher activation temperature, with orange indicating an unknown activation temperature.

6 Cost analysis

This section outlines the method used to estimate the cost of Zeolite 13X and HKUST-1. Sigma-Aldrich prices were chosen as at 5/07/2022 for the largest pack size/cheapest price per kg or L.

6.1 Zeolite 13X cost for literature example[51]

VSA adsorber vessel volume = 630 m^3

Zeolite 13X density = 750 kg/m^3

Mass of Zeolite 13X = 472.5 tonnes

Cost of Zeolite 13X quoted in paper = 5 USD/kg = 6.8USD/kg accounting for inflation.

Cost of Zeolite 13X from Alibaba = 1.5 USD/kg

Total cost of Zeolite 13X = 3.2 millionUSD or 0.7 million USD.

6.2 HKUST-1 commercial sample purchase

Mass of HKUST-1 = 472.5/4 = 118.1 tonnes (generously assuming a 4 fold increase in working capacity)

Commercial quotes for HKUST-1 = 3430 USD/kg[52] and 7200 USD/kg[53]

Total cost of HKUST-1 = 405 or 851 millionUSD

6.3 Cost to purchase synthesized material from Sigma-Aldrich

Zeolite 13X – Sigma SKU 283592-1KG = 114 USD/kg

HKUST-1 – Sigma SKU 688614-100G = 26500 USD/kg

Total cost of Zeolite 13X = 54 millionUSD

Total cost of HKUST-1 = 3130 millionUSD

6.4 Cost to purchase raw materials for synthesis from Sigma-Aldrich

HKUST-1 synthesis method, assuming 100% yield.[10]

Copper nitrate hydrate, trimesic acid and ethanol.

0.8806g trimesic acid with 24mL ethanol = 27.25mL EtOH/g_acid

$$3Cu(NO_3)_2 2H_3BTC \rightarrow Cu_3\,(BTC)_2 + 6NO_3^-$$

For 1g of HKUST-1,

Table S2: Cost (per gram) to synthesize HKUST-1 from Sigma-Aldrich raw materials.

	**Amount required (g or L)**	**Sigma USD/g or /L**	**USD/gHKUST**
**Copper nitrate (g)**	0.9302	1.224	1.139
**Trimesic acid (g)**	0.6944	0.516	0.358
**Ethanol (L)**	0.0189	47.3	0.895
			2.392

Zeolite 13X synthesis method, assuming 100% yield.[54] Bentonite mixed with NaOH powder at a 1:1.4 weight ratio.

For 1 kg of Zeolite 13X,

Table S3: Cost (per kilogram) to synthesize Zeolite 13X from Sigma-Aldrich raw materials.

	**Amount required (kg)**	**Sigma USD/kg**	**USD/kgZ13X**
**Bentonite (kg)**	0.417	1.224	28.17
**Sodium hydroxide (kg)**	0.583	0.516	38.85
			67.02

Total cost of Zeolite 13X = 32 millionUSD

Total cost of HKUST-1 = 283 millionUSD

7 References

1. Golden, T.C., and Sircar, S. (1994) Gas adsorption of silicalite. *J. Colloid Interface Sci.*, **162** (1), 182–8.
2. Scholes, C., Kentish, S., and Stevens, G. (2010) Carbon Dioxide Separation through Polymeric Membrane Systems for Flue Gas Applications. *Recent Pat. Chem. Eng.*, **1**.
3. Zhu, Z., Li, B., Liu, X., Zhang, P., Chen, S., Deng, Q., Zeng, Z., Wang, J., and Deng, Shuguang. (2021) Efficient Xe/Kr separation on two Metal-Organic frameworks with distinct pore shapes. *Sep. Purif. Technol.*, **274** (Copyright (C) 2021 American Chemical Society (ACS). All Rights Reserved.), 119132.
4. (2022) Kinetic diameter. *Wikipedia*.
5. Aguilar-Armenta, G., and Romero-Pérez, A. (2009) Adsorption of C2H4, C2H6 and CO2 on cation-exchanged clinoptilolite. *Adsorpt. Sci. Technol.*, **27** (5), 523–536.
6. Lawless, W.N., and Devries, R.C. (1964) Oxygen polarizability and point-dipole theory in the carbonate minerals. *J. Phys. Chem. Solids*, **25** (10), 1119–1124.
7. Geier, S.J., Mason, J.A., Bloch, E.D., Queen, W.L., Hudson, M.R., Brown, C.M., and Long, J.R. (2013) Selective adsorption of ethylene over ethane and propylene over propane in the metal-organic frameworks M2(dobdc) (M = Mg, Mn, Fe, Co, Ni, Zn). *Chem. Sci.*, **4** (5), 2054–2061.
8. Buckingham, A.D., Disch, R.L., and Dunmur, D.A. (1968) Quadrupole moments of some simple molecules. *J. Am. Chem. Soc.*, **90** (12), 3104–3107.
9. The Engineering ToolBox.
10. Liang, Z., Marshall, M., and Chaffee, A.L. (2009) CO2 Adsorption-Based Separation by Metal Organic Framework (Cu-BTC) versus Zeolite (13X). *Energy Fuels*, **23** (5), 2785–2789.
11. Streb, A., and Mazzotti, Marco. (2021) Adsorption for efficient low carbon hydrogen production: part 1-adsorption equilibrium and breakthrough studies for H2/CO2/CH4 on zeolite 13X. *Adsorption*, **27** (4), 541–558.
12. Lawson, S., Newport, K., Al-Naddaf, Q., Ameh, A.E., Rownaghi, A.A., Petrik, L.F., and Rezaei, Fateme. (2021) Binderless zeolite monoliths production with sacrificial biopolymers. *Chem. Eng. J. Amst. Neth.*, **407** (Copyright (C) 2021 American Chemical Society (ACS). All Rights Reserved.), 128011.
13. Morales-Ospino, R., Santiago, R.G., Siqueira, R.M., de Azevedo, D.C.S., and Bastos-Neto, Moises. (2020) Assessment of CO2 desorption from 13X zeolite for a prospective TSA process. *Adsorption*, **26** (5), 813–824.
14. Golipour, H., Mokhtarani, B., Mafi, M., Khadivi, M., and Godini, H.Reza. (2019) Systematic Measurements of CH4 and CO2 Adsorption Isotherms on Cation-Exchanged Zeolites 13X. *J. Chem. Eng. Data*, **64** (10), 4412–4423.
15. Estupinan Perez, L., Sarkar, P., and Rajendran, Arvind. (2019) Experimental validation of multi-objective optimization techniques for design of vacuum swing adsorption processes. *Sep. Purif. Technol.*, **224** (Copyright (C) 2021 American Chemical Society (ACS). All Rights Reserved.), 553–563.
16. Wilkins, N.S., and Rajendran, Arvind. (2019) Measurement of competitive CO2 and N2 adsorption on Zeolite 13X for post-combustion CO2 capture. *Adsorption*, **25** (2), 115–133.
17. Wynnyk, K.G., Hojjati, B., and Marriott, R.A. (2018) High-Pressure Sour Gas and Water Adsorption on Zeolite 13X. *Ind. Eng. Chem. Res.*, **57** (45), 15357–15365.
18. Epiepang, F.E., Li, J., Liu, Y., and Yang, R.T. (2016) Low-pressure performance evaluation of CO2, H2O and CH4 on Li-LSX as a superior adsorbent for air prepurification. *Chem. Eng. Sci.*, **147** (Copyright (C) 2021 American Chemical Society (ACS). All Rights Reserved.), 100–108.
19. Gibson, J.A.A., Mangano, E., Shiko, E., Greenaway, A.G., Gromov, A.V., Lozinska, M.M., Friedrich, D., Campbell, E.E.B., Wright, P.A., and Brandani, Stefano. (2016) Adsorption Materials and Processes for Carbon Capture from Gas-Fired Power Plants: AMPGas. *Ind. Eng. Chem. Res.*, **55** (13), 3840–3851.
20. Hefti, M., Marx, D., Joss, L., and Mazzotti, Marco. (2015) Adsorption equilibrium of binary mixtures of carbon dioxide and nitrogen on zeolites ZSM-5 and 13X. *Microporous Mesoporous Mater.*, **215** (Copyright (C) 2021 American Chemical Society (ACS). All Rights Reserved.), 215–228.

21. Krishnamurthy, S., Rao, V.R., Guntuka, S., Sharratt, P., Haghpanah, R., Rajendran, A., Amanullah, M., Karimi, I.A., and Farooq, Shamsuzzaman. (2014) CO2 capture from dry flue gas by vacuum swing adsorption: A pilot plant study. *AIChE J.*, **60** (5), 1830–1842.
22. McEwen, J., Hayman, J.-D., and Ozgur Yazaydin, A. (2013) A comparative study of CO2, CH4 and N2 adsorption in ZIF-8, Zeolite-13X and BPL activated carbon. *Chem. Phys.*, **412**, 72–76.
23. Bezerra, D.P., Oliveira, R.S., Vieira, R.S., Cavalcante, C.L., Jr., and Azevedo, D.C.S. (2011) Adsorption of CO2 on nitrogen-enriched activated carbon and zeolite 13X. *Adsorption*, **17** (1), 235–246.
24. Bao, Z., Yu, L., Ren, Q., Lu, X., and Deng, Shuguang. (2010) Adsorption of CO2 and CH4 on a magnesium-based metal organic framework. *J. Colloid Interface Sci.*, **353** (2), 549–556.
25. Liang, Z., Marshall, M., and Chaffee, A.L. (2009) Comparison of Cu-BTC and zeolite 13X for adsorbent based CO2 separation. *Energy Procedia*, **1** (1), 1265–1271.
26. Reljic, S., Broto-Ribas, A., Cuadrado-Collados, C., Jardim, E.O., Maspoch, D., Imaz, I., and Silvestre-Albero, Joaquin. (2020) Structural Deterioration of Well-Faceted MOFs upon H2S Exposure and Its Effect in the Adsorption Performance. *Chem. - Eur. J.*, **26** (71), 17110–17119.
27. Choi, C., Kadam, R.L., Gaikwad, S., Hwang, K.-S., and Han, Sangil. (2020) Metal organic frameworks immobilized polyacrylonitrile fiber mats with polyethyleneimine impregnation for CO2 capture. *Microporous Mesoporous Mater.*, **296** (Copyright (C) 2021 American Chemical Society (ACS). All Rights Reserved.), 110006.
28. Wu, Y., Lv, Z., Zhou, X., Peng, J., Tang, Y., and Li, Zhong. (2019) Tuning secondary building unit of Cu-BTC to simultaneously enhance its CO2 selective adsorption and stability under moisture. *Chem. Eng. J. Amst. Neth.*, **355** (Copyright (C) 2021 American Chemical Society (ACS). All Rights Reserved.), 815–821.
29. Iriowen, E.M., and Goudy, Andrew. (2016) A comparison of gas adsorption on metalorganic frameworks using a sticking factor concept. *Int. J. Adv. Res.*, **4** (8), 362–379.
30. Hu, Z., Wang, Y., Farooq, S., and Zhao, Dan. (2017) A highly stable metal-organic framework with optimum aperture size for CO2 capture. *AIChE J.*, **63** (9), 4103–4114.
31. Sezginel, K.B., Keskin, S., and Uzun, Alper. (2016) Tuning the Gas Separation Performance of CuBTC by Ionic Liquid Incorporation. *Langmuir*, **32** (4), 1139–1147.
32. Rios, R.B., Correia, L.S., Bastos-Neto, M., Torres, A.E.B., Hatimondi, S.A., Ribeiro, A.M., Rodrigues, A.E., Cavalcante, C.L., Jr., and de Azevedo, D.C.S. (2014) Evaluation of carbon dioxide-nitrogen separation through fixed bed measurements and simulations. *Adsorption*, **20** (8), 945–957.
33. Zhang, H., Xiao, R., Song, M., Shen, D., and Liu, Jian. (2014) Hydrogen production from bio-oil by chemical looping reforming - Characteristics of the synthesized metal organic frameworks for CO2 removal. *J. Therm. Anal. Calorim.*, **115** (2), 1921–1927.
34. Xiang, Z., Peng, X., Cheng, X., Li, X., and Cao, Dapeng. (2011) CNT@Cu3(BTC)2 and Metal-Organic Frameworks for Separation of CO2/CH4 Mixture. *J. Phys. Chem. C*, **115** (40), 19864–19871.
35. Karra, J.R., and Walton, K.S. (2010) Molecular simulations and experimental studies of CO2, CO, and N2 adsorption in metal-organic frameworks. *J. Phys. Chem. C*, **114** (37), 15735–15740.
36. Chen, C., Li, X., Zou, W., Wan, H., Dong, L., and Guan, Guofeng. (2021) Structural modulation of UiO-66-NH2 metal-organic framework via interligands cross-linking: Cooperative effects of pore diameter and amide group on selective CO2 separation. *Appl. Surf. Sci.*, **553** (Copyright (C) 2021 American Chemical Society (ACS). All Rights Reserved.), 149547.
37. Mutyala, S., Jonnalagadda, M., and Ibrahim, S.M. (2021) Effect of modification of UiO-66 for CO2 adsorption and separation of CO2/CH4. *J. Mol. Struct.*, **1227** (Copyright (C) 2021 American Chemical Society (ACS). All Rights Reserved.), 129506.
38. Cao, Y., Zhang, H., Song, F., Huang, T., Ji, J., Zhong, Q., Chu, W., and Xu, Qi. (2018) UiO-66-NH2/GO composite: synthesis, characterization and CO2 adsorption performance. *Materials*, **11** (4), 589/1-589/15.
39. Edubilli, S., and Gumma, Sasidhar. (2019) A systematic evaluation of UiO-66 metal organic framework for CO2/N2 separation. *Sep. Purif. Technol.*, **224** (Copyright (C) 2021 American Chemical Society (ACS). All Rights Reserved.), 85–94.

40. Wu, W., Li, Z., Chen, Y., and Li, Wanbin. (2019) Polydopamine-Modified Metal-Organic Framework Membrane with Enhanced Selectivity for Carbon Capture. *Environ. Sci. Technol.*, **53** (7), 3764–3772.
41. Tovar, T.M., Iordanov, I., Sava Gallis, D.F., and De Coste, J.B. (2018) Enhancing Van der Waals Interactions of Functionalized UiO-66 with Non-polar Adsorbates: The Unique Effect of para Hydroxyl Groups. *Chem. - Eur. J.*, **24** (8), 1931–1937.
42. Valekar, A.H., Cho, K.-H., Lee, U.-H., Lee, J.S., Yoon, J.W., Hwang, Y.K., Lee, S.G., Cho, S.J., and Chang, J.-San. (2017) Shaping of porous metal-organic framework granules using mesoporous ρ-alumina as a binder. *RSC Adv.*, **7** (88), 55767–55777.
43. Cmarik, G.E., Kim, M., Cohen, S.M., and Walton, K.S. (2012) Tuning the Adsorption Properties of UiO-66 via Ligand Functionalization. *Langmuir*, **28** (44), 15606–15613.
44. Awadallah-F, A., Hillman, F., Al-Muhtaseb, S.A., and Jeong, H.-Kwon. (2019) Adsorption of carbon dioxide, methane, and nitrogen gases onto ZIF compounds with zinc, cobalt, and zinc/cobalt metal centers. *J. Nanomater.*, (Copyright (C) 2021 American Chemical Society (ACS). All Rights Reserved.), 6130152.
45. Awadallah-F, A., Hillman, F., Al-Muhtaseb, S.A., and Jeong, H.-Kwon. (2019) Adsorption Equilibrium and Kinetics of Nitrogen, Methane and Carbon Dioxide Gases onto ZIF-8, Cu10%/ZIF-8, and Cu30%/ZIF-8. *Ind. Eng. Chem. Res.*, **58** (16), 6653–6661.
46. Zeeshan, M., Keskin, S., and Uzun, Alper. (2018) Enhancing CO2/CH4 and CO2/N2 separation performances of ZIF-8 by post-synthesis modification with [BMIM][SCN]. *Polyhedron*, **155** (Copyright (C) 2021 American Chemical Society (ACS). All Rights Reserved.), 485–492.
47. Koyuturk, B., Altintas, C., Kinik, F.P., Keskin, S., and Uzun, Alper. (2017) Improving Gas Separation Performance of ZIF-8 by [BMIM][BF4] Incorporation: Interactions and Their Consequences on Performance. *J. Phys. Chem. C*, **121** (19), 10370–10381.
48. Zhu, J., Jiang, L., Dai, C., Yang, N., and Lei, Zhigang. (2015) Gas adsorption in shaped zeolitic imidazolate framework-8. *Chin. J. Chem. Eng.*, **23** (8), 1275–1282.
49. Xian, S., Xu, F., Ma, C., Wu, Y., Xia, Q., Wang, H., and Li, Zhong. (2015) Vapor-enhanced CO2 adsorption mechanism of composite PEI@ZIF-8 modified by polyethyleneimine for CO2/N2 separation. *Chem. Eng. J. Amst. Neth.*, **280** (Copyright (C) 2021 American Chemical Society (ACS). All Rights Reserved.), 363–369.
50. Zhang, Z., Xian, S., Xi, H., Wang, H., and Li, Zhong. (2011) Improvement of CO2 adsorption on ZIF-8 crystals modified by enhancing basicity of surface. *Chem. Eng. Sci.*, **66** (20), 4878–4888.
51. Ho, M.T., Allinson, G.W., and Wiley, D.E. (2008) Reducing the Cost of CO2 Capture from Flue Gases Using Pressure Swing Adsorption. *Ind. Eng. Chem. Res.*, **47** (14), 4883–4890.
52. Metal Organic Framework Solutions | MOF Technologies UK.
53. Metal-Organic Frameworks (MOFs) production. *novoMOF.*
54. Chen, C., Park, D.-W., and Ahn, W.-S. (2014) CO2 capture using zeolite 13X prepared from bentonite. *Appl. Surf. Sci.*, **292**, 63–67.
