# Supplementary figures and images for: An Upper Bound Visualization of Design Trade‐Offs in Adsorbent Materials for Gas Separations: CO2, N2, CH4, H2, O2, Xe, Kr, and Ar Adsorbents
Source: Adv Sci (Weinh). 2023 Jan 16;10(8):2206437. doi: 10.1002/advs.202206437 (PMC10015871; doi:10.1002/advs.202206437)

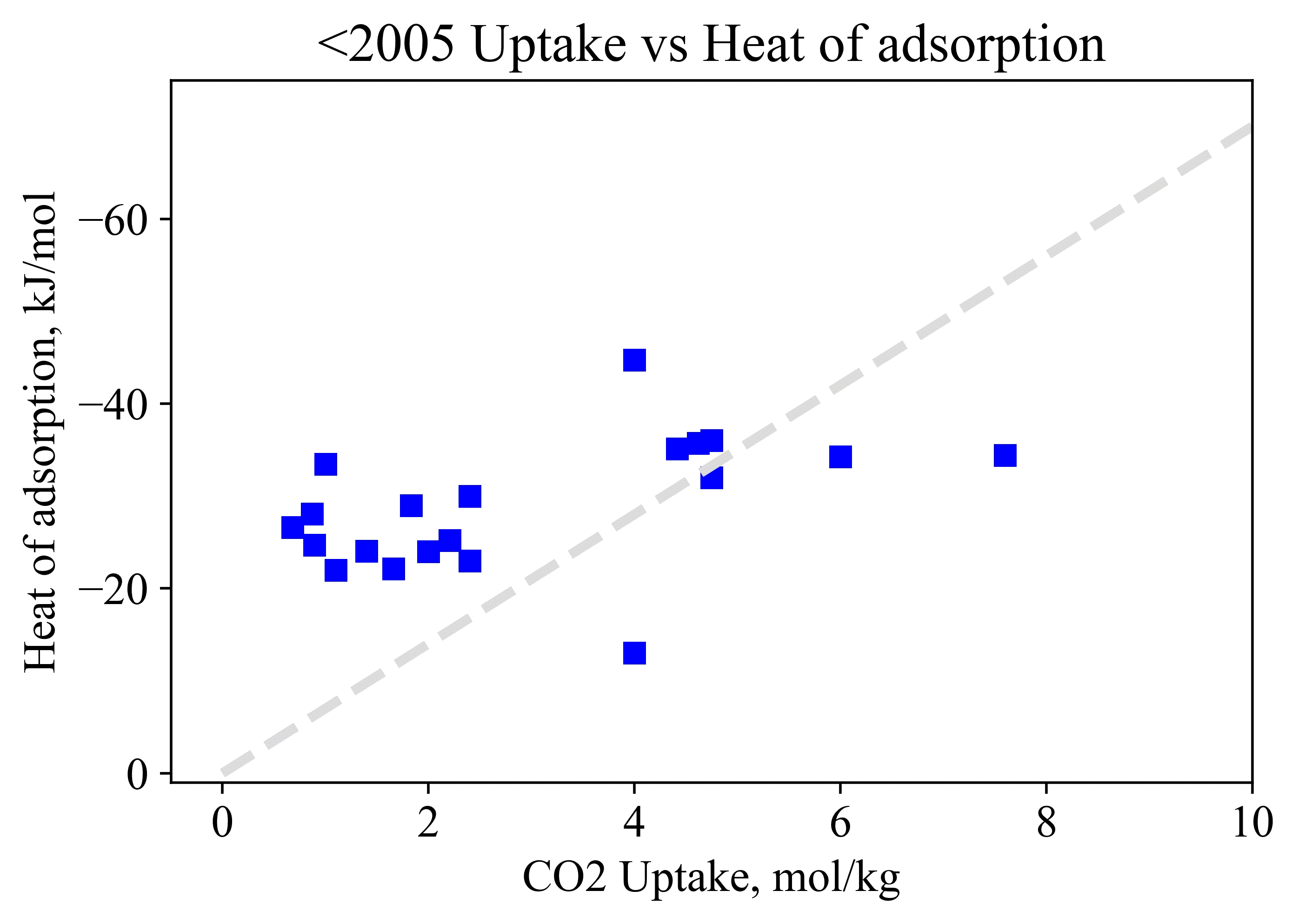

Supplement: Supplementary file 2 — Supporting Information [file ADVS-10-2206437-s001.gif]

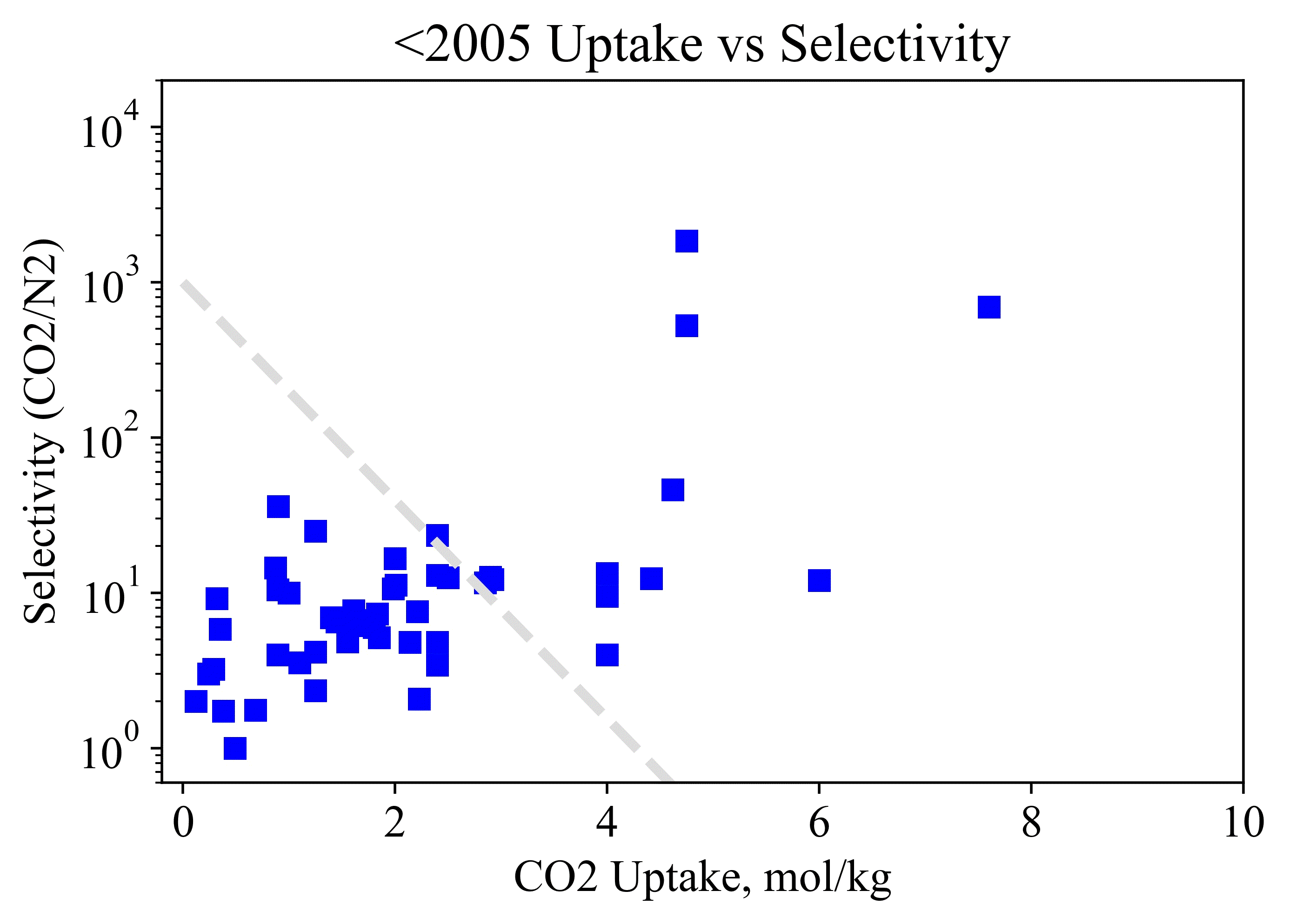

Supplement: Supplementary file 3 — Supporting Information [file ADVS-10-2206437-s004.gif]

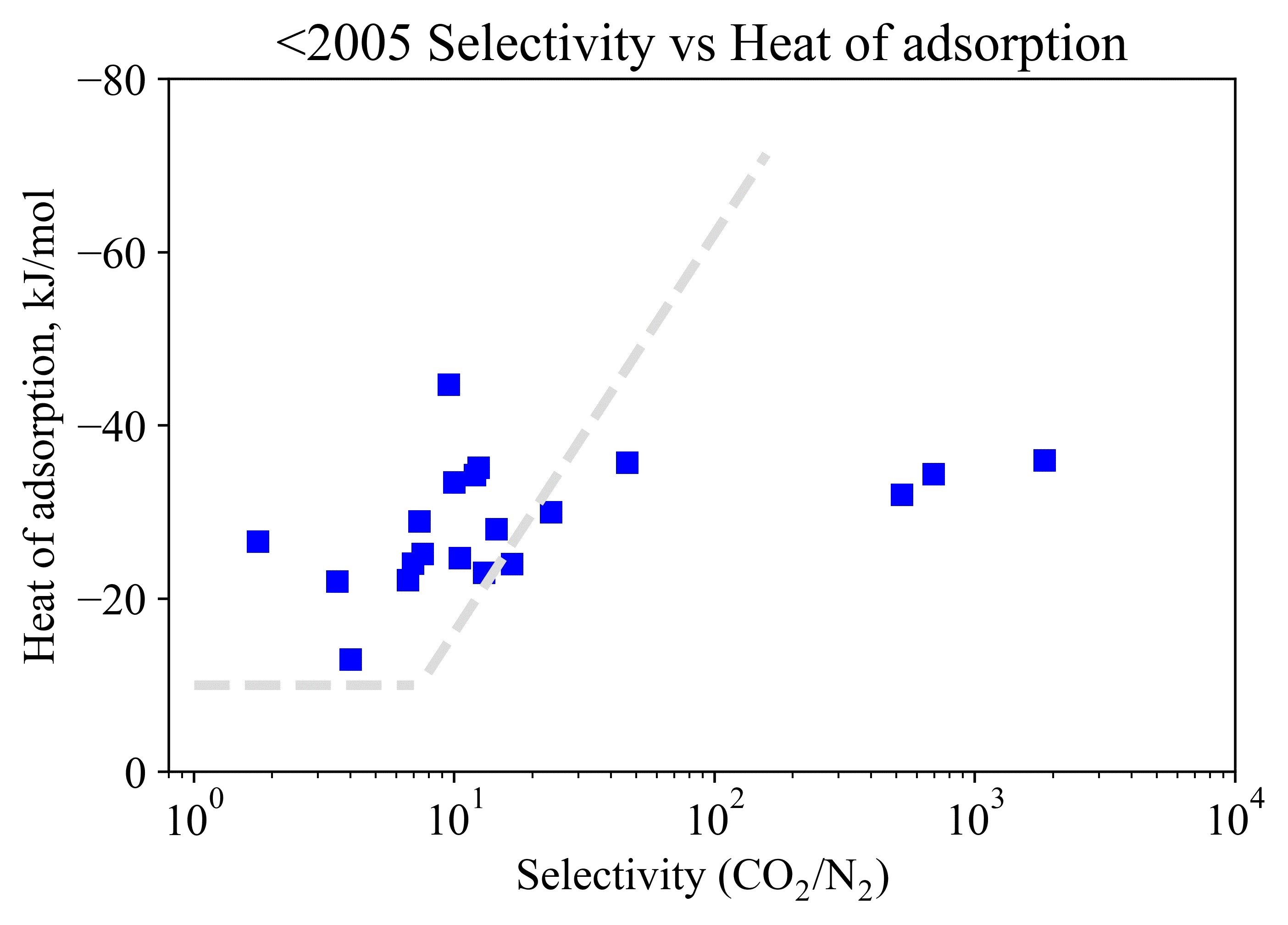

Supplement: Supplementary file 4 — Supporting Information [file ADVS-10-2206437-s003.gif]
